# Convergence of Afrotherian and Laurasiatherian Ungulate-Like Mammals: First Morphological Evidence from the Paleocene of Morocco

**DOI:** 10.1371/journal.pone.0157556

**Published:** 2016-07-06

**Authors:** Emmanuel Gheerbrant, Andrea Filippo, Arnaud Schmitt

**Affiliations:** CR2P –Centre de Recherches sur la Paléobiodiversité et les Paléoenvironnements, UMR 7207, Muséum National d'Histoire Naturelle, CNRS, UPMC, Sorbonne Universités. MNHN, CP38, Paris, France; New York Institute of Technology, UNITED STATES

## Abstract

Molecular-based analyses showed that extant “ungulate” mammals are polyphyletic and belong to the two main clades Afrotheria (Paenungulata) and Laurasiatheria (Euungulata: Cetartiodactyla-Perissodactyla). However, paleontological and neontological studies hitherto failed to demonstrate the morphological convergence of African and Laurasian “ungulate” orders. They support an “Altungulata” group including the Laurasian order Perissodactyla and the African superorder Paenungulata and characterized especially by quadritubercular and bilophodont molars adapted for a folivorous diet. We report new critical fossils of one of the few known African condylarth-like mammal, the enigmatic *Abdounodus* from the middle Paleocene of Morocco. They show that *Abdounodus* and *Ocepeia* display key intermediate morphologies refuting the homology of the fourth main cusp of upper molars in Paenungulata and Perissodactyla: Paenungulates unexpectedly have a metaconule-derived pseudohypocone, instead of a cingular hypocone. Comparative and functional dental anatomy of *Abdounodus* demonstrates indeed the convergence of the quadritubercular and bilophodont pattern in “ungulates”. Consistently with our reconstruction of the structural evolution of paenungulate bilophodonty, the phylogenetic analysis relates *Abdounodus* and *Ocepeia* to Paenungulata as stem taxa of the more inclusive new clade Paenungulatomorpha which is distinct from the Perissodactyla and Anthracobunidae. *Abdounodus* and *Ocepeia* help to identify the first convincing synapomorphy within the Afrotheria–i.e., the pseudohypocone–that demonstrates the morphological convergence of African and Laurasian ungulate-like placentals, in agreement with molecular phylogeny. *Abdounodus* and *Ocepeia* are the only known representatives of the early African ungulate radiation predating the divergence of extant paenungulate orders. Paenungulatomorpha evolved in Africa since the early Tertiary independently from laurasiatherian euungulates and “condylarths” such as apheliscids. The rapid early Tertiary radiation of the Afrotheria and Paenungulatomorpha, as illustrated by the Paleocene Moroccan mammals, is concurrent with that of the Laurasiatheria in a general, explosive mammal evolution in both the South and North Tethyan continents following the K/Pg event.

## Introduction

The higher-level, interordinal relationships of the placental mammals is currently the subject of important research programs and heated discussions that involve several disciplines (e.g., paleontology and molecular phylogenetics) with different approaches (e.g., maximum likelihood and parsimony phylogenetic analyses) and different objects of studies such as genomic and discrete phenotypic data. Over the last twenty years, molecular studies have identified several new major placental clades [1–5], some of which being either unsupported or conflicting with morphological-based phylogenetic studies that include both extant and extinct taxa. This is especially true for the ungulate-like placentals of debated phylogenetic relationships [6–9]. Here we describe and analyze new morphological and fossil data that reconcile the paleontological, paleobiogeographical and molecular data on the lophodont Laurasian and African ungulate-like placentals. Our study enlightens the significance of early and stem taxa in providing factual examples of intermediate morphologies and character combination that were hitherto unsuspected when based on exclusively modern taxa. They document the history of character evolution, especially their ancestral states, and help to clarify the morphological phylogenetic signal, even for characters such as dental traits that are reputedly prone to homoplasy. Our study, based on parsimony and functional analyses, allow the reconstruction of the hypothetical ancestral morphotype of the dental pattern of the African “ungulates”, and its subsequent character states. It sheds new light on the homology of some key dental characters (quadrituberculy and bilophodonty) and demonstrates their convergent evolution with the Laurasian “ungulates”. The phylogenetic study, based exclusively on discrete morphological data, yields a tree topology that is congruent with molecular-based phylogeny at high-rank level.

Ungulate-like mammal orders evolved since the early Tertiary in both Laurasia and Africa, and all were included in the taxon “Ungulata”Linnaeus, 1766 [10] in the Simpson’s classification of mammals [11]. Two major morphological “ungulate” groups were distinguished: the Cetartiodactyla, and the Perissodactyla plus Paenungulata Simpson, 1945 [11] that includes living orders Proboscidea, Hyracoidea, and Sirenia. The Laurasian order Perissodactyla and the African paenungulate orders all share several features and most remarkably a bilophodont molar pattern [12]. Such a group of lophodont “ungulates”, which also includes extinct relatives such as Anthracobunidae and Desmostylia, was named “Altungulata” [13–14]. Phylogenetic studies based on molecular data showed that “ungulates” are in fact polyphyletic and belong to two major clades, the Laurasiatheria (including the Artiodactyla and Perissodactyla as Euungulata) and the Afrotheria (including the Paenungulata). However, the paleontological and neontological studies hitherto failed to demonstrate the convergence of the bilophodont pattern in Afrotheria and Laurasiatheria and relatedly the diphyly of the “Altungulata” morphological taxon that includes the Paenungulata and Perissodactyla. Here, we report the discovery of a new key material of the poorly known condylarth-like genus *Abdounodus* from the Selandian of the Ouled Abdoun phosphate basin (Morocco), which sheds new light on the origin and early evolution of the bilophodonty in Paenungulata, and shows its independent evolution from that of the laurasiatherian euungulates such as the Perissodactyla.

The phosphate levels of the Ouled Abdoun basin are famous for their very rich marine vertebrate fauna that extends across the Cretaceous-Paleogene and Paleocene-Eocene boundaries [15–16]. They have also yielded fossils of early placental mammals that document the earliest endemic African mammals, and especially basalmost crown paenungulates, such as the proboscideans *Phosphatherium* (early Ypresian, ca. 55 ma) and *Eritherium* (Selandian, ca. 60 ma) [17–20]. The Selandian mammal level of the Ouled Abdoun basin also yielded the very few known African condylarth-like taxa. They include two genera possibly related to paenungulates: *Ocepeia*, recently documented by its skull [21], and the enigmatic *Abdounodus* only known by its lower dentition [22–23]. We report here the first discovery of the upper dentition of *Abdounodus* that provides new and key morphological data on the ancestral dental morphotype and the early evolution of the Paenungulata.

The Paleocene fossilerous level from the Ouled Abdoun basin that yields the mammals *Abdounodus hamdii*, *Ocepeia daouiensis* and *Eritherium azzouzorum* is dated as Selandian on the basis of stable isotope chemostratigraphical data recently published [24–25].

## Material, Method of Study

### Acronyms of paleontological collections

*OCP DEK/GE*: Collections of the Office Chérifien des Phosphates, Khouribga, Morocco.

*MNHN*.*F*: Collections of the Muséum National d'Histoire Naturelle (F: Paleontology), Paris, France. *MHNM*.*KHG*: collections of the Natural History Museum of Marrakech, Morocco (KHG: localities of the Ouleb Abdoun basin, Khouribga area).

### Paleontological Ethics Statements

The studied specimen MHNM.KHG.154 is permanently reposited in the collections of the Natural History Museum of Marrakech, in Marrakech, Morocco. The specimens OCP DEK/GE 308 and OCP DEK/GE 310 are permanently reposited in the collections of the Office Chérifien des Phosphates, Khouribga, Morocco. The specimens MNHN.F PM35 and MNHN.F PM92 are permanently reposited in the collections of the Muséum National d'Histoire Naturelle, Paris France.

### Field work

No permits were required for the described study, which complied with all relevant regulations.

### Measurements

Measurements are provided in millimeters (mm).

### CT Scan, 3D modelisation, softwares

MHNM.KHG.154 and MNHN.F PM92 were subjected to X-ray Computed Tomographic (CT) imaging at the AST-RX platform of the MNHN, using a GE Sensing and Inspection Technologies phoenix|x-ray v|tome|x L240-180 CT scanner. We used the microfocus RX source 240kV/320W, detector 400 × 400 mm with a matrix of 2024 pixels (pixel size: 200x200μm). Data were reconstructed using datos|x reconstruction software (Phoenix|x-ray, release 2.0) and then exported into a 16 bits TIFF image stack. The 3D models were reconstructed from the CT scans using the computer programs Materialise Mimics Innovation Suite 18.0 Research Edition (x64), and Maxon Cinema 4D R15. Scale bar = 10 mm.

## Results

### Systematic Paleontology

Infraclass Placentalia Owen, 1837

Supercohort Afrotheria Stanhope, Waddell, Madsen, De Jong, Hedges, Cleven, Kao, Springer, 1997

Magnorder **Paenungulatomorpha** nov. Gheerbrant

The magnorder Paenungulatomorpha is erected here to include the crown superorder Paenungulata Simpson, 1945 (Hyracoidea, Proboscidea, Sirenia, Embrithopoda,? Desmostylia) and its stem relatives such as *Ocepeia* and *Abdounodus* that have transitional molar morphology to bunodonty, quadrituberculy and bilophodonty. Paenungulatomorphans are characterized by a mandibular retromolar fossa, the absence of hypocone, an ectoloph selenodont and linked to strong styles such as mesostyle in basal taxa, and a more or less developed pseudohypocone. Early non-specialized paenungulates lack a paraconule and postcingulum, and a primitive small and labial metaconule.

Family *incertae sedis* (nov.)

*Abdounodus hamdii* Gheerbrant & Sudre, 2001

#### Revised diagnosis

Lower dentition: see Gheerbrant (2010) [23]. Upper dentition: P^3^ is simplified and narrow with small protocone, whereas P^4^ is well developed transversely with a large protocone. P^3^ and P^4^ lack a metacone. Both teeth have a well developed postprotocrista and postcingulum (protofossa closed distally). The upper molars are very bunodont and have strong and bulbous styles, and a selenodont ectoloph linked to the mesostyle; they are characterized by several features related to an incipient quadritubercular and bunodont-lophodont pattern: metaconule enlarged and lingually shifted close to protocone transverse level, and forming an incipient but distinct pseudohypocone; small interloph marked by the absence of postprotocrista; mesostyle, premetacrista, metacone and metaconule more or less aligned transversely (incipient metaloph). Upper molars lack the paraconule and postcingulum (vestigial).

#### New referred material

MHNM.KHG.154: left and right maxillaries of the same individual preserving respectively M^3-1^, P^4-3^, and M^3-1^, P^4^; collections of the Natural History Museum of Marrakech, Morocco; casts (PM95) deposited in MNHN collections, and CT scans archived in MNHN databases. MNHN.F PM92: left dentary bearing M_1_- P_4_, broken P_3_ and roots of M_3_, M_2_, and of one anterior premolar or C_1_.

## Dental Morphological and Functional Study of *Abdounodus*

### Description of the new material of *Abdounodus hamdii*

#### Maxillary and upper dentition (Figs 1, 2 and S1)

Here we describe the first known remains of the upper jaw and its dentition of *Abdounodus hamdii*.120-

**Fig 1:**
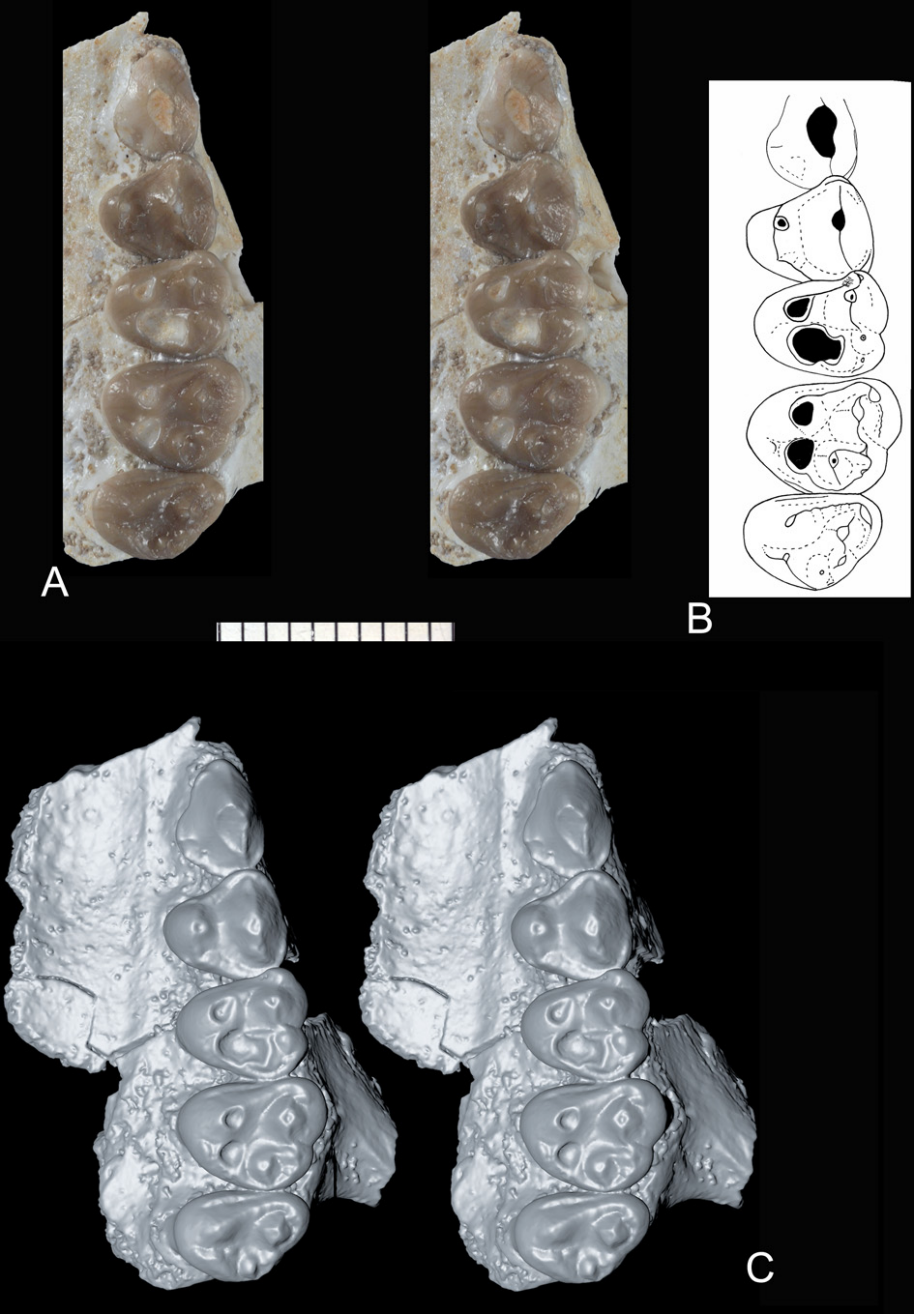
*Abdounodus hamdii*, Selandian (Phosphate level IIa) of the Ouled Abdoun Basin, Morocco. MHNM.KHG.154 (collections of the Natural History Museum of Marrakech), left maxillary of preserving P^3-4^, M^1-3^; (A) strereophotographic pair of P^3-4^, M^1-3^ in occlusal view; (B) occlusal sketch; (C) strereophotographic pair of 3D models reconstructed from CT scans in occlusal view. The 3D models of MHNM.KHG.154 were reconstructed by F. Goussard (MNHN) from the CT scans using the computer programs Materialise Mimics Innovation Suite 18.0 Research Edition (x64), and Maxon Cinema 4D R15. Scale bar = 10 mm.

**Fig 2:**
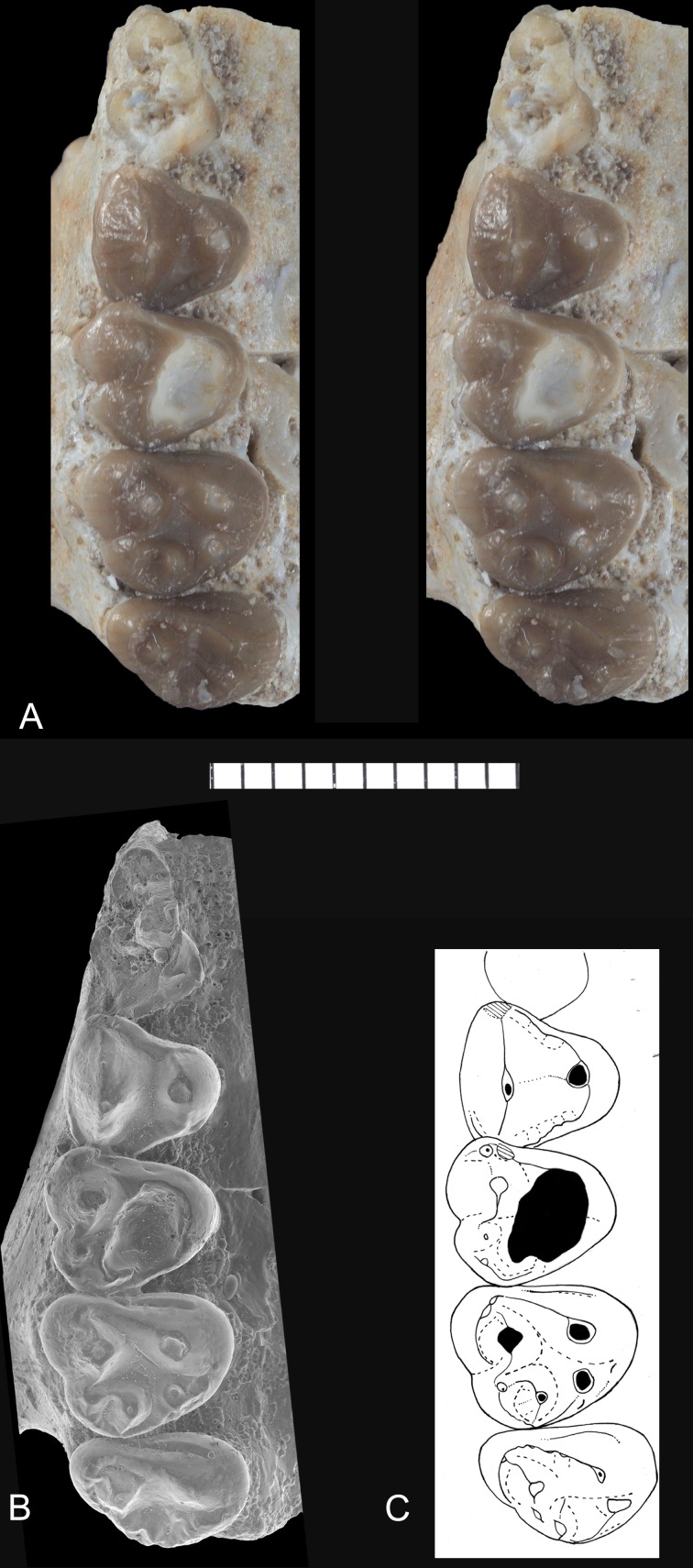
*Abdounodus hamdii*, Selandian (Phosphate level IIa) of the Ouled Abdoun Basin, Morocco. MHNM.KHG.154 (collections of the Natural History Museum of Marrakech), right maxillary of *Abdounodus hamdii* preserving P^3-4^, M^1-3^; (A) strereophotographic pair of P^3-4^, M^1-3^ in occlusal view; B, s.e.m. photograph of occlusal view; (C) occlusal sketch. Scale bar = 10 mm.

The maxillary is partially preserved in MHNM.KHG.154. It shows a small infraorbital foramen that is located above the distal part of P^3^. The zygomatic arch is rooted above M^2^, more anteriorly than in *Ocepeia*. The palatine-maxillary suture extends anteriorly at M^1^-M^2^ level. The posterior opening of the infraorbital foramen in the orbital fossa is above M^1^, which suggests that the orbit anterior rim was above M^1^. This also corresponds to the position of the anterior maxillary–jugal suture.

*Upper molars*: The general morphology is markedly bunodont, with an inflated crown and bulbous cusps (Figs 1 and 2). This is associated with an extensive abrasion wear that excavates the lingual cusps deep in the dentine, and with robust and long tooth roots. The occlusal outline of the molars, although rounded, is sub-triangular, with the lingual region slightly shorter than the labial one. The enamel is slightly wrinkled, especially at the apex of the crests and the cingula as seen in unworn teeth. M^2^ is larger than M^1^ and M^3^, which are comparable in size.

The ectoflexus is deeper on the labial side of the molar just mesial to the transverse level of the mesostyle, and more so in M^1-2^. The parastylar lobe is voluminous and larger and more salient labially than the metastylar lobe in M^2^ and M^3^. The ectocinglum is inflated and bears well developed mesostyle and parastyle; it is also crenulated with presence of small cuspules, especially in its posterior part, in front of the metacone. The mesostyle is inflated and bulbous. It is larger than the parastyle. Its size decreases from M^1^ to M^3^. It is much closer longitudinally to the metacone than to the paracone. The parastyle is located more mesially than labially, so that the preparacrista is mainly mesio-distal (very short residual labial segment). The W-shaped ectoloph is selenodont (= dilambdodonty) and it is linked to the mesostyle. The stylar shelf is narrow and the ectoloph extends labially very briefly. Because of the distal position of the mesostyle, the premetacrista is mostly transverse, whereas the postparacrista is mostly mesio-distal. The postmetacrista is present and more labially (transversely) oriented than the preparacrista; it joins the ectocingulum at the disto-labial angle of the tooth (M^1-2^).

The paracone is markedly more labial that the metacone, so that the paracone is in contact with the ectocingulum, and the metacone is separated from the ectocingulum by a small fovea (narrower in M^1^) on the stylar shelf. The mesostyle, the premetacrista, the metacone and the metaconule are aligned transversely, foreshadowing an incipient metaloph similar to that of the embrithopods, primitive hyracoids and proboscideans. A long precingulum extends from protocone to paracone levels. The postcingulum is vestigial: it is present as a very slight and short ridge below the metaconule. The metaconule is large, at least as large as the metacone, and bulbous. It is smaller from M^1^ to M^3^. It is located lingually, i.e., closer to protocone level than to metacone. However, it is not fully lingual in position, i.e., behind the protocone, as in *Eritherium* and other paenungulates. The metaconule is not mesial to the metacone and it is linked to the ectocingulum. There is no indication of a paraconule and there is no posterior conule other than the enlarged metaconule (pseudohypocone). The protofossa is wider than long. The protocone is large and linked to the parastyle by a long and straight preprotocrista. The paracingulum is large. The protocone apex is slightly more distal than the paracone. The postprotocrista is absent. As a result, the protocone and metaconule are widely separated and the protofossa is opened lingually as an incipient but distinct interloph dividing the tooth transversely. The protocone apex is distant relative to the lingual flank of the tooth that is tilted labially.

The molars have three roots. The lingual root is not expanded mesio-distally. The M^3^ has a narrower occlusal outline and a compressed distal part, with reduced metacone, metaconule, and metastylar lobe. Its labial flank is typically oblique transversely, and the parastylar lobe is markedly salient mesio-labially.

*Upper Premolars*: P^4^ is three-rooted and submolariform, with a well developed lingual lobe and protocone. The ectocingulum is reduced, but still distinct on the mesial and distal parts. The paracone is large and bears two longitudinal crests. The parastyle is reduced to absent, and located mesial. The metacone is absent, except for a very small trace of metacone. The protocone is located at the transverse level of the paracone. There is a very small transverse crest on the bottom of the protofossa between the protocone and paracone. The protocone bears a mesial (preprotocrista) and distal (postprotocrista) crest, both of which are linked to a mesial and distal cingulum. Consequently, the protofossa is fully enclosed. There is a small mesial ridge on the mesial flank of the crown, corresponding to a trace of precingulum.

P^3^ is much narrower than P^4^. It consists in one main large cusp, the paracone. This cusp is labio-lingually compressed and has one main crest that is well developed and oblique. By contrast, the mesial flank of the paracone is steep and has not distinct crest. The parastyle is absent. The lingual lobe is small and bears a very small protocone. The labial cingulum is absent, but there is a distinct lingual cingulum. The tooth has two labial roots and one small lingual root.

Upper teeth anterior to P^3^ remain unknown in *Abdounodus hamdii*. The dimensions of the upper teeth of *Abdounodus hamdii* are provided in Table 1.

**Table 1:** Measurements of the upper teeth of *Abdounodus hamdii*, specimen MHNM.KHG.154 (in millimeters; L: length; W: Width; r: right; l: left).

Teeth	Specimen	r-l	r-w	l-l	l-w
M^3^	MHNM.KHG.154	4.2	5.85	4.3	5.9
M^2^	MHNM.KHG.154	5.1	6.4	5	6.3
M^1^	MHNM.KHG.154	4.9	5.7	4.7	5.7
P^4^	MHNM.KHG.154	4.9	6.2	4.65	5.3
P^3^	MHNM.KHG.154	*5.3	*3.7	5	3.9
M^3-1^	MHNM.KHG.154	13.7	-	13.8	-
M^2^-^1^	MHNM.KHG.154	9.5	-	9.5	-
M^3-2^	MHNM.KHG.154	8.8	-	9.1	-
M^3^-P^3^	MHNM.KHG.154	23.8	-	22.4	-
M^3^-P^4^	MHNM.KHG.154	18	-	17.8	-
P^4-3^	MHNM.KHG.154	11.5	-	9.4	-

#### Dentary and lower dentition (Figs 3 and 4)

The new specimen MNHN.F PM92 of *Abdounodus hamdii* provides additional information on the morphology of the dentary, the lower tooth row and the morphology of the tooth roots thanks to a CT scan imaging and 3D digital modelling.

**Fig 3:**
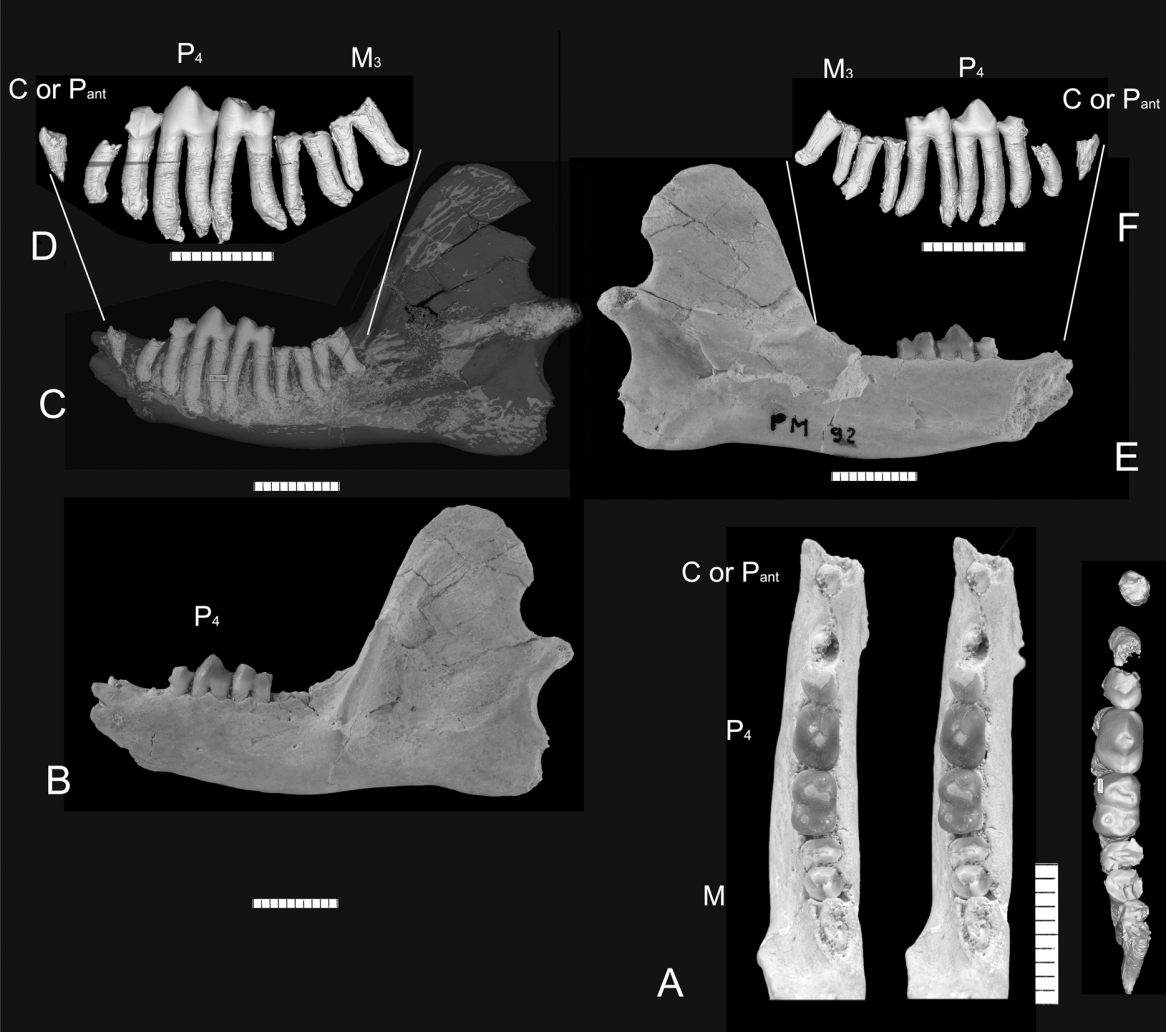
*Abdounodus hamdii*, Selandian (Phosphate level IIa) of the Ouled Abdoun Basin, Morocco. MNHN.F PM92, left dentary bearing roots of M_3_ and M_2_, crown of M_1_, P_4_, P_3_ (damaged) and root of P_2_ or P_1_ or C_1_. (A) stereophotographic pair in occlusal view and 3D model of the isolated teeth in occlusal view reconstructed from the CT scans; (B) Labial view; (C) Transparent 3D model in labial view reconstructed from the CT scans and showing the roots of the teeth; (D) 3D model of the isolated teeth in labial view, reconstructed from CT scans. (E-F) Same in lingual view. Scale bar = 10 mm.

**Fig 4:**
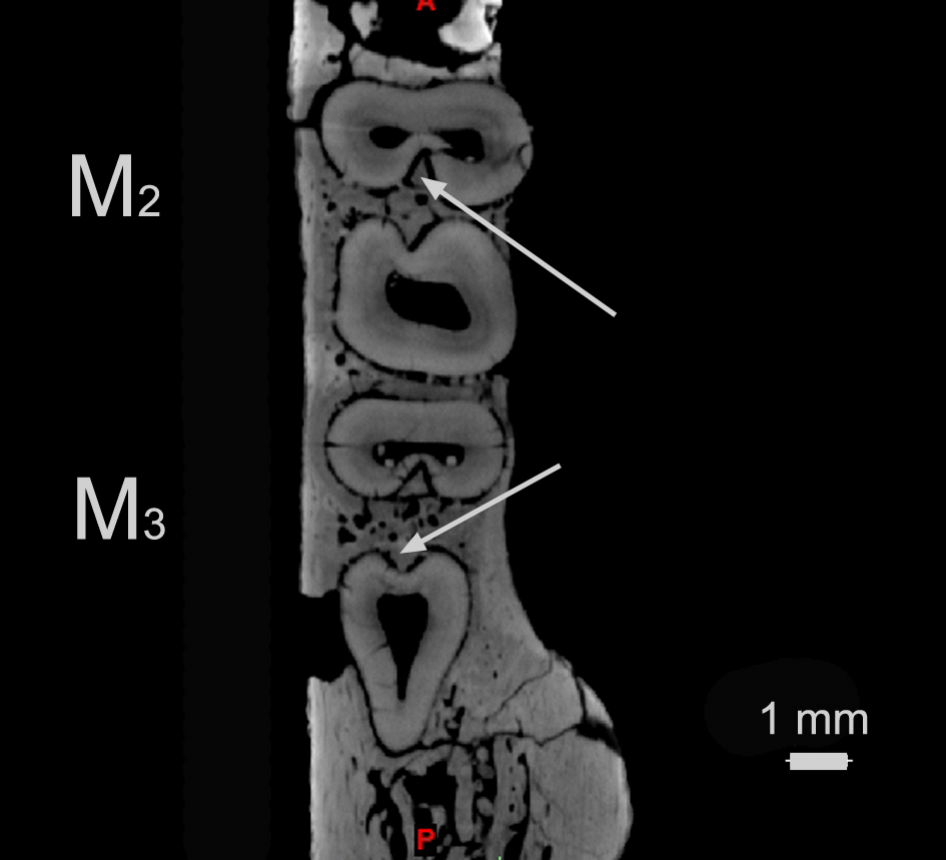
*Abdounodus hamdii*, Selandian (Phosphate level IIa) of the Ouled Abdoun Basin, Morocco. Transverse horizontal CT scan section of the partial lower jaw PM67 preserving M_2_ and M_3_ and showing the vertical root furrow and infilling crest of bone (arrows). A: anterior; P: posterior. Scale bar = 1 mm.

*Dentary (Figs 3 and 4):* The dentary is slender with a narrow corpus (H = 11.5 mm). The corpus is inflated labially, but much less than in *Ocepeia*. It is also convex ventrally. The coronoid process is very high as in *Ocepeia*, but significantly longer. Its anterior margin is slightly inclined posteriorly (angle with alveolar border of about 120°). The masseteric crest is strong and long; it extends high on the lateral surface of the coronoid process as a small crest that curves posteriorly and ends behind its apex; it also extends down low on the corpus, up to a level lateral to M_3_. Consequently, the masseteric fossa is deep and extends very low on the lower jaw. Two small mental foramina occur: the posterior mental foramina is located below P_4_; the anterior one is larger and is located in the symphyseal region below the preserved anteriormost alveolus. The symphysis is short; it ends below the anterior part of P_3_ and is unfused. A small retromolar fossa excavates the anterior side of the coronoid process between the masseteric crest and a medial crest; it is more distinct in old individuals, such as PM68, than in MNHN.F PM92 where the distal part of M_3_ is still partly included in the coronoid process. The coronoid foramen is absent in MNHN.F PM92 (not seen, even in the CT scan slices). The coronoid foramen was also reported absent in *Ocepeia* [21], by contrast to the crown paenungulates, including early genera such as *Seggeurius*, *Eritherium*, *Phosphatherium*, *Arsinoitherium* and *Prorastomus*. The presence of a coronoid foramen (or coronoid canal) was recovered as a paenungulate synapomorphy [18, 21]. *Ocepeia* and *Abdounodus* suggest it is restricted to crown paenungulates.

A small ridge of bone inflated as a medial buttress (but located posterior) extends medially above the mandibular foramen from the posterior part of M_3_ to the articular condyle to which it is linked (Fig 3E). It is more inflated close to the M_3_. The mandibular foramen is large and high dorso-ventrally; it opens in a large groove below the mandibular condyle. It is located below the coronoid process apex. The mandibular condyle lies low above the tooth row (about one tooth height above the tooth row) and is extended transversely. The mandibular condylar process is well individualized by supra- and infracondylar sigmoid notches. The mandibular angular process is moderately developed and only slightly protruding ventro-distally. Although partly broken, it does not extend posteriorly much farther than the mandibular condyle.

The dimensions of the dentary MNHN.F PM92 of *Abdounodus hamdii* are provided in Table 2.

**Table 2:** Measurements of the dentary MNHN.F PM92 of *Abdounodus hamdii* (in millimeters; L: length, H: height, W: width).

MNHN.F PM92	L	W	H
Dentary (max. size)	57.8	8.7	36
Coronoid ap.	18	-	23
Art. cond.	-	11.8	-
Mandibular corpus	-	6	11.5
Tooth row from alveoli, M_3_-I_3_?	28.3	-	-
Tooth row, M_1_-P_4_	9.4	-	-

*Lower tooth row*: The dentary MNHN.F PM92 (Fig 3) preserves four broken roots for both M_3_ and M_2_, the crown of M_1_, P_4_, the posterior part of P_3_, and the root of an undetermined small anterior tooth, either an anterior premolar (P_2_ or P_1_) or the canine (C_1_). At the anteriormost part of MNHN.F PM92, in the symphyseal area, there is a trace of the posterior wall of a deep alveolus for a much larger and slightly proclive root, for an incisor or perhaps a canine. The tooth anterior to P_3_ (premolar or C_1_) is separated from it by a small but distinct diastema. This diastema (L = 2.2 mm) is very similar to that seen in the holotype MNHN.F PM21. It might correspond either to the loss of one or two anterior teeth (anterior premolars or C_1_) or simply to an elongation of the tooth row without any tooth loss. The lower dental row of *Abdounodus* differs from the early paenungulates by the presence of a distinctive lower diastema, whatever may be its anterior dental formula that cannot yet be solved.

*Lower teeth*: MNHN.F PM92 shows that the larger lower teeth are M_1_ and P_4_, i.e., the most central teeth in the tooth row (Fig 3C–3F). One remarkable feature of the lower molars and at least P_4_, which is confirmed by specimen MNHN.F PM92, is that the enamel extends very low on the tooth, in between the two roots below the crown (Fig 3D and [23]). The M_1_ preserved in MNHN.F PM92 does not differ from previously described specimens of *Abdounodus hamdii* [23]. The P_4_ and P_3_ (trigonid) preserved in MNHN.F PM92 are large and simple premolariform and bunodont teeth. Their overall morphology is nearly identical to those of the holotype. The crown of P_4_ is dominated by one main cusp (protoconid) flanked by two main longitudinal crests. This cusp is robust: it is inflated and moderately high (crown less high than long). In occlusal view, the crown is more inflated postero-labially, so that the talonid is slightly wider than the trigonid. There is no trace of metaconid and of labial cingulum. The paraconid is very small and located low, in continuity with a very short and slight mesio-lingual cingulum. The talonid is small and wider than long. It bears three cusps. A small cuspule is inflated at the base of the distal protoconid crest, from which it is separated by a small notch. It corresponds to a protostylid. Two other talonid cusps are distal and aligned transversely. The larger cusp is disto-lingual, and might be the entoconid. The other more labial cusp (hypoconid?) is more or less aligned longitudinally with the protostylid and the distal crest of the protoconid. The postfossid is small but distinct and delimited by the two distal cusps of the talonid. P_3_ is very similar to P_4_. It differs mainly by the narrower occlusal outline and by the smaller talonid cusps.

The 3D reconstruction from CT scan images shows that the roots of lower cheek teeth are strong and very long (Fig 3C–3F), with maximal length for M_1_ and P_4_. The roots are recurved posteriorly and inflated at their apex. They are more divergent on the posterior teeth (M_3-2_) than on the anterior ones (M_1_-P_4-3_). The posterior root of M_3_ is inclined posteriorly and noticeably elongated, even more than the talonid. The roots of the molars show an interradicular groove that fits with a vertical crest of the interradicular bone (Fig 4).

The dimensions of the lower teeth preserved in specimen MNHN PM92 of *Abdounodus hamdii* are provided in Table 3. It should be noted that lower molar proportions of *Abdounodus* seem to follow the Inhibitory Cascade (IC) model of Kavanagh et al. [26], (1) with a ratio of M/2 size to that of M/1-3 close to the predicted value of one third (0.34 in specimen OCP DEK/GE 310 [23]), and (2) with a ratio of M/2 and M/3 to M/1 higher than 1. It corresponds to a larger size of the posterior molars in *Abdounodus* and a low inhibition of M/2-3 by M/1 in the IC model (weak inhibitory cascade in *Abdounodus* [26–28]). The molar size proportions of *Abdounodus* are consistent with an herbivorous mammal [26–27].

**Table 3:** Measurements of the lower teeth of *Abdounodus hamdii*, specimen MNHN.F PM92 (in millimeters).

Teeth	Specimen	l	w	H
M_3_	MNHN.F PM92	*4.1 (r)	*2.1 (r)	-
M_2_	MNHN.F PM92	*4.9 (r)	* 3.2 (r)	-
M_1_	MNHN.F PM92	4.8	3.3	*3.6
P_4_	MNHN.F PM92	5	3.5	4.6
P_3_	MNHN.F PM92	*6.1 (r)	2.7	-
Diastema P_3_ to P_2_, P_1_ or C_1_	MNHN.F PM92	2.2	-	-
P_2_, P_1_ or C_1_	MNHN.F PM92	*2.1 (r)	*2.1 (r)	-

(*) estimations

(r) = after roots).

#### Dental formula of *Abdounodus hamdii*

It is difficult to determine the anterior dental formula in *Abdounodus*, and especially to identify what tooth loss—if any—corresponds to the diastema seen in the lower jaw. Since basal and early taxa such *as Eritherium* and *Seggeurius*, as well as *Arsinoitherium*, suggest an ancestral paenungulate morphotype retaining the full eutherian dental formula, any tooth loss in *Abdounodus* would be a remarkable precocious derived trait, as seen in *Ocepeia* that has lost the two first premolars [21]. For the moment, this cannot be checked yet, and the dental formula of *Abdounodus hamdii*, as presently documented, is identified as I^?^/_?_, C^?^/_?,_ P^?-?-3-4^/_?-?-3-4_, M^1-2-3^/_1-2-3_. That is, all teeth of *Abdounodus* anterior to the third premolar are coded unknown and/or unidentified in our phylogenetic analysis.

### Occlusion and wear pattern of *Abdounodus hamdii* (Figs 5 and 6)

**Fig 5:**
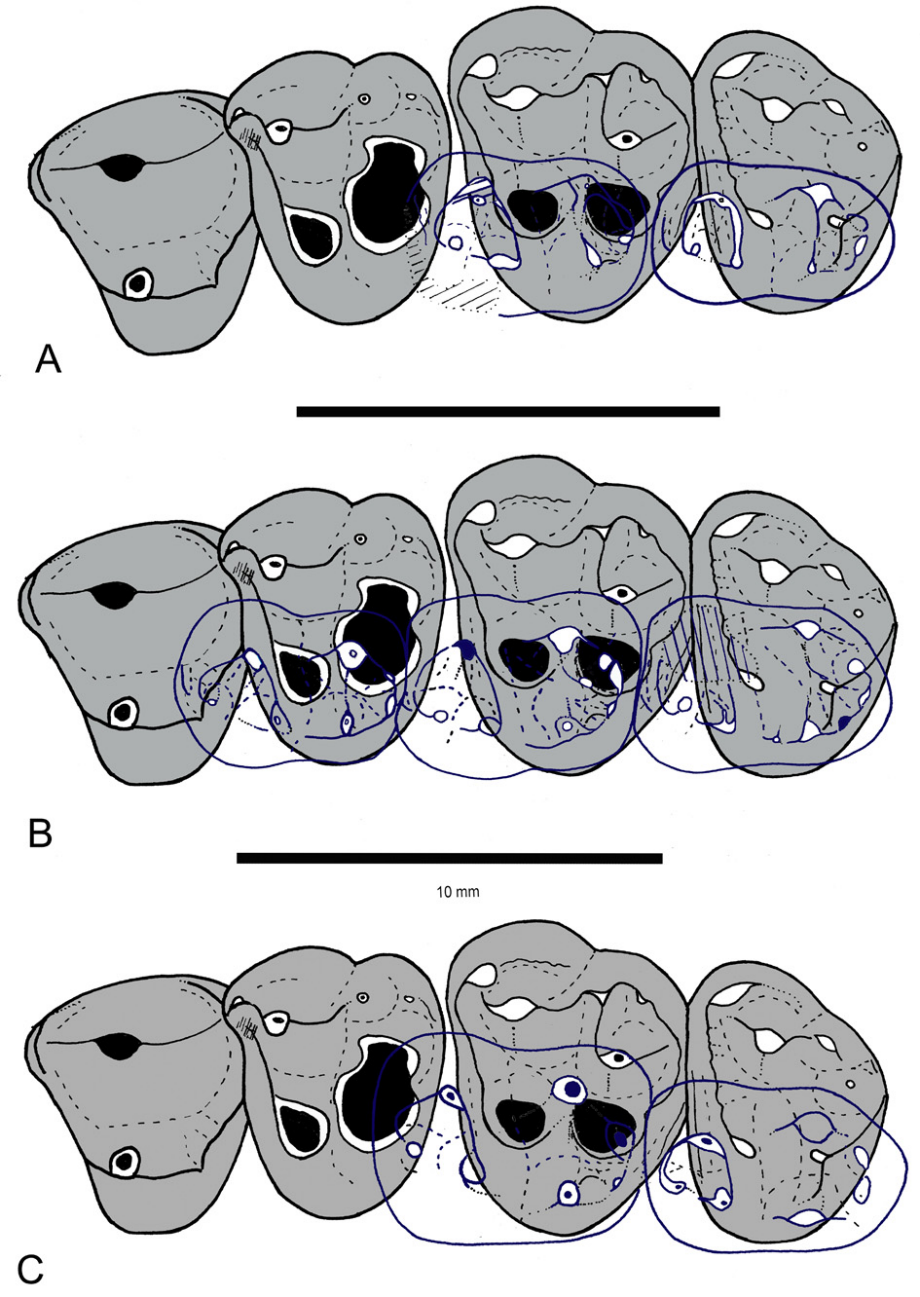
Occlusal sketch of the molars of *Abdounodus hamdii* reconstructed with the maxillary dentition of specimen MHNM.KHG.154. (A) occlusal sketch of MHNM.KHG.154 with M_2-3_ of specimen PM68; (B) cclusal sketch of MHNM.KHG.154 with M_1_, M_2_, M_3_ of specimen OCP DEK/GE 310; (C) occlusal sketch of MHNM.KHG.154 with M_2-3_ of specimen PM67. The occlusion of opposed molars is reconstructed here in sub-centric position.

The pattern of the occlusion of the upper molars of specimen MHNM.KHG.154 with described lower molars of *Abdounodus hamdii* [22–23] fits well both in size and morphology as illustrated in Fig 5. There is a slight variation of size in known specimens of the lower dentition, so that some specimens such as OCP DEK/GE 310 fit less well with MHNM.KHG.154 than others (PM68, PM67, MNHN.F PM35). However, this variation is weak (10–20% in size) and it is clearly linked to individual differences. The good occlusal fitting of upper molars MHNM.KHG.154 and known lower molars of *Abdounodus hamdii* is supported by a study of their occlusion with 3D virtual simulation with the help of the Occlusal Fingerprint Analyser software (OFA: [29,30]), as well as by the wear pattern. This clearly supports the conspecific identity of MHNM.KHG.154 and the lower molars of *Abdounodus hamdii*, in addition to several shared morphological traits such as, in particular, the strong bunodonty and the incipient bilophodont morphology.

**Fig 6:**
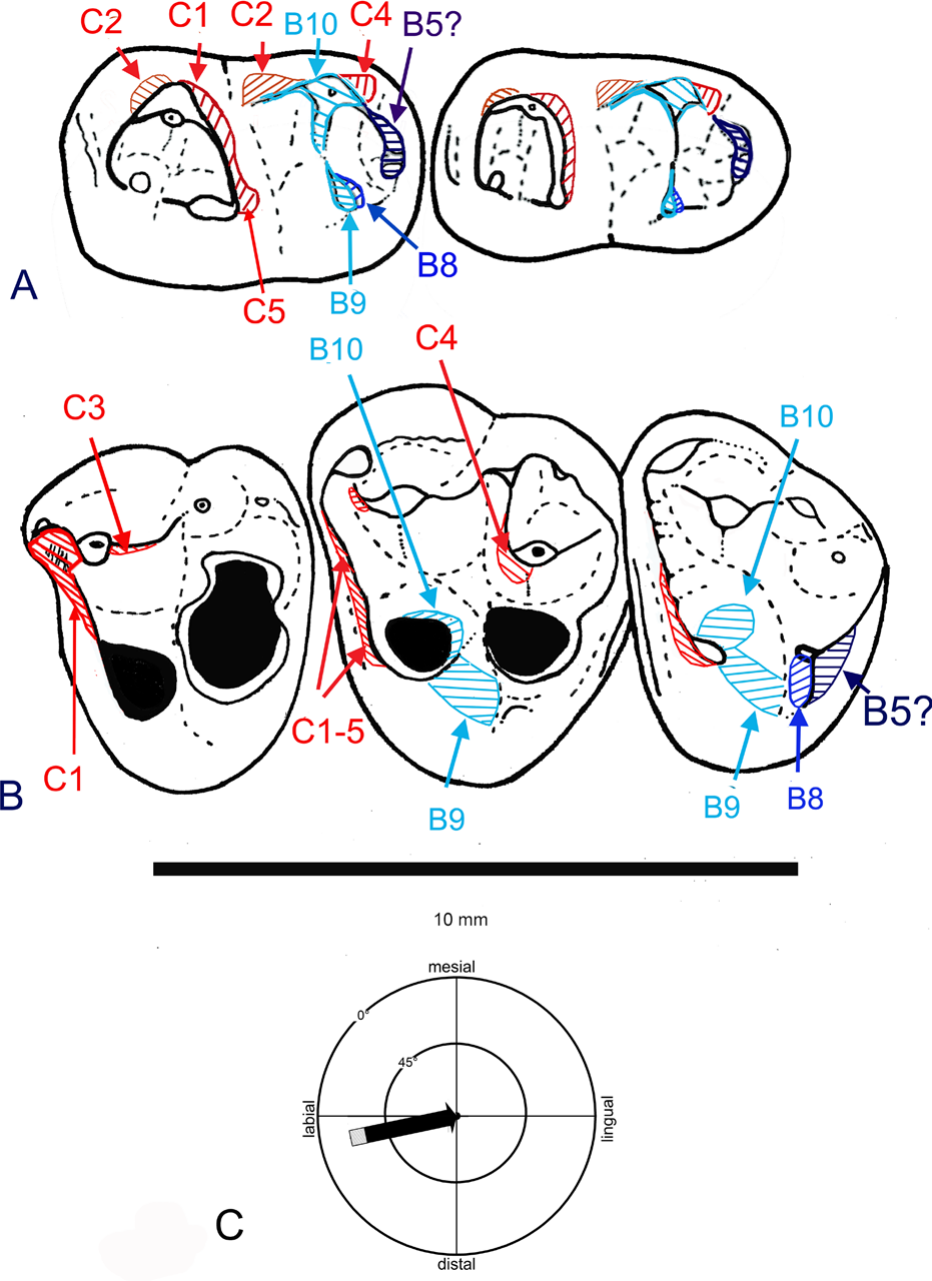
Wear facets of upper (MHNM.KHG.154) and lower molars of *Abdounodus hamdii*, and mastication compass following Koenigswald *et al*. **[30]**. (A) Right lower molars M_2_ and M_3_; (B) left upper molars M^1^, M^2^, M^3^. C1-C10: Crompton [31] wear facets of tribosphenic molars (in red); B1-10, Butler [32] wear facets of lophodont molars (in blue). (C) Mastication compass of *Abdounodus hamdii*, indicating the inclination and direction of the lower jaw motion during power stroke of mastication. Note that the lower jaw motion is mostly labio-lingual (transverse) and horizontal, and is restricted to phase I of mastication.

#### Attrition wear pattern

The detailed study of the pattern of occlusion, with help of occlusal sketches and OFA 3D virtual simulation, shows several occlusal phase I shearing contacts between opposite molars during the power stroke of mastication. They are summarized in Table 4 and Fig 6.

**Table 4:** Occlusal shearing contacts between the opposite molars of *Abdounodus hamdii* during the power stroke of mastication as identified from occlusal sketches (Fig 5) and from 3D simulation of occlusion with the OFA software.

Shearing occlusal contacts	Upper molars (MHNM.KHG.154)	Lower molars
1	Anterior side of paracone (preparacrista) and protocone (preprotocrista)	Posterior side of protoconid and metaconid (protocristid)
2	Posterior side of metacone (postmetacrista) of opposite anterior upper molar	Anterior side of protoconid (paracristid, labial part) of opposite posterior lower molar
3	Posterior side of paracone (postparacrista)	Mesial side of cristid obliqua
4	Anterior side of metacone (premetacrista)	Posterior side of hypoconid (postcristid)
5	Lingual side of protocone	Anterior side of entoconid and hypolophid (lingual segment)
6	Posterior side of protocone	Posterior side of entoconid and hypolophid (lingual segment)
7	Anterior side of metaconule (pseudohypocone)	Posterior side of entoconid and hypolophid (lingual segment)
8	Posterior side of metaconule (postmetaconule crista)	Mesial side of postentoconulid and hypoconulid (postcristid)

The shearing contacts 5–7 evidence a remarkable feature of *Abdounodus hamdii* occlusal pattern: the occlusion of the hypolophid and linked cusps with the interloph of upper molars. This is well seen in the occlusal sketch of upper and lower molars (Fig 5). The occluding morphological structures—interloph and lophs (including metaloph)—are still poorly developed in *Abdounodus*, but their occlusal relations are well characterized, including in wear pattern (Fig 6, Table 5). Their functional patterning was indeed significant.

**Table 5:** Wear pattern of the upper and lower molars of *Abdounodus hamdii*: attritional wear facets and related occluding structures. C1-6 primitive shearing facets of tribosphenic molars following the nomenclature of Crompton [31], B1-B10 shearing facets of lophodont molars described by Butler [32].

Attritional wear facets (all phase I)		Shearing occlusal structures (Table 4)	Specimen		Comments
Tribosphenic molars (Crompton 1971)	Lophodont molars (Butler 1952)		upper molars	lower molars	
C1	B2	1: Preparacrista-preprotocrista / protocristid	MHNM.KHG.154	Holotype, OCP DEK/GE 308, MNHN PM35, OCP DEK/GE 310, PM67, PM68, MNHN PM92	Predominant attritional wear and function in *Abdounodus*
C2	B1	2: Postmetacrista / paracristid	Absent or weak	Weak, except MNHN PM35, PM67	Reduced attritional wear and function in *Abdounodus*
C3	B6	3: Postparacrista / cristid obliqua	Weak	Weak: PM68	Reduced attritional wear and function in *Abdounodus*
C4	B7	4: Premetacrista / Hypoconid-postcristid	MHNM.KHG.154	Holotype, OCP DEK/GE 308, MNHN PM35, OCP DEK/GE 310, PM67, PM68	C4 > C3 and extended lingually to metacone—possibly related to metaloph development
C5	B3	Preprotocrista-protocone mesial / protocristid-metaconid distal	MHNM.KHG.154	PM67, PM68,? MNHN PM92	In continuity with C1; protoloph / protolophid shearing pair
(modified C6^1^)	B9	6: Protocone distal / entoconid mesial-hypolophid	MHNM.KHG.154	OCP DEK/GE 308, MNHN PM35, PM67	Interloph / hypolophid occlusion
(modified C9? ^2^)	B10	5: Protocone lingual / hypoconid apex-hypolophid	MHNM.KHG.154	Holotype, OCP DEK/GE 308, MNHN PM35, OCP DEK/GE 310, PM67, PM68	Interloph / hypolophid occlusion
-	B8	7: Metaconule mesial / entoconid distal	MHNM.KHG.154	PM68 (M_3_), MNHN PM92	Metaconule-Interloph / hypolophid occlusion (= posterior loph shearing)
-	B5?	8: Metaconule distal / postentoconulid-hypoconulid mesial	MHNM.KHG.154 (M^3^)	OCP DEK/GE 308, MNHN PM35, OCP DEK/GE 310, PM67, PM68, MNHN PM92	Homology with facet B5 of Butler (1952)?

^(1)^ resulting from the regression of the postprotocrista and the development of the hypolophid by comparison to the tribosphenic pattern

(2) possible modification the grinding occlusal contact 9 (facet C9) of the tribosphenic molars for shearing with the hypolophid (see text)

Several attrition wear facets with a variable extension are visible (Table 5, Fig 6) in the known material of *Abdounodus hamdi;* all correspond to phase I shearing occluding contacts of opposite molars. These facets include the classical shearing facets C1-6 known in tribosphenic-tritubercular molars that were numbered by Crompton [31], and some additional facets (B8-10) known in quadritubercular and lophodont molars that were numbered by Butler [32]: see Table 5.

Some of these wear facets are predominant and easily visible. In general, the anterior upper wear facets and the posterior lower wear facets are the best developed. The most important wear facets and shearing function of the molars of *Abdounodus hamdii* correspond indeed to the prevallum-postvallid shearing between both preparacrista and preprotocrista and the protocristid (Table 4: 1). It is illustrated by the wear facets C1 and C5. In *Abdounodus*, these two wear facets are difficult to distinguish from each other because of their continuity. In upper molars they are present on the preparacrista in most worn teeth (M^1^) and on the preprotocrista. In the lower molars, they are widely developed on the protocristid below the protoconid and metaconid with labially oblique wear striae; in some specimens (PM67) C1 extends in the hypoflexid. The facet C1 also has a characteristic semi-lunar shape in some specimens (OCP DEK/GE 310).

Other occlusal shearing contacts of phase I are marked by more or less distinct wear facets on both upper and lower molars (Table 5, Fig 6). Facet C2 (postmetacrista/paracristid) is weak and seen mostly in PM67. Facet C4 (premetacrista/postcristid) is better developed than facet C3 (postparacrista/cristid obliqua), again illustrating the predominance of the anterior/posterior shearing between upper and lower molars. The mesial side of entoconid and lingual segment of hypolophid shows a wear facet (Fig 6) that corresponds to shearing with protocone and to a wear facet on its distal flank (Table 4: 6); this is wear facet B9 of lophodont molars and probably the modified wear facet C6 of tribosphenic-tritubercular molars (Table 5). There is a wear facet on the hypoconid and hypolophid (labial segment) (Table 5, Fig 6) that corresponds to the shearing with the protocone lingual flank. It corresponds to the facet B10 of bilophodont molars. By its topographical position, this facet might be homologous to the grinding wear facet C9 resulting from the protocone/hypoconid contact of primitive tribosphenic molars, but as a modification for a new shearing function of the protocone with the neomorphic hypolophid. Consistently, this facet B10 is present on the internal flank of hypoconid in some specimens (OCP DEK/GE 308, MNHN.F PM35, PM67) where the hypolophid is poorly differentiated, being represented by the convex internal flank of hypoconid instead a distinct crest. The development of the hypolophid is indeed variable in *Abdounodus*. There is a variable facet B8, known in lophodont molars, on the posterior flank of entoconid and lingual segment of hypolophid that corresponds to shearing with the postentoconule and to a wear facet on its mesial side (Table 4: 7). There is a well distinct wear facet on the hypoconulid-postentoconulid apex that corresponds to shearing with the metaconule-postmetaconule crista (Table 4: 8) and to a wear facet on its distal flank (best seen on M^3^, less abraded). This facet is unknown in tribosphenic molars, but it might correspond to the facet B5 described in lophodont molars of perissodactyls by Butler [32] and Hooker [33]. In the perissodactyls, the facet B5 extends mostly on the disto-lingual side of the hypocone and on the cingular-like hypoconulid (i.e., forming the postcingulid), as well on the disto-lingual side of the protoconid and paracristid. In *Abdounodus* there is no trace of wear on the lingual side of the paracristid and the facet extends distally on the metaconule (Table 5) rather than on a hypocone. The homology for *Abdounodus* with the facet B5 of perissodactyls remains indeed uncertain; alternatively, the wear facet of *Abdounodus* could be new (i.e., not B5), having no equivalent in Perissodactyla.

*Abdounodus* shows indeed some wear facets known in lophodont molars, which are unknown in tribosphenic-tritubercular molars (B8, B5?) or which have been noticeably modified (B10, B9). With respect to the tribosphenic-tritubercular plan, they are generated by new occluding structures: the hypolophid and opposed interloph-metaconule (pseudohypocone). The facet B8 (as well as the transverse extension of C4) is in particular noticeably linked to the shearing of the distal loph of bilophodont mammals.

Wear striae are seen on several attrition wear facets in the teeth of *Abdounodus hamdii*, but especially on the facet C1 where they are inclined labially in lower molars. They are moderately oblique relative to the horizontal axis with an angle varying from 30° to 40° (Table 6), corresponding to a shallow to steep inclination according to the definitions of Koenigswald *et al*. [30]. They indicate a dominant transverse component of the motion of the lower teeth during phase I of power stroke of mastication.

**Table 6:** Angle of the wear striae of the upper and lower molars of *Abdounodus hamdii* with respect to the horizontal plan (= relative inclination of the motion of the lower jaw during phase I); wear facets number of Crompton [31].

Specimen	Wear facet	Wear striae inclination vs hor. axis
MNHN.F PM35, M_2_	4	40°
PM68, M_2_	1	30°
PM67, M_2_	1	35°
MHNM.KHG.154, LM^1^	1	30°
MHNM.KHG.154, RM^1^	1	35°

There is no distinct phase II grinding wear facet in *Abdounodus hamdii* upper and lower molars.

#### Abrasion wear pattern

Abrasion is extensive in both upper molars of MHNM.KHG.154 and lower molars of *Abdounodus*, with a very similar pattern. The most worn cusps are labial in lower molars and lingual in upper molars, consistently with relative occlusal motion of opposite teeth. The abrasion excavates the cusps deeply in the dentine as seen especially on the M^1^ of specimen MHNM.KHG.154 (Figs 1 and 2). One peculiar feature of the abrasion seen in the teeth of *Abdounodus* is that it extends transversely: from protoconid to metaconid, i.e., the protolophid, on lower molars (holotype, MNHN.F PM35, MNHN.F PM92; Fig 3A), and from metaconule to metacone, i.e., the metaloph of upper molars (Figs 1B and 6). This abrasion pattern indicates a significant transverse motion of the lower molars in food-tooth contacts during preparatory stroke. This is consistent with the lateral inclination of the wear striae seen on wear facets and with the pattern of occlusion (see above, and Fig 6C). The abrasion suggests indeed that the lophs were even functional during preparatory stroke in *Abdounodus*.

The abrasion pattern of *Abdounodus hamdii* highlights 1) a strong vertical component and crushing dental function, and 2) functional lophs and some transverse movement of lower teeth during preparatory stroke of mastication.

#### Main features of the mastication and diet of *Abdounodus hamdii*

The mastication process during the power stroke in *Abdounodus hamdii* is predominated by the phase I shearing function; shearing was enhanced in *Abdounodus* with the evolution of new occluding structures (and related wear) such as the hypolophid of lower molars and the interloph and metaconule-pseudohypocone (metaloph) of upper molars. The phase II is absent as illustrated by the absence of grinding wear facet, and by the occlusal sketches and the 3D virtual simulation with the OFA software of the occlusion of opposite teeth (Fig 6C). The phase I was characterized by a significant horizontal component of lower jaw motion (Fig 6C). This is unexpectedly also shown by the abrasion pattern that marks the transverse lophs. The extensive abrasion indicates a strong vertical and crushing dental function during preparatory stroke in *Abdounodus*, and a hard and abrasive plant items diet. This is in agreement with the bunodont morphology of the teeth and with their very long and robust roots.

### Comparisons of the new material of *Abdounodus hamdii*

Comparisons show closest dental resemblances of *Abdounodus* with some of the earliest afrotherian paenungulates; some shared traits are also seen with the enigmatic extinct family Ptolemaiidae that is considered by some authors as a possible early relative of the Tubulidentata [34].

#### *Ocepeia* (Afrotheria, Paenungulata?)

**Fig 7:**
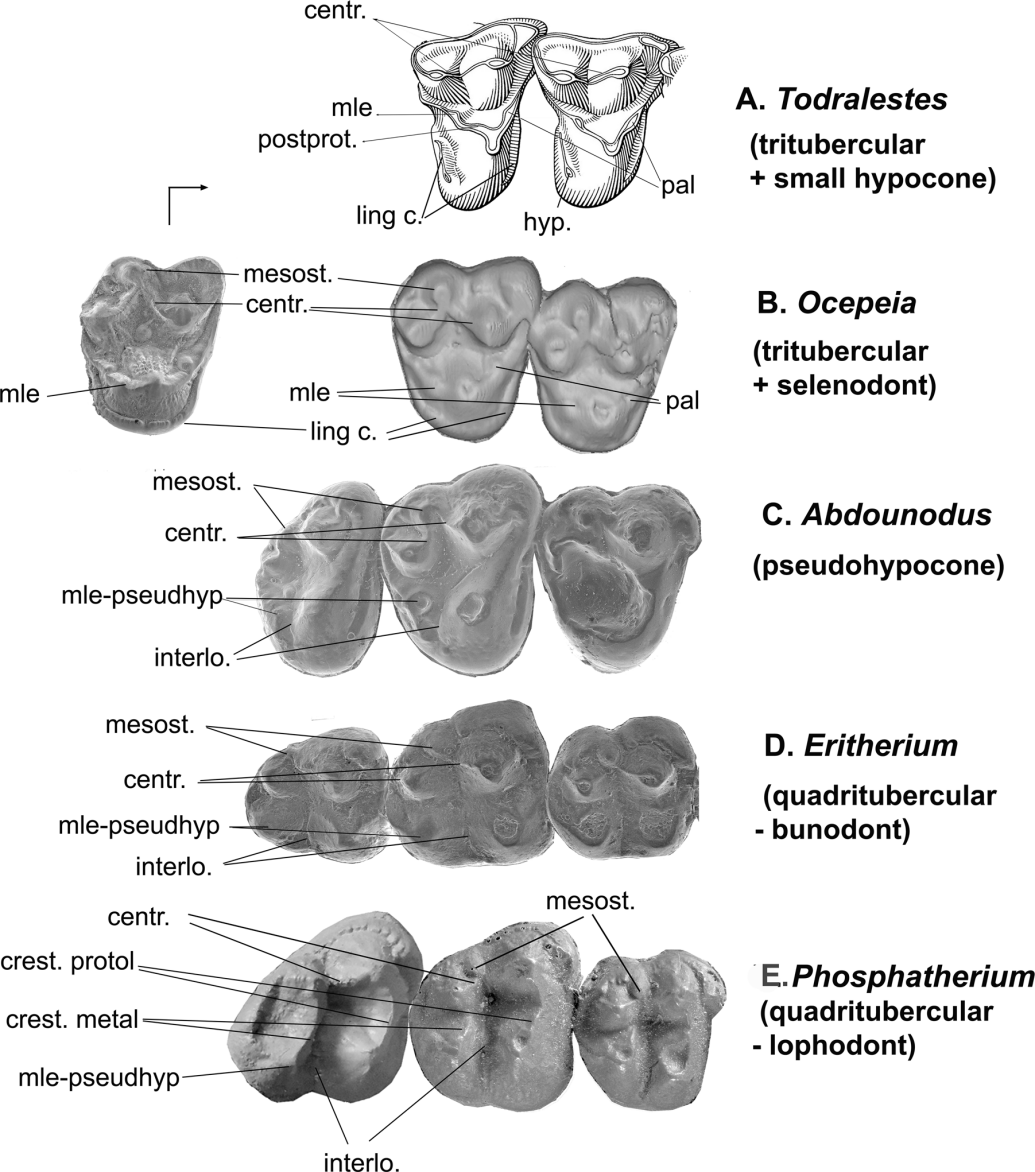
Comparison of the upper molar pattern (occlusal view) of early paenungulatomorphans (Afrotheria) with respect to the tribosphenic-tritubercular pattern of *Todralestes*, with indication of significant homologous structures. (A) *Todralestes variabilis* (Eutheria, Pantolesta?), M^2-1^; (B) *Ocepeia grandis* (left), M^3^, and *O*. *daouiensis* (right), M^2-1^ (Paenungulatomorpha); (C) *Abdounodus hamdii* (Paenungulatomorpha), M^3-1^; (D) *Eritherium azzouzorum* (Paenungulata, Proboscidea), M^3-1^; (E) *Phosphatherium escuilliei* (Paenungulata, Proboscidea), M^3-1^. Not to scale. All teeth figured as right teeth. Abbreviations: centr: centrocrista; hyp: hypocone; interl: interloph; ling c: lingual cinglum (pre- and postcingulum); crest metal and crest protol: full crest-like protoloph and metaloph (true lophodonty); mle: metaconule; mesost: mesostyle; pal: paraconule; pseudohyp: pseudohypocone; postprot: postprotocrista.

*Ocepeia daouiensis* was found in the same Selandian level from the Ouled Abdoun phosphate Basin (especially Sidi Chennane quarries [24]) of Morocco that has yielded *Abdounodus* and *Eritherium* [21–23]. A second species, *O*.*grandis*, was recently described [21] from an upper Paleocene level in the Ouled Abdoun phosphate Basin that is dated as Thanetian. *Ocepeia* was considered as a possible stem relative of the Paenungulata [21], although this was not supported by the cladistic analysis. Some conspicuous resemblances can be noticed between the upper molars of *Abdounodus* and *Ocepeia*, such as the occlusal outline, the ectoloph selenodont and linked to inflated styles (especially the mesostyle), the metaconule shifted lingually close to protocone, the reduced lingual cingulum and absence of hypocone (Fig 7).

However, *Abdounodus* differs from *Ocepeia* by many features. The molars of *Abdounodus* are much more bunodont than those of *Ocepeia*: the crests are less developed and the cusps are more bulbous. These derived traits of *Abdounodus* are closer to *Eritherium*. The occlusal outline is narrower, but broader narrow lingually. The stylar shelf is narrower. Consequently, the mesostyle is closer to the paracone and metacone, and the paracrista and metacrista are shorter transversely. The parastylar lobe is less labially expanded. The mesostyle and parastyle are proportionally smaller. The mesostyle is more distal and more aligned transversely with the metacone, as in *Eritherium*.

By comparison to *Eritherium*, the lingual cingulum, especially the postcingulum, is more reduced and remains in the form of mere traces. The metaconule is more shifted lingually relative to the metacone. The postmetaconule crista joins labially the ectocingulum with a distinct metacingulum, in contrast to *Ocepeia* in which it ends at the lingual flank of the metacone. There is no trace of a paraconule. The metaconule is much larger and bulbous. The postprotocrista is completely absent and the protocone and metaconule are widely separated. Consequently, the protofossa is opened lingually by an incipient interloph. The protocone is less long and voluminous. The protocone apex is more anterior, more aligned transversely with the paracone. The M^3^ of *Abdounodus* is smaller than the M^2^; this is distinctive from *Ocepeia* in which M^2^ and M^3^ are similar in size. The premolars of *Abdounodus* are narrower, with a less developed lingual lobe and protocone and a transversely more compressed paracone.

#### *Eritherium* (Afrotheria, Paenungulata, Proboscidea)

*Eritherium* comes from the same Selandian level in the Ouled Abdoun basin (Morocco) than *Abdounodus* and *Ocepeia*, and is the most primitive known proboscidean [19–20]. *Abdounodus* intriguingly resembles *Eritherium* in several traits (Fig 7). The incipient quadritubercular morphology of upper molars of *Abdounodus* with an inflated metaconule-pseudohypocone (see below) is strikingly reminiscent of *Eritherium*. *Abdounodus* and *Eritherium* also share the strongly bunodont molars, a mesostyle distally close to the metacone, a protocone in anterior position (aligned transversely with paracone), the presence of the interloph, and the absence of paraconule. They also have similar wear pattern with strong development of abrasion, especially on lingual cusps of upper molars. As a whole, the bilophodont pattern is much better characterized in *Eritherium* (although still very primitive) as illustrated by the transverse alignment of labial and lingual cusps that have similar large size. In *Abdounodus*, the metaconule is less enlarged and closer to the paracone: the pseudohypocone is not as fully developed as in *Eritherium* where it has a similar or even larger size with respect to the protocone. Consequently, the molar occlusal outline remains triangular in *Abdounodus* in contrast to the quadrangular outline in *Eritherium* (Fig 7). In *Eritherium*, the centrocrista is nearly rectodont (more rectilinear longitudinally), and the mesostyle is closer to it. The mesostyle and parastyle are larger, and the paracrista and metacrista are shorter in *Abdounodus*. The M^3^ is smaller relative to M^2^. The interloph is less developed (narrower). The lingual root is not enlarged. The upper premolars, especially P^3^, are narrower in *Abdounodus*.

#### *Seggeurius* (Afrotheria, Paenungulata, Hyracoidea)

The basalmost hyracoid *Seggeurius* known from the Early Eocene Algerian site of El Kohol [35–37] and probably also from the Ouled Abdoun basin [38], shares some features with *Abdounodus* and *Eritherium* such as the selenodont ectoloph linked to inflated styles, the reduced lingual cingulum (esp. postcingulum) and the absence of small labial conules. Its upper molars differ mainly in its more advanced lophodonty, with more developed and crest-like lophs, the large M^3^, the stronger styles and more developed selenodont ectoloph. The upper premolars of *Seggeurius* have a more developed metacone, and the P^3^ is more extended transversely, with a larger protocone.

#### Ptolemaiidae (Afrotheria?)

Ptolemaiids are endemic African placental mammals known from the early Oligocene (Fayum) and early Miocene and of uncertain relationships; they were recently considered as related to afrotherians, and especially to tubulidentates [34,39,40]. Ptolemaiids share with *Abdounodus* the strong roots, the bunodont molars, the inflated paraconid, a mesio-distally compressed trigonid, a reduced lingual cingulum in upper molars, and a small third molar. Among ptolemaiids, *Ptolemaia* from the early Oligocene of the Fayum (Egypt) more closely recalls *Abdounodus*, for instance in the anterior diastema seen in *P*. *grangeri*. However, *Abdounodus* differs from all ptolemaiids in many ways. The crown of the lower molars of *Abdounodus* is more inflated, and lower and much less hypsodont than in ptolemaiids, especially below the talonid. The upper molars of *Abdounodus* lack labial conules. They are narrower transversely, and differ also by the selenodont ectoloph (rectodont in ptolemaiids), and the styles that are present and inflated. Many other differences with *Ptolemaia* are seen in the lower dentition of *Abdounodus* (e.g., hypolophid present, less shortened talonid, unfused roots of M^3^, M^3^ much less reduced, premolars not enlarged).

## Molar pattern of *Abdounodus* and its significance (Figs 7 and 8)

The upper dentition of *Abdounodus* shows morphological affinities with early and basalmost paenungulates such as *Eritherium* and *Seggeurius*, as well with the afrotherian *Ocepeia* (Fig 7). We discuss below the significance of the dental morphology of *Abdounodus*, in light of the new material reported here.

**Fig 8:**
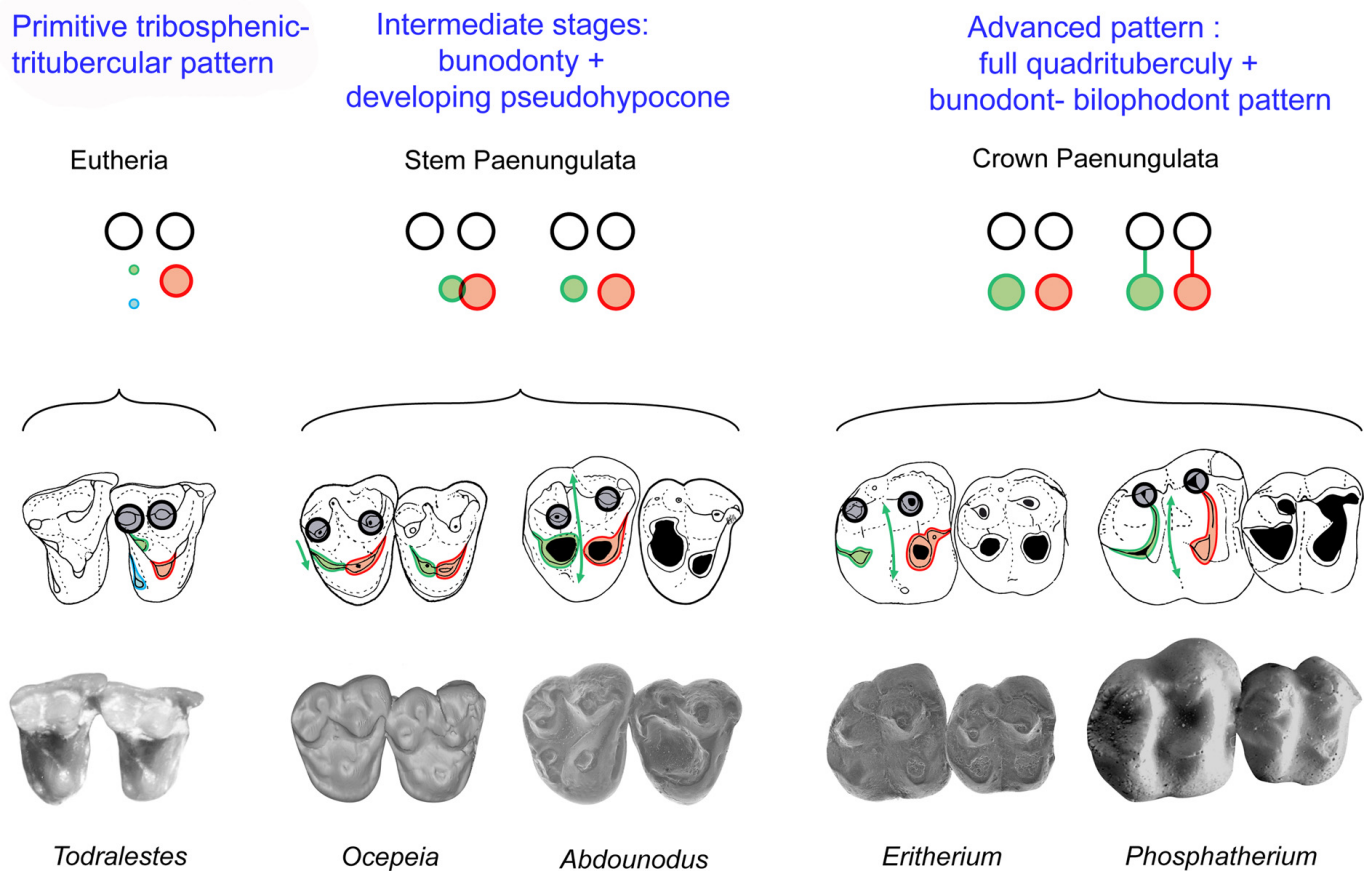
Origin and evolution of the bilophodont pattern in paenungulates: a new structural scenario for upper molars including the stem taxa *Ocepeia* and *Abdounodus* which documents two intermediate stages between the tribosphenic-tritubercular pattern and the quadritubercular-bilophodont pattern. Photography and occlusal sketch of teeth, all figured as right teeth. Symbols of dental structures: Black circles: paracone and metacone, green circle: metaconule; red circle: protocone; blue circle: hypocone; transverse lines: proto- and metaloph; double arrow: interloph.

### Metaconule, pseudohypocone, and quadrituberculy

The postero-lingual cusp of the upper molars preserved in MHNM.KHG.154 is identified here as an enlarged and lingually shifted metaconule, i.e., as a pseudohypocone, rather than as a true hypocone derived from the postcingulum (cingular hypocone). This is primarily indicated by the topographical relations and relative size of the cusp. In *Abdounodus*, it still remains in an intermediate structural stage between the primitive tribosphenic-tritubercular pattern (e.g., *Todralestes*), where the metaconule is small and labial, and the quadritubercular and bunodont-bilophodont pattern (e.g., *Eritherium*, *Seggeurius*), where it is as large and lingual than the protocone and forms a metaloph by its transverse alignment with the metacone, as seen with the hypocone (hence the term “pseudohypocone”). The intermediate structural stage seen in *Abdounodus* is derived in the lingual position of the metaconule close to the protocone, and in its bulbous shape and enlarged size (at least as large as the metacone).

Other morphological features that support development of a metaconule-derived pseudohypocone in *Abdounodus* are as follows:

1. presence of a vestigial postcingulum below the metaconule and concomitant absence of a true cingular hypocone;
2. absence of other conule than the metaconule-pseudohypocone;
3. metaconule still linked to postmetaconule crista, but independent from the more posterior and vestigial postcingulum.

In addition, it is noteworthy that early and basal paenungulates generally lack conules, although a small and possible secondary “paraconular swelling” is known in sirenians and in some early hyracoids. Conules are absent for instance in *Eritherium*, *Phosphatherium*, *Numidotherium*, *Seggeurius*, *Dimaitherium*, *Namatherium*, and *Palaeoamasia*. This is consistent with paraconule reduction and metaconule transformation into a pseudohypocone in stem paenungulates such as *Abdounodus*.

The comparison of *Abdounodus* with *Ocepeia* (Fig 7) highlights the homology of the metaconule in the two taxa. In *Ocepeia*, the metaconule is unambiguously identified: it is still linked to the postprotocrista and independent from the lingual cingulum (postcingulum) which is small but distinct (Fig 7 and [21]). In *Abdounodus*, the cusp has an identical topographical position as in *Ocepeia*. In both taxa, the metaconule is characterized by its lingual location, close to the protocone and related shortened postprotocrista, in contrast to the generalized tribosphenic plan. By comparison to tribosphenic eutherians such as *Todralestes*, *Ocepeia* also shares with *Abdounodus* the trend toward an enlargement of the metaconule. However, *Ocepeia* lacks a pseudohypocone: the metaconule remains poorly inflated and it is still linked to the protocone by a distinct postprotocrista. *Abdounodus* is more advanced in the large, bulbous and fully separated metaconule; this pattern is closer to the quadritubercular and bunodont-bilophodont molar morphology of the paenungulates (Fig 8). It indeed already possesses a pseudohypocone in contrast to *Ocepeia*, which basically retains the tritubercular pattern of the tribosphenic mammals in this respect. With respect to *Abdounodus*, *Ocepeia* therefore displays an additional more primitive intermediate structural stage between the tribosphenic-tritubercular plan seen in *Todralestes* and the quadritubercular bunodont-bilophodont pattern of *Abdounodus*, *Eritherium*, and *Seggeurius* (Fig 8). *Abdounodus*, alongside with *Ocepeia*, further evidences and documents the morphoclinal development of a pseudohypocone from a metaconule in paenungulates. Moreover, *Ocepeia* retains a small lingual cingulum that does not show any trace of hypocone, thereby confirming that the morphotypic ancestral molar pattern of paenungulates lacks a hypocone.

Comparison of *Ocepeia* further adds evidence for evolution and presence of a metaconule-pseudohypocone in *Abdounodus*. *Ocepeia*, *Abdounodus*, *Eritherium*, and *Phosphatherium* illustrate a remarkable structural morphocline showing the initial evolution of the quadrituberculy and bilophodonty from the primitive tribosphenic-tritubercular pattern such as that of *Todralestes* (Fig 8). In this morphocline, *Ocepeia* and *Abdounodus* display the very first steps of the emergence of the pseudohypocone, a key apomorphic feature of the quadritubercular and bunodont-lophodont pattern shared by crown paenungulates.

### Incipient bunodont-bilophodont pattern

Several molar traits indicate an early bunodont-lophodont patterning in *Abdounodus*. The relative position of the mesostyle and metaconule that are more or less transversely aligned with the metacone and its premetacrista corresponds to the basal arrangement of the metaloph. This results especially from the distal shift of the mesostyle in front of the metacone and from the transverse orientation of the premetacrista, in line with the metaloph. This is the pattern seen in basal crown paenungulates such as especially the palaeoamasiid embrithopods. The specialized and distinctive metaloph of embrithopods is derived from this pattern mostly by the lingual migration of the metacone and related transverse hypertrophy of the premetacrista [41].

In *Abdounodus*, the separation of the metaconule from the protocone, which initiates the pseudohypocone development, is related to the regression of the postprotocrista and to the development of an interloph that extends lingually between protocone and metaconule and labially up to the mesostyle. The interloph development, alongside with pseudohypocone development, contributes to define the incipient protoloph and metaloph. The hypolophid occludes in the interloph between the protocone and pseudohypocone (Table 4). In other words, the early bunodont-bilophodont pattern is marked in *Abdounodus* upper molars by 1) the development of a pseudohypocone from the metaconule (quadrituberculy), 2) the transverse alignment of two independent rows of bunodont cusps (incipient proto- and metaloph), and 3) the development of the interloph between these incipient lophs. The character of the lower molars of *Abdounodus* that best illustrates the incipient bunodont-bilophodont pattern is the presence of a small hypolophid that occludes in the interloph, as indicated by occlusion and wear pattern (Tables 4 and 6; Fig 5).

The bunodont-bilophodont trend of *Abdounodus* is also evidenced functionally, perhaps even better than morphologically, by the pattern of occlusion and wear. It is characterized 1) by the occlusion of the hypolophid in the interloph, between the protocone and pseudohypocone, 2) by the occurrence of wear facets known in lophodont molars and which are new or modified with respect to tribosphenic-tritubercular molars (B8, B9, B10, B5?), 3) by the predominance of the attritional shearing of phase I [30] and especially of anterior/posterior shearing of upper and lower molars as is characteristic of lophodont taxa, and 4) also by the abrasion pattern which links the lingual and labial cusps, indicating a transverse motion of the lower jaw in the preparatory stroke of mastication.

However, *Abdounodus* shows a very early stage of the bunodont-bilophodont pattern, i.e., poorly specialized by comparison to crown paenungulates including the most basal ones such as *Eritherium* and *Seggeurius*. The pseudohypocone is smaller, the lophs less developed and marked mostly by alignment of bunodont cusps instead of transverse crests, and the protocone is less mesial (protoloph less developed) (Fig 7).

### Bunodonty

*Abdounodus* differs from *Ocepeia* by the pseudohypocone and related quadrituberculy, the higher bunodonty (*Ocepeia* retains shearing teeth), and the incipient lophs and interloph (absent in *Ocepeia)*. In *Abdounodus*, the bunodonty is associated with the remarkably long and robust tooth roots (Fig 3). The bunodonty of *Abdounodus* is a remarkable, shared trait with early crown paenungulates such as early proboscideans (*Eritherium*, *Phosphatherium*, *Numidotherium*), early hyracoids (*Seggeurius* and geniohyids), early embrithopods (*Palaeoamasia*) and early sirenians (*Protosiren*, *Eotheroides*, *Eosiren*, *Prototherium*). This indicates that bunodonty is a feature of the ancestral morphotype of the paenungulates. Later paenungulates evolved true lophodonty or selenodonty with sharp transverse crests and a higher crown. This is most characteristic in the proboscideans, advanced embrithopods such as *Arsinoitherium*, and some specialized clades of hyracoids (e.g., *Microhyrax*, *Dimaitherium*, titanohyracids, *Antilohyrax*).

### Structural scenario for the origin and evolution of the bilophodonty (upper molar pattern) of Paenungulata (Fig 8)

The discovery of the upper dentition of *Abdounodus* sheds first light on the homology and origin of the bilophodonty of the Paenungulata. *Ocepeia* and *Abdounodus* document for the first time intermediate structural stages between the primitive tribosphenic-tritubercular pattern of insectivore-like taxa such as *Todralestes* and the derived bilophodont pattern of the Paenungulata. In other words, *Ocepeia* and *Abdounodus* are the first fossil taxa showing the emergence of the bilophodont pattern of paenungulates.

*Ocepeia* and *Abdounodus* show key gradual, early structural steps toward the bilophodonty in a morphocline within which *Ocepeia* is closer to the primitive tribosphenic-tritubercular pattern. Primitive traits of *Ocepeia* include its low bunodonty, the metaconule poorly inflated and linked to the protocone, a residual but distinct lingual cingulum (but no hypocone), and the protocone less mesial (less aligned transversely with paracone).

Fig 8 summarizes the early structural steps illustrated by *Abdounodus* and *Ocepeia* in the evolution of the pseudohypocone (quadrituberculy) and bilophodonty of Paenungulata. With respect to the tribosphenic-tritubercular upper molar pattern, *Ocepeia* and *Abdounodus* show the following morphoclinal trend toward the paenungulate molar pattern:

1. increase in bunodonty (higher in *Abdounodus* than in *Ocepeia*);
2. lingual shift of the metaconule (stronger in *Abdounodus* than in *Ocepeia*), and its transverse alignment with metacone (*Abdounodus*);
3. enlargement of the metaconule (bulbous in *Abdounodus*);
4. separation of the metaconule from the protocone with regression of postprotocrista, and related development of the interloph (*Abdounodus*);
5. regression of postcingulum;
6. loss of paraconule, and no more labial conules.

### Convergence of lophodonty in Paenungulata (Afrotheria) and Perissodactyla (Euungulata, Laurasiatheria) (Fig 9)

**Fig 9:**
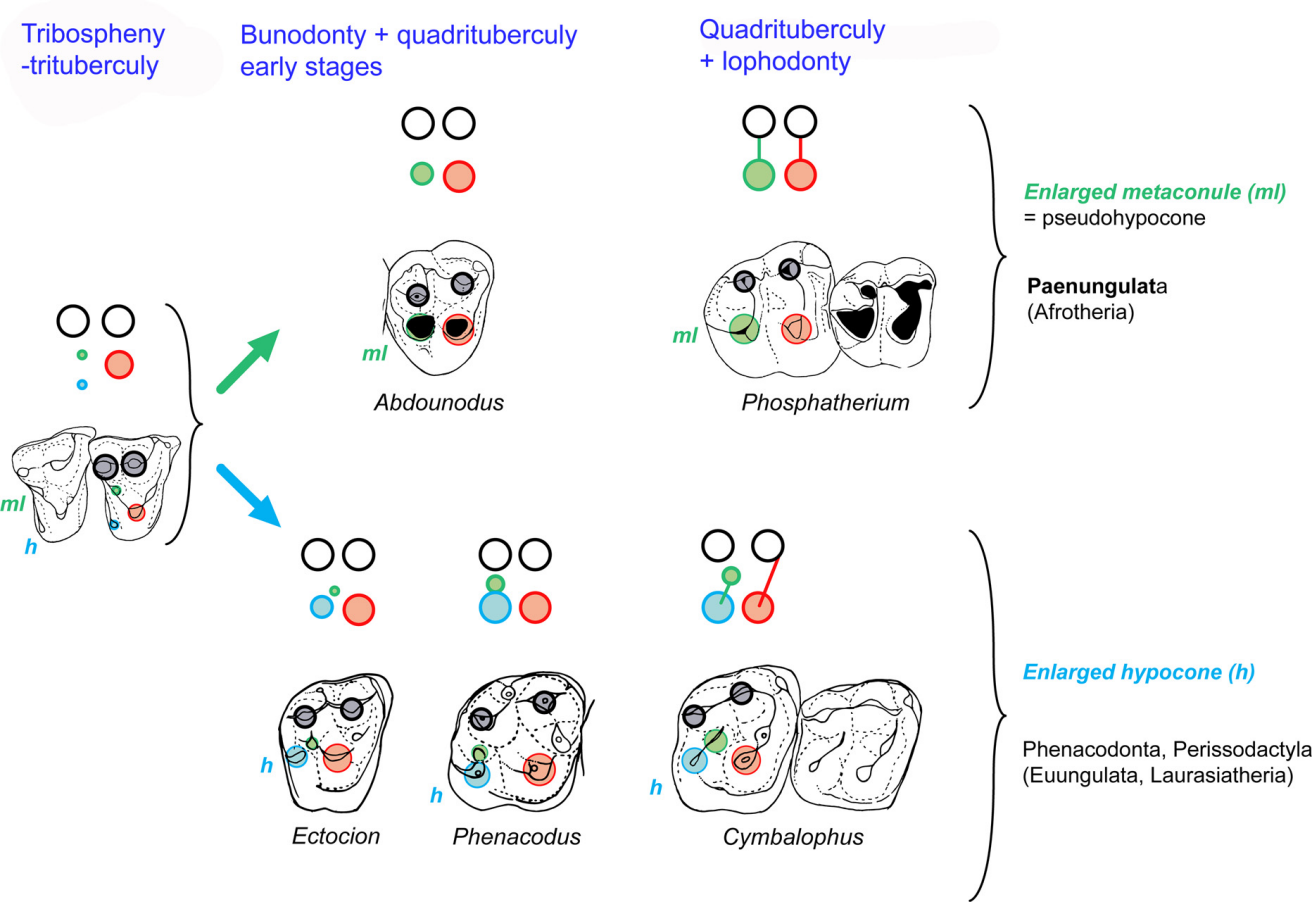
Convergence of lophodonty in Paenungulata (Afrotheria) and Perissodactyla (Euungulata, Laurasiatheria). The fourth upper molar cusp (postero-lingual cusp bearing the metaloph) is not homologous in Paenungulata and Perissodactyla: it is issued from a modified metaconule (pseudohypocone) in Paenungulata (*Phosphatherium* here) and from the true cingular hypocone in the Perissodactyla (*Cymbalophus* here) and early lophodont euungulates such as the Anthracobunidae (not shown here). Stem groups illustrating early stages of the evolution of the lophodonty remain poorly known in Euungulata, being represented mainly by phenacodontids such as *Ectocion* and *Phenacodus* that have a large hypocone. The stem paenungulates *Ocepeia* (not shown here) and *Abdounodus* are the first fossil taxa documenting intermediate morphological stages between the primitive tritubercular molar pattern and the derived quadritubercular and bilophodont molar pattern of the crown Paenungulata (here represented by *Phosphatherium*). Symbols of dental structures: Black circles: paracone and metacone, green circle: metaconule; blue circle: hypocone; red circle: protocone; transverse lines: proto- and metaloph. Occlusal sketch of teeth, all figured as right teeth.

Fig 9 summarizes the convergent evolution of the bilophodonty in paenungulates and euungulates such as the perissodactyls. It shows especially that the fourth upper molar cusp (metaloph cusp) is not homologous: it corresponds to the pseudohypocone (metaconule) in paenungulates, and to the true hypocone (cingular derived) in perissodactyls and extinct euungulate relatives such as anthracobunids and phenacodonts. It should be noted that among Euungulata, the stem groups documenting the emergence of bilophodonty of Perissodactyla are poorly known; transitional stages, as seen in *Abdounodus* and *Ocepeia*, remain unknown in Laurasian “condylarths” and euungulates. Phenacodonts and phenacolophids are for instance structurally more advanced than *Ocepeia* in the quadritubercular and bilophodont morphology.

Lophodont euungulates such as perissodactyls, and their extinct relatives such as anthracobunids, are as a whole distinctive from early paenungulates by the retention of a lingual cingulum and the enlarged hypocone (as opposed to its absence in Paenungulatomorpha), and well developed and labial conules.

## Phylogeny

The significant new material of *Abdounodus hamdii* described here allows the first study of its phylogenetic relationships among various placentals, but especially among paenungulates, afrotherians and euungulates. We have carried out a character analysis in order to test in particular (1) our hypothesis that *Ocepeia* and *Abdounodus* are transitional lophodont stem paenungulates and (2) our reconstructed morphocline of the characters linked to the evolution of quadrituberculy and lophodonty in the paenungulates and their relatives (Fig 8).

### Character/taxon matrix

The list of characters and the matrix analysed including *Abdounodus* are given in **S2 Text**; the character matrix in the Hennig and TNT format is provided in S3 Text. This matrix is derived and expanded from that of Gheerbrant et al. [21]. Changes were made for both in taxa and in features. In addition to the inclusion of *Abdounodus*, we expanded the taxonomic sample of basal euungulates (*Paschatherium*, *Cambaytherium*) and we removed the Zhelestidae that appeared unstable after pruned analysis with TNT. Changes in studied characters include several corrections and additions which are detailed in S1 Text. The main change in the characters coding is related to our resolved homology of the metaloph cusp, based on *Abdounodus* and *Ocepeia* morphology, as (1) a pseudohypocone (metaconule derived) in paenungulates, and (2) a true hypocone (postcingulum derived) in lophodont euungulates such as the perissodactyls and their relatives. It corresponds to the characters 97, 107, and 108. Because the homology of these characters remains questionable in desmostylians, they were coded with a question mark for the group. In this regard, our cladistic analysis provides a test of the secondary homology of the metaloph cusp in the desmostylians.

### Parsimony analyses

We used with the program TNT [42] for parsimony analyses and the interface Winclada [42, 43] for study of character distribution. We performed height cladistic analyses that are summarized in Table 7. All analyses use the ‘‘traditional search” command of TNT, with or without the TNT “implied weighting” option (IW). They were developed with the combined following different conditions (S2 Text, part III):

- 44 features ordered;
- all features (184) unordered;
- unweighted analysis;
- analysis with the TNT “implied weighting” option; it allows to decrease the value of homoplastic characters [44];
- the constrained clade including *Ocepeia*, *Abdounodus* and Paenungulata (= Paenungulatomorpha; see below);
- hypocone with increased weight: character 97 with weight x 5;
- Analysis excluding the Desmostylia to check the hypothesis of a long branch attraction with Paenungulata (i.e., convergence of Desmostylia and Paenungulata).

**Table 7:** Cladistic analyses of *Abdounodus* relationships performed in this work. ***Analysis 2*** (S2 Text, part III.2) ***is our reference analysis and tree in this work*.** All parsimony analyses were made with the “traditional search” command of TNT. Matrix with 27 taxa, 184 characters (S2 Text), 16 uninformative characters (inactived), 44 additive (ordered) characters (S6 Table). “Altungulata”: former grouping of lophodont ungulates including the Paenungulata and the crown and stem Perissodactyla; stem Perissodactyla: *Phenacolophus*, *Minchenella*, *Cambaytherium*, *Anthracobunia*, *Radinskya*.

Analysis	Type of analysis and constraint	Trees number	Trees Length	RI; CI	Comments
1.	“Traditional search”, 44 characters ordered, no constraint	1	735	53; 37	Clades: “Altungulata”; Hypocone strongly homoplastic; Good Bremer sup.
**2.**	**Idem 1 with “implied weighting“**	1	745	52; 36	Clades: **Paenungulatomorpha;** no Afrotheria. **Hypocone poorly homoplastic.**
3.	“Traditional search”, *no character ordered*, no constraint	2	682	52; 40	Clade “Altungulata”; basal clade (*Ocepeia*, *Abdounodus*); Good Bremer sup. for Paenungulata
4.	Idem 3 with “implied weighting”	1	691	52; 39	Clade “Altungulata” with sister-group *(Ocepeia*, *Abdounodus*).
5.	“Traditional search”, 44 characters ordered, clade Paenungulatomorpha constrained	4	743	52; 36	Clades: Paenungulatomorpha constrained, **Afrotheria** *minus* Macroscelidea; Desmostylia = stem Perissodactyla; Good Bremer sup.
6.	Idem 5 with “implied weighting”	1	749	52; 36	Clades: No Afrotheria; clade (Potam. (Orycter. Ptolem.)) basal; **Desmostylia = stem Perissodactyla**; **Hypocone poorly homoplastic**
7.	“Traditional Search”, 44 characters ordered, and hypocone with increased weight: K98 X 5	8	760	**57**; 37	Clades: **Paenungulatomorpha**; Afrotheria *minus* Macroscelidea distinct from Euungulata; **Desmostylia = stem Perissodactyla**; Low Bremer sup. **Hypocone not homoplastic**
8.	Idem 7 with “implied weighting”	1	768	56; 37	Clades: **Paenungulatomorpha**; no Afrotheria: (*Potam*. (*Orycter*. *Ptolem*.)) basal; *Todralestes* = sister group euungulates. **Hypocone not homoplastic**
9.	Analysis without Desmostylia, “Traditional search”, 44 characters ordered	18	711	56; 38	Clades: **Paenungulatomorpha**; some trees with Afrotheria.
10.	Idem 9 with “implied weighting”	1	718	55; 38	Clades: **Paenungulatomorpha**; no Afrotheria. **Hypocone poorly homoplastic.**

**Fig 10:**
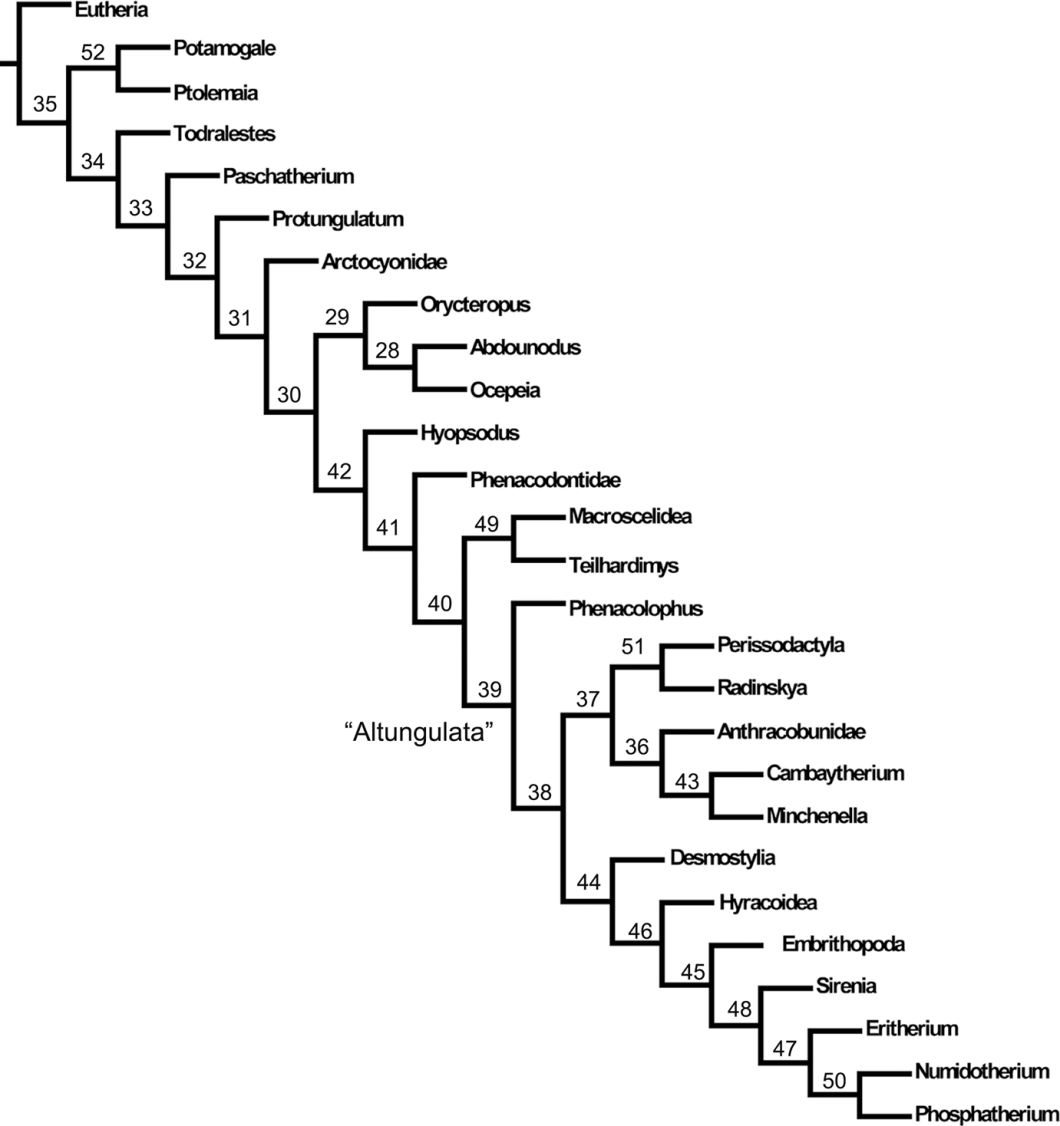
Relationships of *Abdounodus and paenungulates*. Most parsimonious tree resulting from the unweigheted analysis with ordered features. Details, Bremer index and distribution of synapomorphies are provided in S2 Text (part III.1). Tree length: 735. Retention index: 53. Consistency Index: 37.

The shortest most parsimonious trees (MPTs) were obtained for analyses of our matrix with unordered characters (S2 Text: cladograms 3–4). They yield similar topologies than the unweighted analysis of the matrix with ordered characters (Table 7; S2 Text, part III.1; Fig 10). They all recover a single clade of lophodont taxa (Paenungulata + Perissodactyla and their lophodont euungulates relatives), as was found in previous phylogenetic studies of *Ocepeia* and the early proboscideans *Eritherium* and *Phosphatherium* [20–21]. It corresponds to the taxon “Altungulata” ([13–14]; = “Pantomesaxonia” in Thewissen & Domning [45]), and is supported by characters mostly related to lophodonty, following the initial definition of McKenna & Manning [12]. Such a grouping of African and Laurasian lophodont ungulate-like placentals is, however, refuted by molecular phylogeny. In this topology *Abdounodus* and *Ocepeia* are in a basal position, out of the “Altungulata” (Fig 10, node 30), as was recovered by Gheerbrant *et al*. [21] for *Ocepeia*.

The likely convergence of the lophodonty in paenungulates and perissodactyls was already discussed based on some anatomical details [20–21]. It is now demonstrated by the dental morphology of *Abdounodus* described in this work which shows that the fourth upper molar cusp is not homologous, corresponding to the metaconule (pseudohypocone) in paenungulates.

**Fig 11:**
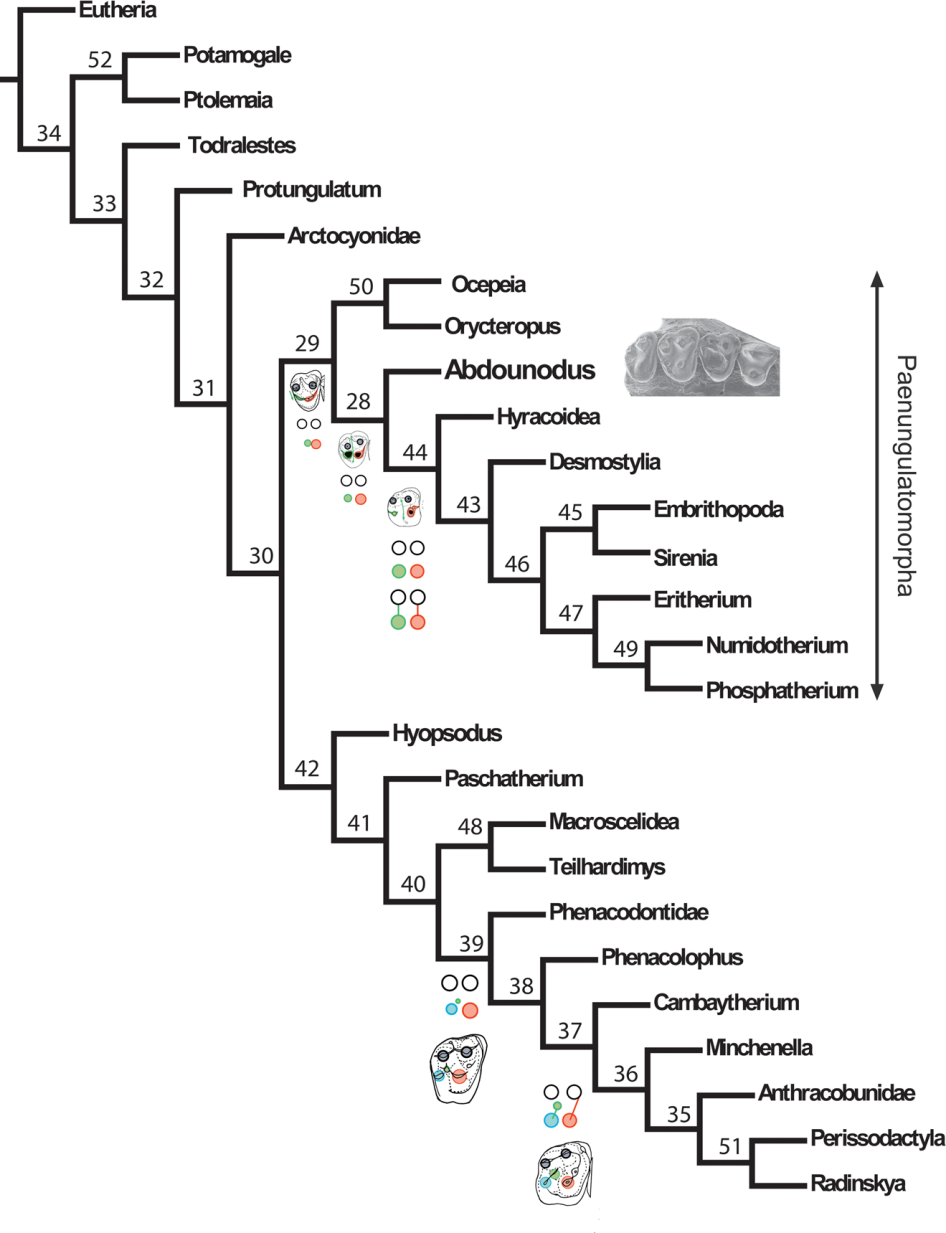
Relationships of *Abdounodus and paenungulates*. Most parsimonious tree resulting from analysis with implied weighting and ordered features. This tree is in this work our **reference topology** for the discussion of the relationships of *Abdounodus* and *Ocepeia* and of the distribution of the characters. Main upper molar patterns are outlined at the node of lophodont taxa (incl. early stages) to show the convergent development of the pseudohypocone in the Paenungulatomorpha and the hypocone in the Perissodactyla and other lophodont euungulates such as Anthracobunidae. See Fig 9 for caption of symbols of dental structures. Details, Bremer index and distribution of synapomorphies of this tree are provided in S2 Fig and S2 Text (part III.2). The clades numbers are reported above the nodes. Tree length: 746. Retention index: 52. Consistency Index: 33.

The trees recovering a single lophodont clade (“Altungulata”) are significantly shorter (Table 7: minimal difference is 10 steps shorter), but they paradoxically involve high homoplasy of key characters of lophodonty such as the hypocone (up to five extra steps in analyses 1 and 4) (S2 Table). In addition, they show very strong (i.e., costly) structural transformations in paenungulates, such as the regression in quadritubercular molars of the large hypocone (97–3 > 0) that is replaced by a large metaconule (107–2>4, 108–0>2). Analysis with implied weighting and ordered characters (S2 Text, part III.2) provides more consistent results in terms of both topology and character transformations. Its resulting single MPT (Fig 11) shows a stem relationship of *Abdounodus* and *Ocepeia* to paenungulates, and the convergent but less homoplastic development of the hypocone (1 extra-step) and pseudohypocone in euungulates and paenungulates. The resulting tree seen in Fig 11, which is in agreement with molecular phylogeny, is retained as the **reference topology in this work.** In this tree, other characters of lophodonty are convergent (homoplastic) in paenungulates and lophodont euungulates (Perissodactyla and stem relatives).

Analyses 7–8 (S2 Text: cladograms 7–8) make the hypocone non-homoplastic in MPTs. They were developed to test the significance of this character in the relationships of lophodont taxa and in the distribution (evolution) of key characters linked to lophodonty. They yield MPTs fully congruent with our reference analysis (Fig 11), and they have higher Retention Indices: they support the convergence of the lophodonty in Paenungulata and Perissodactyla, and show low homoplasy in the metaconule evolution (S2 Table). Analyses 5–6 (Table 7), that constrain a clade including *Abdounodus*, *Ocepeia* and paenungulates (= Paenungulatomorpha, see below), provide similar results.

Finally we have developed two analyses (Table 7: 9–10; S2 Text: cladograms 9–10) excluding the Desmostylia to check a possible effect of long branch attraction with the paenungulates. They also yield a congruent topology with our reference analysis (Fig 11).

### Results

As a whole, few MPTs with well-resolved consensus trees (i.e., with similar topologies) were recovered in our analyses. However, they are strongly homoplastic (Retention Indices RI = 52–57; shortest MPTs = 17 exclusive synapomorphies). Partitioned analyses show that the main phylogenetic signal of the matrix rests on dental features, as was found in the study of *Phosphatherium* and *Ocepeia* [18,21].

#### Topology and recovered clades

Our reference analysis illustrated Fig 11 makes *Ocepeia* and *Abdounodus* stem groups to crown Paenungulata in a single clade (node 29). This clade corresponds to the new taxon which is formally named here **Paenungulatomorpha** nov. magnorder. The topology and character distribution within the Paenungulatomorpha are in good agreement with our reconstructed morphocline of the evolution of pseudohypocone and lophodonty (Figs 8 and 9). The intermediate morphology and stem position of *Ocepeia* and *Abdounodus* to paenungulates are remarkably consistent.

The tubulidentate *Orycteropus* also joins the Paenungulatomorpha, usually as as the sister group of *Ocepeia*. However, *Orycteropus* has many inapplicable characters (46 dental features) of unknown homology with taxa included in the matrix because it is strongly specialized. For instance, it is unknown whether or not its simplified molar morphology was derived from an ancestral quadritubercular and bilophodont pattern. The main synapomorphy of *Orycteropus* and the Paenungulatomorpha, found in several trees, is the absence of a postglenoid foramen (174–2). Other shared traits with either *Ocepeia* or the Paenungulatomorpha are for most very homoplastic, and a few others are the result of optimization of inapplicable traits. It is however noted that a relationship of *Orycteropus* with paenungulates would support the clade Pseudoungulata [2,39] rather than the clade Afroinsectiphilia that is the sister group of the Paenungulata [4,46].

The clade Paenungulatomorpha is well distinguished from all other taxa in analyses with implied weighting (Fig 11, and S2 Text: cladograms 6 and 8). However, in unweighted analyses (S2 Text: cladograms 5 and 7), it belongs to a more inclusive clade including other African taxa such as *Potamogale* and *Ptolemaia*. Among them, *Potamogale* has the best supported relationship with Paenungulatomorpha (8 synapomorphies, although with low RI). Relationship of these afroinsectiphilian taxa with Paenungulatomorpha (in addition to *Orycteropus*) is strongly reminiscent of the clade Afrotheria. The main discrepancy of our MPTs from current definition of the Afrotheria is that macroscelideans are related to euungulates (Fig 11) instead of afrotherians (Afroinsectiphilia). This is mainly related to the presence of a large hypocone in macroscelideans (as for the louisinid *Teilhardimys*). It is however noted that two trees resulting from the analysis with the constrained clade Paenungulatomorpha (S2 Text: cladograms 5–0, 5–1) recover both the louisinids and macroscelideans forming the sister group of all other afrotherian taxa; they are characterized by a less homoplastic metaconule (S2 Table).

The Macroscelidea and the louisinid *Teilhardimys* are sister groups in our MPTs in a well supported node, in agreement with Tabuce et al. [47]. Both louisinids and macroscelideans were also previously considered as related to the Afrotheria [47–48], but Cooper et al. [49] alternatively found a stem relationship of the louisinids to perissodactyls and anthracobunids.

All analyses recover the Paenungulata. It is the best supported clade, with higher Bremer supports than in the study of *Ocepeia* [21], and with five exclusive synapomorphies in our reference analysis. Within Paenungulata, Hyracoidea is always basal. In the MPTs recovering the clade Paenungulatomorpha, the Sirenia and Embrithopoda are sister groups and form with Proboscidea the Tethytheria. *Eritherium* is sister group of other Proboscidea, except in unordered analyses (S2 Text: cladograms 3–4).

All our analyses associate the Perissodactyla and its sister group *Radinskya* in the same clade with the Anthracobunidae, *Cambaytherium*, and *Minchenella* (Fig 11, node 37). It supports that the anthracobunids are stem perissodactyls as recently concluded by Cooper et al. [49] and Rose et al. [50]. Anthracobunids and other early relatives of perissodactyls form either a single clade sister-group to Perissodactyla (Fig 10), corresponding to the infraorder Anthracobunia Ginsburg et al. 1999 [51], or they are arranged as a sequence of successive sister groups to the Perissodactyla (Fig 11). *Phenacolophus*, previously related to the Embrithopoda [12], is never allied to the Paenungulata in our MPTs. This is in agreement with its distinct enamel microstructure [30] from the Embrithopoda, and with new fossil data [52]. *Phenacolophus* is the basalmost stem perissodactyl in the MPTs recovering the clade Paenungulatomorpha (Fig 11), and it is more closely allied to lophodont euungulates than is the Phenacodontidae. The relationship of the Phenacodontidae to the Perissodactyla was recently supported by Halliday et al. [53]. These taxa share especially molarized premolars, by contrast to early paenungulatomorphans.

Some taxa have a notably unstable position in our analyses. This is especially true for the Desmostylia that is either related to Paenungulata (S2 Text: cladograms 1–4, 8) or to lophodont euungulates as stem perissodactyls. The relationship of Desmostylia to lophodont euungulates occurs in trees that optimise the presence of a hypocone in the group (secondary homology) and with low homoplasy of hypocone (S2 Table). It was also found recently by Cooper et al. [49]. The analysis excluding the Desmostylia, which was made in order to test a possible effect of long branch attraction with the Paenungulata (S2 Text: cladograms 9–10), supports the relationships of *Abdounodus* to the Paenungulata and the convergent evolution of the quadritubercular and bilophodont molars in the Paenungulatomorpha and Perissodactyla (plus extinct relatives).

#### Distribution and evolution of features

*Paenungulatomorpha*: The clade Paenungulatomorpha (Fig 11) displays the progressive development of quadritubercular and lophodont features such as the pseudohypocone (characters 107 & 108), reduced postprotocrista (110–1) and developed interloph (112–1), reduced postcristid (39–1), and other important traits such as the reduction of the paraconule (109–1) and of the postcingulum (98–0). The evolution of the pseudohypocone in Paenungulatomorpha corresponds to the following character states transformations (*asterisks denote non-homoplastic characters with RI = 100): node 29 (*Ocepeia* (*Abdounodus*, Paenungulata)): 107–2, *108–1; node 28 (*Abdounodus*, Paenungulata): *107–3; node 44 Paenungulata: *107–4, *108–2. This is in good agreement with our reconstructed morphocline and with the transitional morphology of *Ocepeia* and *Abdounodus* (Figs 7 and 8).

The evolution of a pseudohypocone is the landmark of the Paenungulatomorpha, but this clade is also characterized by other synapomorphies recorded in S3 and S4 Tables. The most important ones are unknown in lophodont euungulates and include:

- four synapomorphies shared by all paenungulatomorphans (Fig 11, node 29): *61–1 retromolar fossa present, 97–0 hypocone lost, 95–0 large stylar shelf, and 85–0 P^4^ postcingulum absent;
- two synapomorphies shared by *Abdounodus* and paenungulates (Fig 11, node 28): 109–1 reduced paraconule, 98–0 secondarily reduced molar postcingulum.

Other paenungulatomorphan synapomorphies such as the hypolophid are convergent with lophodont euungulates. The most remarkable one, known convergently only in *Phenacolophus* and Phenacodontidae, is the centrocrista dilambdodont and linked to an inflated mesostyle (99–1 & 103–1: selenodont ectoloph). It is known in *Ocepeia*, *Abdounodus* and early paenungulates such as *Eritherium*, *Phosphatherium*, *Seggeurius*, *Helioseus* and especially embrithopods such as *Namatherium* and *Palaeoamasia*. The presence of a mesostyle was recently recovered by Halliday *et al*. [53] as a synapomorphy of Phenacodontidae and Perissodactyla.

Within the Paenungulatomorpha it should be noted that *Abdounodus* is much less autapomorphic than *Ocepeia*. *Abdounodus* is in this regard significantly closer to the ancestral morphotype of extant paenungulates.

Paenungulata. The crown clade Paenungulata (Fig 11, node 44) is supported by as many as 25 features unknown in stem taxa such as *Ocepeia* and *Abdounodus* (S5 Table). They include however only five non-homoplastic synapomorphies unknown in lophodont euungulates: *1–1 I_1-3_ enlarged and procumbent, *59–1 coronoid foramen, *107–4 and *108–2 full pseudohypocone bearing the metaloph, *119–1 pseudohypocone root (S5 Table). The loss of a molar paraconid was recently found by Halliday *et al*. [53] as a synapomorphy of crown Euungulata, it is indeed a convergence of the Paenungulata with both the Cetartiodactyla and Perissodactyla.

*Afrotheria*: The relationship of some afroinsectiphilians taxa to paenungulatomorphans in some of our MPTs (S2 Text: cladograms 5, 7) is reminiscent of the clade Afrotheria. The best supported is found for *Potamogale*, although it is based on a few homoplastic characters. The most noticeable synapomorphies, which are rarely found in euungulates, are the followings: large I^1^ (64–1), small C_1_ (7–3), small to medium C^1^ (68–1), and possibly absence of molar postcingulum (98–0, Acctran). A large I^1^ is also a synapomorphy supporting the sister group relationship of macroscelideans and louisinids to *Potamogale* and Paenungulatomorpha, i.e., basically a synapomorphy of the Afrotheria.

## Conclusions

### Convergence of quadrituberculy and lophodonty in ungulate-like and afrotherian mammals

The discovery of the upper dentition of the poorly known early African ungulate-like genus *Abdounodus* [22] provides key morphological data on the origin and evolution of the lophodonty—a major adaptive feature—in the Paenungulata. The detailed comparative anatomy and functional analysis (occlusion and wear pattern) of the dentition of *Abdounodus*, *Ocepeia* and early crown paenungulates demonstrates that the fourth cusp of upper molars that bears the metaloph is unexpectedly not homologous to that of the Perissodactyla and its related lophodont euungulate taxa. The intermediate and progressive morphological stages documented by *Ocepeia* and *Abdounodus* show that paenungulates possess a metaconule-derived pseudohypocone, instead of the true cingular hypocone of the Perissodactyla and its lophodont euungulate relatives. This is the first morphological and fossil evidence of the dental convergence of the African and the Laurasian ungulate-like placentals.

We investigated the phylogenetic significance of the intermediate morphology of *Abdounodus* in a cladistic analysis including early paenungulates, afrotherians and various euungulates and eutherians. The analysis using ordered characters and implied weighting (Fig 11) supports for the first time the relationship of *Ocepeia* and *Abdounodus* to the crown paenungulates, as stem taxa of a major new clade—the Paenungulatomorpha nov. magnorder. Alternative topologies (unordered and unweighted analyses) cluster the Perissodactyla and Paenungulata in the same clade, corresponding to the obsolete taxon “Altungulata” that is refuted especially by the molecular phylogeny. These alternative trees do not provide clear evidence for the convergence of the lophodonty, and they imply the unlikely reversal of a large true hypocone in paenungulates. They are therefore rejected here. The character transformation entailed by the MPT resulting from our reference analysis with implied weighting (Figs 11 and S2) is more consistent and confirms the transitional morphology of *Ocepeia* and *Abdounodus* toward the paenungulate quadrituberculy and bilophodonty, and the key metaconule contribution to this dental pattern, instead of the hypocone. This is congruent with our reconstructed morphocline of character transformation linked to the evolution of bilophodonty in Paenungulatomorpha (Figs 7 and 8).

Although more basal within Paenungulatomorpha, *Ocepeia* is noticeably more autapomorphic than *Abdounodus*. In this regard, *Ocepeia* and its family [21] represent an older, specialized lateral branch of Paenungulatomorpha, and *Abdounodus* is significantly closer to the ancestral morphotype of the crown-group Paenungulata.

Our phylogenetic analysis is congruent with Copper *et al*.’s [49].and Rose *et al*.’s [50] studies showing the stem relationship to Perissodactyla of the early lophodont Laurasian taxa such as the Anthracobunidae and *Phenacolophus*, that is supported by the presence of a large true hypocone. It is also congruent with the relationship of the Phenacodontidae to the Perissodactyla, both taxa also sharing a large hypocone (e.g., [53]). The order Desmostylia has a more ambiguous position. It is related to the paenungulates in our reference analysis, but it is alternatively a stem Perissodactyla in the MPTs optimizing the presence of a hypocone in the group (S2 Text: cladograms 5–8). The cladistic analysis supports that the Desmostylia actually shows a case of long-branch attraction with the Paenungulata, contributing to the erroneous inclusion of the Paenungulata and Perissodactyla in the same clade (“Altungulata”) based on shared bilophodont molars.

The African condylarth-grade mammals *Abdounodus* and *Ocepeia* are the first known transitional fossil taxa demonstrating that the lophodont molar pattern is not homologous in African and Laurasian ungulate-like placentals. They elucidate the first well-supported morphological synapomorphy within Afrotheria—namely the **pseudohypocone** (characters 107 and 108) of the Paenungulata—that evidences the convergence between the Paenungulata and the Perissodactyla (Euungulata). This is congruent with especially the molecular analyses that argue for separate phylogenetic and paleobiogeographic evolution of the clades Afrotheria-Paenungulata and Laurasiatheria-Eungulata (e.g., [5,46]). This is also consistent with the recent morphological phylogeny published by Halliday *et al*. [53] showing that all Laurasian “condylarths”, such as the Phenacodontidae, belong to the Laurasiatheria.

The presence of a pseudohypocone in the paenungulates is identified here based on the comparative anatomy of the teeth of *Abdounodus* and of *Ocepeia*. This emphasizes the importance of the early fossils to elucidate the convergences and homologies that cannot be solved or are even *unexpected* based on modern taxa alone. This is especially true for the Afrotheria and the Paenungulata that accumulated strong homoplasies and specializations during their rapid radiation and long evolutionary history [54–56]. The intermediate morphology of the early stem taxa *Abdounodus* and *Ocepeia* sheds new light on the ancestral state and evolution of key dental features of the Paenungulata such as the quadrituberculy and bilophodonty, and it helps to clarify their phylogenetic significance. It should be noted that, although the early Tertiary fossil record of Laurasia is much better known than that of Africa, the origin of the bilophodonty in euungulates remains poorly known.

The afrotherian order Macroscelidea, which is the best documented afroinsectiphilian group, has a quadritubercular and incipient bunodont-lophodont molar pattern that resembles early paenungulates such as *Eritherium* or *Seggeurius*. It is also found in the genera *Louisina* and *Teilhardimys* of the European family Louisinidae that is related to the Macroscelidea by some authors [47–48]. Our anatomical comparisons indicate that this pattern both in the Macroscelidea and Louisinidae actually results from the presence a large true hypocone and is not homologous with the Paenungulatomorpha. It indicates a convergence of this ungulate-like pattern also within the Afrotheria, rather than its inheritance from a common afrotherian ancestor. This is unexpected because the Paenungulata and the Macroscelidea are both included in the clade Afrotheria that is supported by molecular data and by some morphological evidences [47,53]. In most of our MPTs recovering the Paenungulatomorpha, the Macroscelidea is indeed clustered with euungulates (Fig 11). However, some analyses recover a sister group relationship of the macroscelideans to *Potamogale* and Paenungulatomorpha, i.e., they recover their relationship to the Afrotheria, and they assume the parallel evolution of the pseudohypocone and hypocone within the Afrotheria.

### Significance of early paenungulatomorphans for placental phylogeny and radiation

*Abdounodus* and *Ocepeia* are the only known representatives of the early radiation of the African ungulate-like mammals predating the divergence of extant paenungulate orders, and both are related to their common ancestor. This stands in contrast to the huge diversity of the Laurasian “condylarths” that includes not only stem groups of the modern lineages (e.g., euungulates), but also a variety of extinct groups. Nevertheless, the co-occurrence of the autapomorphic *Ocepeia* with *Abdounodus* and *Eritherium* in the Selandian of Morocco indicates the presence and old endemic evolutionary history of placentals in Africa that remains nearly blank except for the few Moroccan discoveries. The Paenungulatomorpha evolved at least from the early Tertiary onwards, and independently from the Laurasian basal euungulates and “condylarths” such as the phenacodontids and apheliscids. *Abdounodus* and *Ocepeia* emphasize the importance of the early African fossil record for the knowledge of the phylogeny and early radiation of paenungulates and afrotherians.

The rapid radiation of the Afrotheria and Paenungulatomorpha at the beginning of the Tertiary, as illustrated by the Paleocene Moroccan mammal fauna, is concurrent with that of the Laurasiatheria [53,56], in a general explosive evolution of mammals in both the South and North Tethyan continents after the global K/Pg event.

## Supporting Information

S1 Fig 3D model of the maxillary of *Abdounodus hamdii* (Selandian, Ouled Abdoun, Morocco), reconstructed from the CT scans of the specimen MHNM.KHG.154. The reconstruction illustrates the sutured right and left maxillaries of the original specimen MHNM.KHG.154 with the jugal dentition (P^3-4^, M^1-3^). The length of the preserved tooth row P3-M3 is 23.8 mm (right tooth row) and 22.4 mm (left tooth row).
(PDF)

S2 Fig Most parsimonious tree with synapomorphies resulting from analysis with implied weighting and ordered features (see Fig 11). This tree is our reference topology for the discussion of the relationships of *Abdounodus* and *Ocepeia* and of the distribution of the characters. Details on the analysis and synapomorphies in this tree are provided in S2 Text (part III.2). The black and open white circles represent respectively strict and homoplastic synapomorphies. Tree length: 746. Retention index: 52. Consistency Index: 36.5.
(JPG)

S1 Table Angle of molar wear striae of *Abdounodus hamdii* for reconstruction of its mastication compass (Fig 6C). (DOC)

S2 Table Homoplasy scores (extra steps) of some key features of upper molars seen in *Abdounodus*, *Ocepeia* and the Paenungulata. Clades found in our Most Parsimonious Trees: Alt: lophodont ungulates (“Altungulata”); Pae: Paenungulatomorpha.
(DOC)

S3 Table Synapomorphies of Paenungulatomorpha (Fig 11, node 29: *Ocepeia* (*Abdounodus*, Paenungulata)). Ambiguous synapomorphies: (a) Acctran optimization; (d) Deltran optimization.
(DOC)

S4 Table Synapomorphies of *Abdounodus* and Paenungulata (Fig 11, node 28). Ambiguous synapomorphies: (a) ACCTRAN optimization; (d) DELTRAN optimization.
(DOC)

S5 Table Synapomorphies of Paenungulata (Fig 11, node 44). Ambiguous synapomorphies: (a) ACCTRAN optimization; (d) DELTRAN optimization.
(DOC)

S6 Table Additive characters in analysed matrix of *Abdounodus hamdii*. (DOC)

S1 Text Changes (additions and corrections) made in the character matrix of Gheerbrant et al. (2014) for the analysis of the relationships of *Abdounodus* (DOC)

S2 Text Character matrix and phylogenetic analysis of *Abdounodus*. (PDF)

S3 Text TNT/Hennig character matrix analysed for cladistic study of the relationships of *Abdounodus*. (SS)

## References

[1] Stanhope MJ, Waddell VG, Madsen O, Dejong WW, Hedges S B, Cleven GC, et al Molecular evidence for multiple origins of the Insectivora and for a new order of endemic African mammals. Proc. Ntl. Acad. Sc., USA 1998; 95: 9967–9972. 10.1073/pnas.95.17.9967 PMC21445 9707584

[2] Waddell PJ, Okada N, Hasegawa M. Towards resolving the interordinal relationships of placental mammals. Syst. Biol. 1999; 48: 1–5. 12078634

[3] Madsen O, Scally M, Douady CJ, Kao DJ et al Parallel adaptive radiations in two major clades of placental mammals. Nature 2001; 409:610–614. 11214318 10.1038/35054544

[4] Murphy WJ, Eizirik E, Johnson WE, Zhang YP, Ryder OA, O’brien SJ. Molecular phylogenetics and the origin of placental mammals. Nature 2001; 409: 614–618. 11214319 10.1038/35054550

[5] Springer MS, Stanhope J, Madsen O, Dejong WW. Molecules consolidate the placental mammal tree. Trends Ecol. Evol. 2004; 19: 430–438. 16701301 10.1016/j.tree.2004.05.006

[6] Prothero D. Ungulate phylogeny: molecular vs. morphological evidence In: Szalay FS, McKenna MC, editors. Mammal Phylogeny: vol. II: Placentals. New York: Springer Verlag; 1993 pp. 173–181.

[7] Hooker JJ. Perissodactyla In: Rose KD, Archibald JD, editors. The Rise of Placental Mammals: Origins and Relationships of the Major Extant Clades Baltimore: Johns Hopkins University Press; 2005 pp. 199–214.

[8] Rose KD. The Beginning of the Age of Mammals. Baltimore: Johns Hopkins University Press; 2006.

[9] Welker F, Collins JM, Thomas JA, Wadsley M, Brace S, Cappellini E et al Ancient Proteins Resolve the Evolutionary History of Darwin’s South American ungulates. Nature 2015; 522: 81–84. 10.1038/nature14249 25799987

[10] Linnaeus, C. Systemae naturae. 12th ed. 1766.

[11] Simpson GG. The principles of classification and a classification of mammals. Bull. Am. Mus. Nat. Hist. 1945; 85: 1–350.

[12] McKenna M C, Manning E. Affinities and paleobiogeographic significance of the Mongolian Paleogene genus *Phenacolophus*. Geobios 1977; Mém. Sp. 1, 1: 61–85.

[13] Prothero DR, Schoch RM. Origin and evolution of the Perissodactyla: summary and synthesis In: Prothero D R, Schoch R M, editors. The Evolution of Perissodactyls. New York: Oxford University Press; 1989 pp. 504–529

[14] McKenna MC, Bell SK. Classification of Mammals above the Species Level New York: Columbia University Press 1997.

[15] Arambourg C. Les vertébrés fossiles des gisements de phosphates (Maroc-Algérie-Tunisie). N. Mém. Serv. Géol. Maroc 1952; 92: 1–372.

[16] Noubhani A, Cappetta H. Les Orectolobiformes, Carcharhiniformes et Myliobatiformes (Elasmobranchii, Neoselachii) des bassins à phosphate du Maroc (Maastrichtien–Lutétien basal). Systématique, biostratigraphie, évolution et dynamique des faunes. Palaeo Ichthyol. 1997; 8: 1–327.

[17] Gheerbrant E, Sudre J, Cappetta, H. A Palaeocene proboscidean from Morocco. Nature 1996; 383, 68–71.

[18] Gheerbrant E, Sudre J, Tassy P, Amaghzaz M, Bouya B, Iarochene M. Nouvelles données sur *Phosphatherium escuilliei* (Mammalia, Proboscidea) de l’Eocène inférieur du Maroc, apports à la phylogénie des Proboscidea et des ongulés lophodontes. Geodiversitas 2005; 27: 239–333.

[19] Gheerbrant E, Bouya B, Amaghzaz M. Dental and cranial anatomy of *Eritherium azzouzorum* from the Paleocene of Morocco, earliest known proboscidean mammal. Palaeontogr. Abt A, 2012; 297: 151–183.

[20] Gheerbrant E. Paleocene emergence of elephant relatives and the rapid radiation of African ungulates. Proc. Natl. Acad. Sci., USA 2009; 106: 10717–10721. 10.1073/pnas.0900251106 19549873 PMC2705600

[21] Gheerbrant E, Amaghzaz M, Bouya B, Goussard F, Letenneur C. *Ocepeia* (Middle Paleocene of Morocco): the oldest skull of an afrotherian mammal. PLoS ONE 2014; 9 (2), e89739 10.1371/journal.pone.0089739 24587000 PMC3935939

[22] Gheerbrant E, Sudre J, Iarochene M, Moumni A. First ascertained African "condylarth" mammals (primitive ungulates: cf. Bulbulodentata & cf. Phenacodonta) from the Earliest Ypresian of the Ouled Abdoun Basin, Morocco. J. Vert. Pal. 2001; 21: 107–118.

[23] Gheerbrant E. Primitive African ungulates ("Condylarthra" and Paenungulata) In: Werdelin L Sanders WJ, editors. Cenozoic Mammals of Africa. Berkeley, Los Angeles, London: The University of California Press; 2010 pp. 563–571.

[24] Yans J, Amaghzaz M, Bouya B, Cappetta H, Iacumin P, Kocsis L et al First carbon isotope chemostratigraphy of the Ouled Abdoun phosphate Basin, Morocco; implications for dating and evolution of earliest African placental mammals. Gondwana Res. 2014; 25: 257–269.

[25] Kocsis L, Gheerbrant E, Mouflih M, Cappetta H, Yans J, Amaghzaz M. Comprehensive stable isotope investigation of marine biogenic apatite from the late Cretaceous—early Eocene phosphate series of Morocco. Palaeogeogr., Palaeoclim., Palaeoecol. 2014; 394: 74–88.

[26] Kavanagh KD, Evans AR, Jernvall J. Predicting evolutionary patterns of mammalian teeth from development. Nature 2007; 449:427–433. 17898761 10.1038/nature06153

[27] Polly PD. Development with a bite. Nature 2007; 229:413–415. 10.1038/449413a 17898756

[28] Wilson LA, Madden RH, Kay RF, Sánchez-Villagra MR. Testing a developmental model in the fossil record: molar proportions in South American ungulates. Paleobiol. 2012; 38, 308–321.

[29] Kullmer O, Benazzi S, Fiorenza L, Schulz D, Bacso S, Winzen O. Technical Note: Occlusal Fingerprint Analysis (OFA): Quantification of tooth wear pattern. Amer. J. Phys. Anthr. 2009; 139: 600–605. 10.1002/ajpa.21086 19425091

[30] Koenigswald Von W, Anders U, Engels S, Schultz JA, Kullmer O. Jaw movement in fossil mammals: analysis, description and visualization. Paläont. Zeit. 2013; 87: 141–159, 10.1007/s12542-012-0142-4

[31] Crompton AW. The origin of the tribosphenic molar. In: D.M. Kermack & K.A. Kermack, editors. Early Mammals. New York: Academic Press. Zool. J. Lin. Soc. 1971; 50, suppl. 1: 65–87.

[32] Butler PM. The milk-molars of Perissodactyla, with remarks on molar occlusion. Proc. Zool. Soc. 1952; 121, 777–817.

[33] Hooker JJ. A primitive ceratomorph (Perissodactyla, Mammalia) from the early Tertiary of Europe. Zool. J. Lin. Soc.1984; 82: 229–244.

[34] Cote S, Werdelin L, Seiffert ER, Barry J C. Additional material of the enigmatic early Miocene mammal *Kelba* and its relationship to the order Ptolemaiida. Proc. Natl. Acad. Sci., USA 2007; 104: 510–5515. 10.1073/pnas.0700441104 PMC1838468 17372202

[35] Mahboubi M, Ameur R, Crochet J-Y, Jaeger J-J. El Kohol (Saharan Atlas, Algeria): A new Eocene mammal locality in Northwestern Africa. Palaeontogr. 1986; 192: 15–49.

[36] Court N, Mahboubi M. Reassessment of Lower Eocene *Seggeurius amourensis*—aspects of primitive dental morphology in the mammalian order Hyracoidea. J. Pal. 1993; 67: 889–893.

[37] Benoit J, Crochet J-Y, Mahboubi M, Jaeger J-J, Bensalah M, Adaci M et al New material of *Seggeurius amourensis* (Paenungulata, Hyracoidea) including a partial skull with intact basicranium. J. Vert. Pal. 2015; 36 (2), in press. 10.1080/02724634.2015.1034358

[38] Gheerbrant E, Sudre J, Cappetta H, Mourer-Chauvire C, Bourdon E, Iarochene M et al Les localités à mammifères des carrières de Grand Daoui, Bassin des Ouled Abdoun, Maroc, Yprésien: Premier état des lieux. Bull. Soc. Géol. France 2003; 174: 279–293.

[39] Seiffert ER. A new estimate of afrotherian phylogeny based on simultaneous analysis of genomic, morphological, and fossil evidence. BMC Evol. Biol. 2007; 7, 224, 1–13. 17999766 10.1186/1471-2148-7-224 PMC2248600

[40] Gunnell GF, Gingerich PD, Holroyd PA. Ptolemaiida In: Werdelin L, Sanders WJ, editors. Cenozoic Mammals of Africa. Berkeley, Los Angeles, London: The University of California Press; 2010 pp.123–145

[41] Court N. A unique form of dental bilophodonty and a functional interpretation of peculiarities in the masticatory system of *Arsinoitherium* (Mammalia, Embrithopoda) Hist. Biol. 1992; 6: 91–111.

[42] Goloboff PA, Farris JS, Nixon KC. TNT, a free program for phylogenetic analysis. Cladistics 2008; 24: 774–786

[43] Nixon KC. Winclada (BETA) Version 0.9.9. Software published by the author, Ithaca, N.Y. 1999.

[44] Goloboff PA. Estimating character weights during tree search. Cladistics 1993; 9, 83–91. 10.1111/j.1096-0031.1993.tb00209.x 34929936

[45] Thewissen JGM, Domning D. The role of phenacodontids in the origin of the ING, D. P modern orders of ungulate mammals. J. Vert. Pal. 1992; 12: 494–504.

[46] Robinson TJ, Ruiz-Herrera A, Avise JC. Hemiplasy and homoplasy in the karyotypic phylogenies of mammals. Proc. Natl. Acad. Sci., USA 2008; 105: 14477–14481. 10.1073/pnas.0807433105 18787123 PMC2567171

[47] Tabuce R, Marivaux L, Adaci M, Bensalah M, Hartenberger JL, Mahboubi M et al Early Tertiary mammals from North Africa reinforce the molecular Afrotheria clade. Proc. Roy. Soc. London 2007; B, 274: 1159–1166. 10.1098/rspb.2006.0229 PMC2189562 17329227

[48] Hooker JJ, Russell DE. Early Palaeogene Louisinidae (Macroscelidea, Mammalia), their relationships and north European diversity. Zool. J. Lin. Soc. 2012; 164: 856–936. 10.1111/j.1096-3642.2011.00787.x

[49] Cooper L, Seiffert ER, Clementz M, Madar SM, Bajpai S, Hussain ST et al Anthracobunids from the middle Eocene of India and Pakistan are stem Perissodactyls. PLoS One 2014; 9(10), e109232 10.1371/journal.pone.0109232 25295875 PMC4189980

[50] Rose KD, Holbrook L T, Rana RS, Kumar K, Jones KE, Ahrens HE, Missiaen P, Sahni A, Smith T. Early Eocene fossils suggest that the mammalian order Perissodactyla originated in India. Nature Comm. 2014; 5: 1–9. 10.1038/ncomms6570 25410701

[51] Ginsburg L, Durrani KH, Kassi M, Welcomme J-L. Discovery of a new Anthracobunidae Tethytheria, Mammalia) from the Lower Eocene lignite of the Kach-Harnai Area in Baluchistan (Pakistan) C. R. Acad. Sci. Paris sér. IIa, 1999; 328: 209–213.

[52] Mao F-Y Wang Y-Q Li Q, Jin X. New records of archaic ungulates from the Lower Eocene of Sanshui Basin, Guangdong, China. Hist. Biol. 2016; 28: 787–802.

[53] Halliday TJD, Upchurch P, Goswami A. Resolving the relationships of Paleocene placental mammals. Biological Rev. 2015 10.1111/brv.12242 PMC6849585 28075073

[54] Robinson TJ, Seiffert ER. Afrotherian origins and interrelationships: New views and future prospects. Cur. Top. Dev. Biol. 2004; 63: 37–60. 10.1016/S0070-2153(04)63002-X 15536013

[55] Pardini AT, O'brien PC, Fu B, Bonde RK, Elder FF, Ferguson-Smith MA et al Chromosome painting among Proboscidea, Hyracoidea and Sirenia: support for Paenungulata (Afrotheria, Mammalia) but not Tethytheria. Proc. Biol. Sc. B 2007; 274: 1333–1340. 10.1098/rspb.2007.0088 PMC1914331 17374594

[56] Halliday TJD, Goswami A. Eutherian morphological disparity across the end-Cretaceous mass extinction. Biol. J. Lin. Soc. 2016; 118: 152–168.

