# Supplementary figures and images for: Convergence of Afrotherian and Laurasiatherian Ungulate-Like Mammals: First Morphological Evidence from the Paleocene of Morocco
Source: PLoS One. 2016 Jul 6;11(7):e0157556. doi: 10.1371/journal.pone.0157556 (PMC4934866; doi:10.1371/journal.pone.0157556)

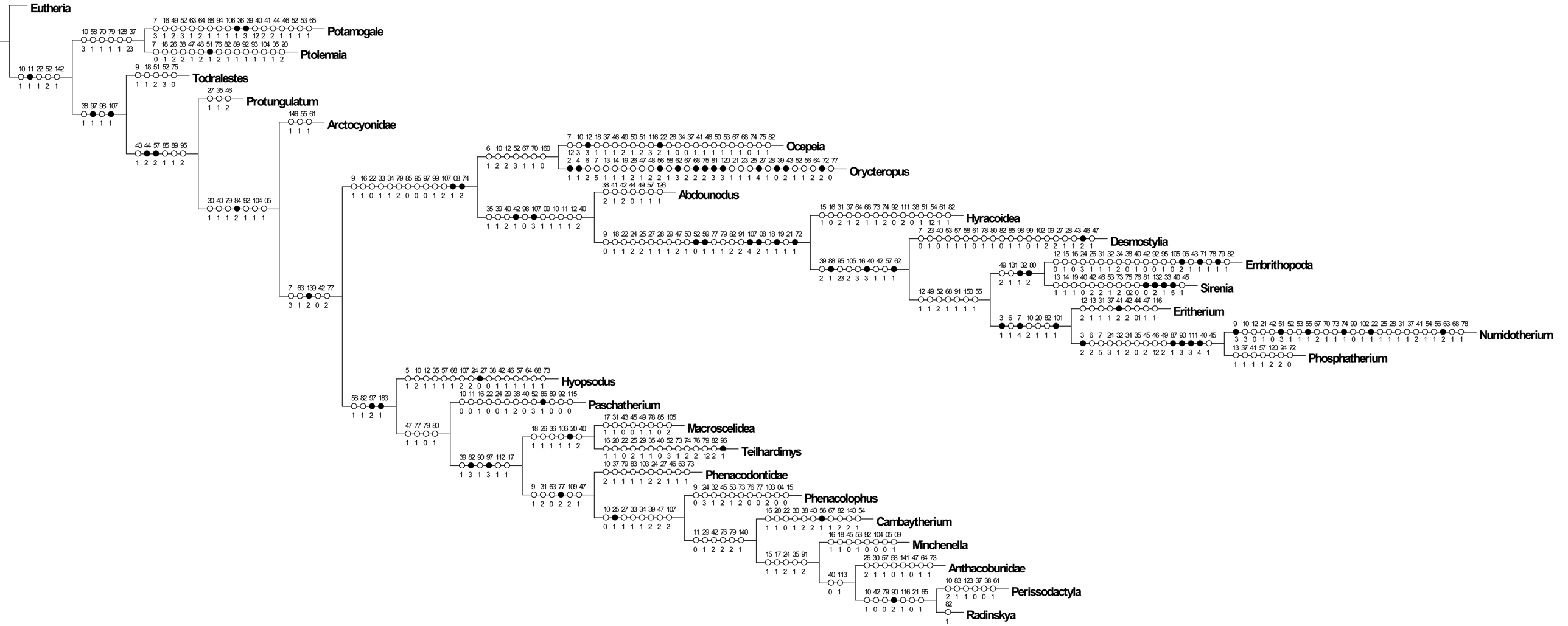

Supplement: S2 Fig — This tree is our reference topology for the discussion of the relationships of Abdounodus and Ocepeia and of the distribution of the characters. Details on the analysis and synapomorphies in this tree are provided in S2 Text (part III.2). The black and open white circles represent respectively strict and homoplastic synapomorphies. Tree length: 746. Retention index: 52. Consistency Index: 36.5. (JPG) [file pone.0157556.s002.jpg]
