# Supplementary material for: Convergence of Afrotherian and Laurasiatherian Ungulate-Like Mammals: First Morphological Evidence from the Paleocene of Morocco
Source: PLoS One. 2016 Jul 6;11(7):e0157556. doi: 10.1371/journal.pone.0157556 (PMC4934866; doi:10.1371/journal.pone.0157556)
Supplement: S1 Table — (DOC) [file pone.0157556.s003.doc]

S1 Table. Angle of the molar wear striae of *Abdounodus hamdii* for reconstruction of its mastication compass (**Fig. 6C**)

|  | Phase I | Phase II |
| --- | --- | --- |
| Inclination *vs* horizontal axis | 30-40 ° | Absent |
| Direction *vs* mesio-distal axis | 80-70 °  lingual, lingo-linguomesial | Absent |
