# Supplementary material for: Convergence of Afrotherian and Laurasiatherian Ungulate-Like Mammals: First Morphological Evidence from the Paleocene of Morocco
Source: PLoS One. 2016 Jul 6;11(7):e0157556. doi: 10.1371/journal.pone.0157556 (PMC4934866; doi:10.1371/journal.pone.0157556)
Supplement: S2 Table — Clades found in our Most Parsimonious Trees: Alt: lophodont ungulates (“Altungulata”); Pae: Paenungulatomorpha. (DOC) [file pone.0157556.s004.doc]

S2 Table. Homoplasy scores (extra steps) of some key features of upper molars seen in *Abdounodus*, *Ocepeia* and the Paenungulata. Clades found in our Most Parsimonious Trees: Alt : lophodont ungulates (“Altungulata”); Pae: Paenungulatomorpha.

| Characters /  Analysis | 97 -  hypocone | 99 -  mesostyle | 103 -  centro-crista | 107 - meta-conule | 108 - meta-conule | Clades |
| --- | --- | --- | --- | --- | --- | --- |
| 1 | 5 | 4 | 3 | 2 | 1 | Alt |
| 2 | 1 | 4 | 2 | 2 | 0 | Pae |
| 3 | 3,3 | 4,4 | 3,3 | 3,3 | 0,0 | Alt |
| 4 | 5 | 3 | 2 | 3 | 0 | Alt |
| 5 | 3,3,2,2 | 3,3,3,3 | 2,2,2,2 | 3, 3,4,4 | 0,0,0,0 | Pae |
| 6 | 1 | 3 | 2 | 2 | 0 | Pae |
| 7 | 0,0,0,0,0,0,0,0 | 3,3,3,3,3,3, 3,3 | 2,2,2,2,2,2,2,2 | 3,3,3,3,3,3,3,3 | 0,0,0,0,0,0,0,0 | Pae |
| 8 | 0 | 4 | 2 | 2 | 0 | Pae |
| 9 | 2,2,2,2,3,3,3,3,3,3,3,3,3,2,2,2,2,3,3 | 3,3,3,3,3,3,3,3,3,3,3,3,3,3,3,3,3,3,3 | 2,2,2,2,2,2,2,2,2,2,2,2,2,2,2,2,2,2,2 | 2,2,2,3,3,3,3,3,3,3,3,3,3,3,3,3,3,3,3 | 2,2,2,0,2,2,2,0,0,0,0,0,2,0,0,0,0,0,0 | Pae |
| 10 | 1 | 3 | 2 | 2 | 0 | Pae |
