# Supplementary material for: Convergence of Afrotherian and Laurasiatherian Ungulate-Like Mammals: First Morphological Evidence from the Paleocene of Morocco
Source: PLoS One. 2016 Jul 6;11(7):e0157556. doi: 10.1371/journal.pone.0157556 (PMC4934866; doi:10.1371/journal.pone.0157556)
Supplement: S3 Table — Ambiguous synapomorphies: (a) Acctran optimization; (d) Deltran optimization. (DOC) [file pone.0157556.s005.doc]

S3 Table. Synapomorphies of the Paenungulatomorpha (Fig 11, node 29 : *Ocepeia* (*Abdounodus*, Paenungulata)). Ambiguous synapomorphies: (a) acctran optimization; (d) DELtran optimization. * denotes a non-homoplastic synapomorphy.

| Character state | RI | Description | Remarks |
| --- | --- | --- | --- |
| 16-1 | 36 | P_3_ paraconid absent | Convergence with Euungulates |
| 33-1 | 90 | Hypolophid present | Convergence with lophodont Euungulates |
| 34-1 | 83 | Hypolophid notched | Convergence with lophodont Euungulates |
| ***61-1** | **100** | **Retromolar fossa present** | ***Not in Euungulates*** |
| **85-0** | **70** | **P^4^ Postcingulum reduced** | ***Not in Euungulates*** |
| **95-0** | **42** | **Large stylar shelf** | ***Not in Euungulates***  **Not in derived Paenungulata** |
| **97-0** | **96** | **Hypocone secondarily reduced** | ***Not in Euungulates*** |
| 99-1 | 42 | Mesostyle present | Convergence with lophodont Euungulates |
| 103-1 (a) | 60 | Centrocrista dilambdodont | Convergence with lophodont Euungulates |
| 107-2 | 90 | Metaconule large | Some lophodont Euungulates |
| ***108-1** | **100** | **Metaconule lingually close to protocone** | ***Not in Euungulates*** |
| 174-2 | 66 | Postglenoid foramen absent | Not in *Ocepeia* & *Phosphatherium* |
