# Supplementary material for: Convergence of Afrotherian and Laurasiatherian Ungulate-Like Mammals: First Morphological Evidence from the Paleocene of Morocco
Source: PLoS One. 2016 Jul 6;11(7):e0157556. doi: 10.1371/journal.pone.0157556 (PMC4934866; doi:10.1371/journal.pone.0157556)
Supplement: S4 Table — Ambiguous synapomorphies: (a) ACCTRAN optimization; (d) DELTRAN optimization. (DOC) [file pone.0157556.s006.doc]

S4 Table. Synapomorphies of *Abdounodus* and the Paenungulata (Fig 11, node 28). Ambiguous synapomorphies: (a) ACCTRAN optimization; (d) DELTRAN optimization. * denotes a non-homoplastic synapomorphy.

| Character state | RI | Description | Remarks |
| --- | --- | --- | --- |
| 35-1 | 50 | M_1-3_ Entocristid reduced | Convergence with lophodont Euungulates |
| 39-1 | 88 | M_1-3_ Postcristid reduced, hypoconulid and entoconid separated | Convergence with lophodont Euungulates |
| 40-2 | 36 | M_1-3_ Hypoconulid labial | Convergence with lophodont Euungulates |
| 42-1 | 27 | M_1-3_ Molar - Small postentoconulid | Convergence with lophodont Euungulates |
| 90-1 (a) | 87 | M^1-3^ Bunodont bilophodont pattern | Convergence with lophodont Euungulates |
| **98-0** | **77** | **M^1-3^ Postcingulum secondarily reduced** | ***Not in Euungulates. A*frotherian trait (*Potamogale) ?*** |
| ***107-3** | **100** | **M^1-3^ Metaconule enlarged & bulbous** | ***Not in Euungulates*** |
| **109-1** | **53** | **M^1-3^ Molar - Paraconule reduced** | ***Not in Euungulates*** |
| 110-1 | 81 | M^1-3^ postprotocrista reduced | Convergence with lophodont Euungulates |
| 111-1 | 69 | M^1-3^ Prehypocrista absent & metaloph present | Convergence with lophodont Euungulates |
| 112-1 | 87 | M^1-3^ Interloph present | Convergence with lophodont Euungulates |
| 140-2 | 57 | Orbit above M^1^ | Convergence with lophodont Euungulates |
