# Supplementary material for: Convergence of Afrotherian and Laurasiatherian Ungulate-Like Mammals: First Morphological Evidence from the Paleocene of Morocco
Source: PLoS One. 2016 Jul 6;11(7):e0157556. doi: 10.1371/journal.pone.0157556 (PMC4934866; doi:10.1371/journal.pone.0157556)
Supplement: S5 Table — Ambiguous synapomorphies: (a) ACCTRAN optimization; (d) DELTRAN optimization. (DOC) [file pone.0157556.s007.doc]

S5 Table. Synapomorphies of Paenungulata (Fig 11, node 44). Ambiguous synapomorphies: (a) ACCTRAN optimization; (d) DELTRAN optimization. * denotes a non-homoplastic synapomorphy.

| Character state | RI | Description | Remarks |
| --- | --- | --- | --- |
| 1- 1 (a) | **83** | **I_1-3_ procumbent** | ***Not in euungulates;***  **Known in Macroscelidea** |
| 9-0 | 20 | Lower anterior diastema reduced | Not in *Ocepeia* |
| 18-1 | 37 | P_3-4_ high paraconid | Convergence with lophodont euungulates |
| 22-1 | 0 | P_4_ metaconid present and lingual | Convergence with lophodont euungulates |
| 24-2 | 54 | M_1-3_ bunodont lophodont pattern | Convergence with lophodont euungulates |
| 25-2 | 80 | M_1-3_ lophs transverse | Some lophodont euungulates: Anthracobunidae |
| 27-1 | 80 | M_1_-_3_ lingual cusp larger than labial cusps | Convergence with lophodont euungulates |
| 28-1 | 60 | M_1-3_ short trigonid and weak paracristid | Convergence with lophodont euungulates |
| 29-1 | 70 | M_1-3_ paraconid weak | Convergence with lophodont euungulates |
| 47-2 | 69 | M^3^>M^2^ | Convergence with lophodont euungulates and louisinids |
| 50-1 |  | Mandibular symphysis fused | Not in *Eritherium* & *Phosphatherium;*  Convergence with lophodont euungulates |
| 52-0 | 0 | Mental foramen anterior (below C and anterior Pm) | Reversal in Tethytheria |
| ***59-1** | **100** | **Coronoid foramen** | ***Not in euungulates*** |
| **68-1 (d)** | **50** | **C^1^ medium to small** | ***Not in euungulates, except Hyopsodus*** |
| 76-1 (d) | 41 | P^3^ protocone present | Convergence with lophodont euungulates |
| 77-1 | 77 | P^3^ metacone present | Convergence with lophodont euungulates |
| 79-1 | 30 | P^4^ parastyle medium | Convergence with lophodont euungulates |
| 82-2 |  | P^4^ metacone medium | Convergence with lophodont euungulates |
| 91-2 | 75 | M^1-3^ lophs oblique | Convergence with lophodont euungulates  Reversal in Tethytheria |
| ***107-4 & *108-2** | **100** | **Full pseudohypocone** | ***Not in euungulates*** |
| 118-1 | 88 | Protocone root enlarged | Convergence with lophodont euungulates |
| ***119-1** | **100** | **Pseudohypocone with one root** | ***Not in euungulates*** |
| 121-1 | 66 | Occlusal outline squared | Convergence with lophodont euungulates |
| 172-1 | 100 | Subarcuata fossa poorly excavated | Reversal in *Phosphatherium* ;  Character poorly known |
