# Supplementary material for: Convergence of Afrotherian and Laurasiatherian Ungulate-Like Mammals: First Morphological Evidence from the Paleocene of Morocco
Source: PLoS One. 2016 Jul 6;11(7):e0157556. doi: 10.1371/journal.pone.0157556 (PMC4934866; doi:10.1371/journal.pone.0157556)
Supplement: S6 Table — (DOC) [file pone.0157556.s008.doc]

S6 Table. Additive characters in analysed matrix of *Abdounodus hamdii*.

ÚÄÄÄÄÄÄÂÄÄÄÄÄÄÂÄÄÄÄÄÄÂÄÄÄÄÄÄÂÄÄÄÄÄÄÂÄÄÄÄÄÄÂÄÄÄÄÄÄÂÄÄÄÄÄÄÂÄÄÄÄÄÄÂÄÄÄÄÄÄ¿

³ +0³ +1³ +2³ +3³ +4³ +5³ +6³ +7³ +8³ +9³

ÚÄÄÄÄÄÄÅÄÄÄÄÄÄÅÄÄÄÄÄÄÅÄÄÄÄÄÄÅÄÄÄÄÄÄÅÄÄÄÄÄÄÅÄÄÄÄÄÄÅÄÄÄÄÄÄÅÄÄÄÄÄÄÅÄÄÄÄÄÄÅÄÄÄÄÄÄ´

³ 0³ ³ ³ ³ X ³ ³ ³ X ³ X ³ X ³ ³

³ 10³ X ³ ³ X ³ ³ ³ ³ ³ ³ ³ ³

³ 20³ ³ ³ ³ ³ ³ ³ ³ ³ ³ ³

³ 30³ ³ ³ ³ ³ X ³ ³ ³ ³ ³ X ³

³ 40³ ³ X ³ X ³ ³ X ³ ³ ³ ³ X ³ X ³

³ 50³ ³ ³ ³ ³ ³ ³ X ³ X ³ ³ ³

³ 60³ ³ X ³ X ³ ³ X ³ ³ ³ X ³ X ³ ³

³ 70³ ³ ³ ³ ³ X ³ ³ X ³ ³ ³ ³

³ 80³ ³ X ³ ³ ³ X ³ ³ X ³ ³ ³ ³

³ 90³ ³ ³ ³ ³ ³ ³ ³ X ³ ³ X ³

³ 100³ ³ ³ ³ ³ ³ X ³ ³ X ³ X ³ ³

³ 110³ ³ ³ ³ ³ ³ ³ ³ ³ ³ ³

³ 120³ ³ ³ X ³ ³ ³ ³ ³ ³ ³ ³

³ 130³ ³ ³ X ³ ³ ³ ³ ³ X ³ ³ ³

³ 140³ ³ X ³ ³ X ³ ³ ³ ³ ³ ³ X ³

³ 150³ ³ ³ ³ ³ ³ ³ ³ ³ ³ ³

³ 160³ ³ ³ ³ X ³ X ³ ³ ³ ³ ³ ³

³ 170³ ³ ³ X ³ ³ ³ ³ ³ ³ ³ ³

³ 180³ X ³ X ³ ³ ³ ³ ³ ³ ³ ³ ³

ÀÄÄÄÄÄÄÁÄÄÄÄÄÄÁÄÄÄÄÄÄÁÄÄÄÄÄÄÁÄÄÄÄÄÄÁÄÄÄÄÄÄÁÄÄÄÄÄÄÁÄÄÄÄÄÄÁÄÄÄÄÄÄÁÄÄÄÄÄÄÁÄÄÄÄÄÄÙ

List of ordered characters (numbering from 0): 3, 6, 7, 8, 10, 12, 34, 39, 41, 42, 44, 50, 51, 56, 57, 58, 61, 62, 64, 67, 68, 74, 76, 81, 84, 86, 97, 99, 104, 105, 107, 108, 109, 122, 132, 137, 141, 143, 149, 163, 164, 172, 180, 181.
