# Supplementary material for: Convergence of Afrotherian and Laurasiatherian Ungulate-Like Mammals: First Morphological Evidence from the Paleocene of Morocco
Source: PLoS One. 2016 Jul 6;11(7):e0157556. doi: 10.1371/journal.pone.0157556 (PMC4934866; doi:10.1371/journal.pone.0157556)
Supplement: S1 Text — (DOC) [file pone.0157556.s009.doc]

First fossil evidence for convergence of African and Laurasian ungulate-like mammals

Emmanuel Gheerbrant, Andrea Filippo and Arnaud Schmitt

**SUPPORTING INFORMATION**

**S1 Text. Changes (additions and corrections) made in the matrix of Gheerbrant *et al. (*2014) for the analysis of the relationships of *Abdounodus*. The resulting new and updated matrix analysed here is given in S2 Text.**

***Remark: Characters are numbered from 0 to 183.***

**Dental features**

0 – Character modified, restricted to the development of incisors.

0 – Hyracoidea state: ?. The relative size of *Seggeurius* lower incisors is unknown.

1 – Character modified, restricted to orientation of incisors (vertical or proclive).

0 & 1 – Anthracobunidae state: 0-0 lower incisors small and simple, and 1-0 subvertical.

8 – Sirenia state: C/1: uncertain (one or two roots). Hyracoidea state: 8-1 C/1: 1 root (*Seggeurius*).

9 – Character modified (states 2 and 3)

10 – P/1 development recoded with distinction of states 10-0 “well developed” and 10-1 “small but more or less premolarized”.

10 – *Phosphatherium* state: 10-3 P/1 “absent” following Gheerbrant *et al. (*2012).

11 – Hyracoidea state: number of roots of P/1 recoded polymorphic (0,1); P/1 is developed and biradicular in *Microhyrax* (Tabuce *et al.*, 2007), but small and uniradicular in *Seggeurius* (Benoit *et al.,* 2015).

23 and 90 – Lophodonty coded non – additive, i.e. transformation of states 0🡪2 possible in Perissodactyla and Louisinidae.

30 – Embrithopoda state: 31-1 M/1– 3: premetacristid present as seen in *Palaeoamasia*.

34 – *Teilhardimys* and Macroscelidea states: (0) Hypolophid absent.

39 – Character modified, simplified with only three states.

40 – Hyracoidea state: 40-2 M/1 – 3: hypoconulid labial, as seen in *Seggeurius*.

50 – Hyacoidea state: 50-1 mandibular symphysis fused, as seen in *Seggeurius* (Benoit *et al.*, 2015).

57 – *Ocepeia* state: 57-2 mandibular condyle very high in (>= one molar length). *Abdounodus* state: 57-1 less high than in *Ocepeia*.

58 – Mandibular condyle: (0) it is cylindrical and elongated transversely in *Ocepeia, Seggeurius* (Hyracoidea), *Phosphatherium, Numidotherium, Prorastomus* (Sirenia) and *Arsinoitherium* (Embrithopoda)*.*

65 – Embrithopoda state: ?. Upper incisors of embrithopods are known only in *Arsinoitherium* that is specialized in the order.

68 – *Eritherium* state: 68-1 upper canine small following Gheerbrant *et al.* (2012), in agreement with a small lower canine.

69 – Sirenia state: 69-1 C1/: one root.

79 – New feature; P4/ and P3/: 79-0 parastyle large, 79-1 medium or 79-2 well developed.

84 – New feature; P4/ protocone development : 84-0 absent, 84-1 present – medium, 84-2 large.

88 – Anthracobunidae state: 88-0 postprotocrista present on P3 – 4/. *Phosphatherium* state: 88-1 postprotocrista absent on P3 – 4/ (Gheerbrant *et al.*, 2005).

93 – Parastyle states coded non additive, as it seems to enlarge in selenodont taxa such as hyracoids.

98 – *Ocepeia* state: 98-1 upper molar postingulum present.

103 – Hyracoidea state: centrocrista shape polymorphic; it is (103-1) poorly dilambdodont in *Helioseus* (formerly identified *Seggeurius*, see Tabuce *et al.,* 2007: Fig. 2d – e), and (103-2) strongly dilambdodont in *Seggeurius*.

104 – *Ocepeia* state: 104-0 postmetacrista well developed and transverse.

106 – *Ocepeia* state: M3/ metacone smaller than paracone *contra* Gheerbrant *et al. (*2014).

107, 109 – paraconule and metaconule separated as two characters; *Ocepeia* state*:* conules well developed (108 – 2, 110 – 0).

111 – Character 107 of Gheerbrant *et al. (*2014) modified; in paenungulates, the prehypocrista” is not homologous with the anterior crest of the hypocone seen in euungulates (Perissodactyla) and in macroscelids, and louisinids; it corresponds to a neoformed crest issued from the pseudohypocone (enlarged metaconule).

113 – Anthracobunidae state: 113-1 distocrista absent.

114 – Anthracobunidae state: 114-0 postentoconule absent. However a similar cusp is present in *Indobune* (= Cambaytheriidae in Cooper *et al.*, 2015); it might not be homologous because it is lingual to the hypocone and not posterior and linked to postcingulum – distocrista.

119 – “M3 hypocone root” renamed “postero-lingual root under hypocone or pseudohypocone”, because of the non-homology of the hypocone and pseudohypocone (metaconule) evidenced in Paenungulata and Perissodactyla.

**Skull features**

For skull characters of anthracobunids (mainly *Anthracobune*) we followed the description and study of Cooper *et al. (*2015).

123 – Rostrum elongation: *Radinskya* state: 123-0; the relative preorbital length of *Radinskya* (36% at least) was estimated according to Holbrock (2014).

134 – Character modified (frontal and maxillary contact anterior to orbit). The definition of this character was refined for more details. It is equivalent to part of character 10 of Rose *et al. (*2014). The character is inapplicable when the lacrimal is absent.

134 – State 134-0: *Zalambdalestes*, *Kulbeckia,* Phenacodonta (*contra* Holbrook, 2014). State 134-1: *Potamogale* (*contra* Gheerbrant *et al.,* 2014), Anthracobunidae (Rose *et al.*, 2014), Hyracoidea (*Dimaitherium*).

135 – New feature: Naso – frontal sutures (Holbrook; 2014; Rose *et al.*, 2014): 135-0 convex posteriorly (nasal intrudes distally in frontals); 135-1 Naso – frontal sutures straight transversely.

137 – Character modified: additional states are included following Rose *et al. (*2014): 137-0 lacrimal facial process large contacting nasal; 137-1 large or moderate, not contacting nasal; (2) small, reduced along orbital rim; (3) absent.

138 – *Radinskya* state: 138-1 Small lacrimal tubercle present (Holbrook, 2014).

140 – Character modified: Relative position of the orbit was defined more accurately (6 states instead 5).

143 – Character modified: states 143-0 and 143-1 changed; an infraorbial foramen anterior to the last premolar is considered to be in the primitive eutherian condition by comparison to many Cretaceous taxa.

147 – State 147-1 (large tuber maxillae) in *Radinskya*, Phenacodonta, and *Cambaytherium* according to Holbrook (2014) and Rose *et al. (*2014).

166 – Periotic mastoidy/amastoidy: Following Holbrook (2014) exposure of pars mastoidea was distinguished with two states: state (0): posterior mastoidy: large posterior exposure of pars mastoidea between exoccipital and squamosal ; state 166-1: small lateral mastoid exposure between exoccipital and squamosal; state 166-2 is pars mastoidea of periotic not present between squamosal and exoccipital.

173 – New feature: Preglenoid process (Holbrook, 2014; Rose *et al.,* 2014): 173-0 absent; 173-1 present. This character is in state 173-0 in most of our analyzed taxa, except *Hyopsodus*; the state 173-1 occurs in some perissodactyls not analyzed here (Rose *et al.,* 2014).

174 – Anthracobunidae: The postglenoid foramen is described absent in *A. pinfoldi* by Cooper *et al. (*2014), but the state is coded unknown in their matrix. We coded it unknown.

**Petrosal and Inner ear features**

**C**haracters states of inner ear for paenungulates were completed based on recent studies of Benoit *et al.* (2013a,b,c). Sirenia: some petrosal features for this order were re-coded according to the description of the new sirenian from the early/middle Eocene of Chambi, Tunisia (Benoit *et al.*, 2013); e.g. character 170: Fenestra vestibuli “strongly oval” (round in *Prorastomus*) ; character 171: postero-medial position of the fenestra cochleae.

164 – Sagittal crest: one intermediate state added to this character following Cooper *et al. (*2015): 164-0 Sagittal crest present all along top of skull (joining temporal crests); 164-1 sagittal_crest reduced to back of skull; 164-2 Sagittal crest weak or absent.

165 – Nuchal crest: Hyracoidea state: 165-0 – present (*Seggeurius*, Benoit *et al.,* 2015).

172 – Fossa subarcuata: Hyracoidea state: 172-1 – shallow (*Seggeurius*, Benoit *et al.,* 2015).

180 – Character modified. To take into account the morphology of the sirenian from Chambi (CBI 1-542; Benoit *et al.,* 2013) we added state 180-2: absence of secondary bony lamina.

181 – Number of turns of the cochlea: Hyracoidea state (*Seggeurius*, Benoit *et al.*, 2015): 181-1 –between 1.5 and 2.

182 **–** New character: secondary crus commune : 182-1 present; 182-1 absent.

183 **–** New character: shape of cochlear canal: 183-0 planispiral (cochlear ratio<0.6); 183-1 conic (cochlear ratio>0.6).

183 **–** Hyracoidea state: 183-0 (*Seggeurius*, Benoit *et al.*, 2015)**.**

**References.**

Benoit, J., Adnet, A., Ben, Haj, Ali, M., Essid, E.M., Marivaux, L., Merzeraud, G., Merigeaud, S., Vianey –Liaud, M., Tabuce, R. (2013a). Cranial remain of a stem sea – cow (Mammalia, Sirenia): evidence for a freshwater origin in Africa. *Plos One*, 8, p. e54307.

Benoit, J., Merigeaud S., Tabuce R. (2013b). Homoplasy in the ear region of Tethytheria and the systematic position of Embrithopoda (Mammalia, Afrotheria). Geobios 46, 357 – 370.

Benoit, J., Orliac, M., Tabuce, R., (2013c). The petrosal of *Chambius* (Macroscelidea, Afrotheria) from the Eocene of Djebel Chambi (Tunisia). *Journal of Systematic Paleontology,* 11, 8, 907-923.

Benoit, J. , Crochet, J . - Y ., Mahboubi, M. , Jaeger, J. - J. , Bensalah, M. , Adaci, M., Tabuce, R. (2015). New material of *Seggeurius amourensis* (Paenungulata, Hyracoidea) including a partial skull with intact basicranium. *Journal of Vertebrate Paleontology* 36 (2), in press. DOI: 10.1080/02724634.2015.1034358.

Cooper, L.N., Seiffert, E.R., Clementz, M., Madar, S.M., Bajpai, S., Hussain, S.T., Thewissen, J. G. M. (2014). Anthracobunids from the Middle Eocene of India and Pakistan are stem Perissodactyls. *PLoS ONE* 9(10): e109232. doi:10.1371/journal.pone.0109232

Gheerbrant, E., Sudre, J., Tassy, P., Amaghzaz, M., Bouya., B., & Iarochene, M. (2005a). Nouvelles données sur *Phosphatherium escuilliei* (Mammalia, Proboscidea) de l’Eocène inférieur du Maroc, apports à la phylogéenie des Proboscidea et des ongulés lophodontes. *Geodiversitas* 27, 239–333.

Gheerbrant, E., Bouya B. & Amaghzaz, M. (2012). Dental and cranial anatomy of *Eritherium azzouzorum* from the Paleocene of Morocco, earliest known proboscidean mammal. *Palaeontographica* Abt A, 297, 151–183.

Gheerbrant, E, Amaghzaz M., Bouya B., Goussard F., Letenneur C. (2014). *Ocepeia* (Middle Paleocene of Morocco): the oldest skull of an afrotherian mammal. PLoS ONE 9 (2), e89739.

Holbrook, L. T. (2014). On the skull of *Radinskya* (Mammalia) and its phylogenetic position. *Journal of Vertebrate Paleontology*, *34*(5), 1203-1215.

Rose, K. D., Holbrook, L. T., Rana, R. S., Kumar, K., Jones, K. E., Ahrens, H. E., Missiaen, P., Sahni, A., Smith, T. (2014). Early Eocene fossils suggest that the mammalian order Perissodactyla originated in India. *Nature Communications* 5 (5570), 1-9.

Tabuce, R., Marivaux, L., Adaci, M., Bensalah, M., Hartenberger, J. L., Mahboubi, M., Mebrouk, F., Tafforeau, P., Jaeger, J.-J. (2007). Early Tertiary mammals from North Africa reinforce the molecular afrotheria clade. *Proceedings of the Royal Society of London, B* 274, 1159–1166.

Table S1-1- Correspondence of the character numbers of the phylogenetic matrix analyzed here (numbering 1-183) with characters numbers used in Gheerbrant *et al. (*2014). (*) character modified; (n) = new character. Characters 41 and 96 (numbering 1-175) of Gheerbrant *et al. (*2014) are not retained here.

| This work, numbering  0-183 | Gheerbrant *et al. (*2014) numbering  1-175 | Gheerbrant *et al. (*2014) numbering  0-174 |
| --- | --- | --- |
| 0 | 1(*) | 0 (*) |
| 1 | 2 | 1 |
| 2 | 3 | 2 |
| 3 | 4 | 3 |
| 4 | 5 | 4 |
| 5 | 6 | 5 |
| 6 | 7 | 6 |
| 7 | 8 | 7 |
| 8 | 9 | 8 |
| 9 | 10 (*) | 9 (*) |
| 10 | 11 | 10 |
| 11 | 12 | 11 |
| 12 | 13 | 12 |
| 13 | 14 | 13 |
| 14 | 15 | 14 |
| 15 | 16 | 15 |
| 16 | 17 | 16 |
| 17 | 18 | 17 |
| 18 | 19 | 18 |
| 19 | 20 | 19 |
| 20 | 21 | 20 |
| 21 | 22 | 21 |
| 22 | 23 | 22 |
| 23 | 24 | 23 |
| 24 | 25 | 24 |
| 25 | 26 | 25 |
| 26 | 27 | 26 |
| 27 | 28 | 27 |
| 28 | 29 | 28 |
| 29 | 30 | 29 |
| 30 | 31 | 30 |
| 31 | 32 | 31 |
| 32 | 33 | 32 |
| 33 | 34 | 33 |
| 34 | 35 | 34 |
| 35 | 36 | 35 |
| 36 | 37 | 36 |
| 37 | 38 | 37 |
| 38 | 39 | 38 |
| 39 | 40 (*) | 39 (*) |
| 40 | 42 | 41 |
| 41 | 43 | 42 |
| 42 | 44 | 43 |
| 43 | 45 | 44 |
| 44 | 46 | 45 |
| 45 | 47 | 46 |
| 46 | 48 | 47 |
| 47 | 49 | 48 |
| 48 | 50 | 49 |
| 49 | 52 | 50 |
| 50 | 52 | 52 |
| 51 | 53 | 52 |
| 52 | 54 | 53 |
| 53 | 55 | 54 |
| 54 | 56 | 55 |
| 55 | 57 | 56 |
| 56 | 58 | 57 |
| 57 | 59 | 58 |
| 58 | 60 | 59 |
| 59 | 61 | 60 |
| 60 | 62 | 61 |
| 61 | 63 | 62 |
| 62 | 64 | 63 |
| 63 | 65 | 64 |
| 64 | 66 | 65 |
| 65 | 67 | 66 |
| 66 | 68 | 67 |
| 67 | 69 | 68 |
| 68 | 70 | 69 |
| 69 | 71 | 70 |
| 70 | 72 | 71 |
| 71 | 73 | 72 |
| 72 | 74 | 73 |
| 73 | 75 | 74 |
| 74 | 76 | 75 |
| 75 | 77 | 76 |
| 76 | 78 | 77 |
| 77 | 79 | 78 |
| 78 | 80 | 79 |
| 79(n) | -- | -- |
| 80 | 81 | 80 |
| 81 | 82 | 81 |
| 82 | 83 | 82 |
| 83 | 84 | 83 |
| 84(n) | -- | -- |
| 85 (n) | -- | -- |
| 86 | 85 | 84 |
| 87 | 86 | 85 |
| 88 | 87 | 86 |
| 89 | 88 | 87 |
| 90 | 89 | 88 |
| 91 | 90 | 89 |
| 92 | 91 | 90 |
| 93 | 92 | 91 |
| 94 | 93 | 92 |
| 95 | 94 | 93 |
| 96 | 95 | 94 |
| 97 | 97 (*) | 96 (*) |
| 98 (n) | -- | -- |
| 99 | 98 (*) | 97 (*) |
| 100 | -- | -- |
| 101 | 99 | 98 |
| 102 | 100 | 99 |
| 103 | 101 (*) | 100 (*) |
| 104 | 102 | 101 |
| 105 | 103 | 102 |
| 106 | 105 (*) | 104 (*) |
| 107 (n) | -- (105 in part) | -- (104in part) |
| 108 (n) | - - | - - |
| 109 | -- (105 in part) | -- (104 in part) |
| 110 | 106 | 105 |
| 111 | 107 (*) | 106 (*) |
| 112 | 108 | 107 |
| 113 | 109 | 108 |
| 114 | 110 | 109 |
| 115 | 111 | 110 |
| 116 | 112 | 111 |
| 117 | 113 | 112 |
| 118 | 114 | 113 |
| 119 | 115 (*) | 114(*) |
| 120 | 116 | 115 |
| 121 | 117 | 116 |
| 122 | 118 | 117 |
| 123 | 119 | 118 |
| 124 | 120 | 119 |
| 125 | 121 | 120 |
| 126 | 122 | 121 |
| 127 | 123 | 122 |
| 128 | 124 | 123 |
| 129 | 125 | 124 |
| 130 | 126 | 125 |
| 131 | 127 | 126 |
| 132 | 128 | 127 |
| 133 | 129 | 128 |
| 134 | 130 (*) | 129 (*) |
| 135 (n) | -- | -- |
| 136 | 131 | 130 |
| 137 | 132 (*) | 131 (*) |
| 138 | 133 | 132 |
| 139 | 134 | 133 |
| 140 | 135(*) | 134 (*) |
| 141 | 136 | 135 |
| 142 | 137 | 136 |
| 143 | 138 (*) | 137 (*) |
| 144 | 139 | 138 |
| 145 | 140 | 139 |
| 146 | 141 | 140 |
| 147 | 142 | 141 |
| 148 | 143 | 142 |
| 149 | 144 | 143 |
| 150 | 145 | 144 |
| 151 | 146 | 145 |
| 152 | 147 | 146 |
| 153 | 148 | 147 |
| 154 | 149 | 148 |
| 155 | 150 | 149 |
| 156 | 151 | 150 |
| 157 | 152 | 151 |
| 158 | 153 | 152 |
| 159 | 154 | 153 |
| 160 | 155 | 154 |
| 161 | 156 | 155 |
| 162 | 157 | 156 |
| 163 | 158 | 157 |
| 164 | 159(*) | 158 (*) |
| 165 | 160 | 159 |
| 166 | 161 (*) | 160 (*) |
| 167 | 162 | 161 |
| 168 | 163 | 162 |
| 169 | 164 | 163 |
| 170 | 165 | 164 |
| 171 | 166 | 165 |
| 172 | 167 | 166 |
| 173 (n) | -- | -- |
| 174 | 168 | 167 |
| 175 | 169 | 168 |
| 176 | 170 | 169 |
| 177 | 171 | 170 |
| 178 | 172 | 171 |
| 179 | 173 | 172 |
| 180 | 174 | 173 |
| 181 | 175 (*) | 174 (*) |
| 182 (n) | -- | -- |
| 183 (n) | -- | -- |
