# Supplementary material for: Convergence of Afrotherian and Laurasiatherian Ungulate-Like Mammals: First Morphological Evidence from the Paleocene of Morocco
Source: PLoS One. 2016 Jul 6;11(7):e0157556. doi: 10.1371/journal.pone.0157556 (PMC4934866; doi:10.1371/journal.pone.0157556)
Supplement: S2 Text — (PDF) [file pone.0157556.s010.pdf]

Convergence of Afrotherian and Laurasiatherian Ungulate-like Mammals: first Morphological Evidence from the Paleocene of Morocco

Emmanuel Gheerbrant, Andrea Filippo and Arnaud Schmitt

SUPPORTING INFORMATION

S2 Text. Phylogenetic analysis of *Abdounodus*

- Part I. Studied characters of *Abdounodus hamdii*
- Part II. Character matrix for *Abdounodus hamdii*, characters summary list, taxa analyzed
- Part III. Cladistic analysis with TNT, method, cladograms, and characters at nodes.

Part I

Studied characters of *Abdounodus hamdii*

The characters list (K0 to K183) of our matrix corresponds to that of Gheerbrant *et al.* (2014) with several corrections, deletions and additions that are detailed in Appendix S1.

Lower Dentition

K0 Lower incisors development and shape
(0) Lower incisors small, peg like, (1) spatulate Lower incisors, (2) higher or enlarged.
K1 Lower incisors lower incisors
(0) subvertical lower incisors, (1) enlarged and procumbent.
K2 I/1
(0) I/1present, (1) I/1 absent.
K3 I/1 enlargement
(0) I/1 and I/2 of similar size, (1) I/1 slightly larger than I/2, (2) I/1 very enlarged, (3) I/1 hypsodont.
K4 I/2
(0) I/2 present, (1) absent.
K5 I/2 enlargement
(0) I/1 and I/2 of similar size, (1) I/2 larger than I/1.
K6 I/3
(0) I/3 present, (1) well developed, (1) I/3 very small and labial to C/1, (2) I/3 lost.
K7 Lower canine

(0) Lower canine very large, more than two times higher than molars , (1) Large, higher than molars (no more than two times), (2) medium, as high than molars, (3) small, lower and smaller than molars, (4) very small, smaller and lower than P/2, (5) absent.

K8 C/1 Number of roots

(0) C/1 biradicular, (1) C/1 uniradicular.

K9 Lower anterior diastema

(0) diastema absent or very short present, (1) diastema well distinct long (in relation to loss of 2 teeth, (2) diastema C/1 and P/1 or P/1 and P/2), (3) very long (and in relation to loss of at least two teeth).

K10 P/1 development

(0) P/1 well developed, (1) P/1 small but more or less premolarized, (2) P/1 small and simple (basically conical), (3) P/1 absent.

K11 P/1: Number of roots

(0) P/1 biradicular, (1) P/1 uniradicular.

K12 P/2 development

(0) P/2 well developed, (1) P/2 small, but premolariform, (2) P/2 small and simple (basically conical), (3) P/2 absent.

K13 P/2: Number of roots

(0) P/2 biradicular, (1) P/2 uniradicular.

K14 P/3

(0) biradicular and large, (1) uniradicular and smaller crown.

K15 P/3: Metaconid

(0) P/3: Metaconid absent or weak, (1) P/3: Metaconid present.

K16 P/3: Paraconid

(0) P/3: Paraconid present, (1) P/3: Paraconid absent or weak.

K17 P/3: Talonid

(0) P/3: Talonid reduced and simple (mostly cutting or piercing), (1) P/3: Talonid developed and molariform.

K18 P/3-4: Paraconid location

(0) P/3-4: Paraconid low, (1) P/3-4: Paraconid high.

K19 P/4

(0) biradicular and large, (1) one root and smaller crown.

K20 P/4: Paraconid development

(0) Paraconid present, (1) Paraconid absent or weak.

K21 P/4: Talonid

(0) P/4: Talonid reduced (small) and simple (mostly cutting), (1) P/4: Talonid enlarged and molariform .

K22 P/4: Metaconid

(0) P/4: Metaconid weak and distal with respect to protoconid, (1) P/4: Metaconid present and lingual .

K23 Lower premolars (at least P/4): Labial cingulum (ectocingulid)

(0) Lower premolars (at least P/4): Labial cingulum absent, (1) Lower premolars (at least P/4): Labial cingulum present.

K24 M/1-3: Molar pattern

(0) M/1-3: Molar pattern tribosphenic with high trigonid + sharp cusps, (1) M/1-3: Bunodont pattern with low trigonid, (2) M/1-3: Bunodont-lophodont pattern, (3) M/1-3: True lophodont pattern, (4) M/1-3: Bunolophodont pattern.

K25 M/1-3: Lophs

(0) M/1-3: No lophs, M/1-3: Lophs oblique M/1-3: Lophs transverse.

K26 M/1-3

(0) Crown uniformly brachyodont, (1) unilateral lingual and/or labial hypsodonty, (2) crown uniformly hypsodont.

K27 M/1-3: Labial cusps (protoconid-hypoconid) vs lingual cusps (metaconid-entoconid)
(0) M/1-3: Labial cusps larger than lingual cusps, (1) M/1-3: Lingual cusps larger than labial cusps (or cusps subequal).
K28 M/1-3:Trigonid elongation and paracristid
(0) M/1-3: Trigonid elongated with well differentiated paracristid, (1) M/1-3: Trigonid short, with paracristid weakened.
K29 M/1-3: Paraconid
(0) M/1-3: Paraconid present, (1) M/1-3: Paraconid weak or absent.
K30 M/1-3: Premetacristid
(0) M/1-3: Premetacristid weak or absent, (1) M/1-3: Premetacristid developed.
K31 M/1-3: Posmetacristid
(0) M/1-3: Posmetacristid reduced or absent, (1) M/1-3: Postmetacristid well developed, (2) M/1-3: Postmetacristid more or less inflated as a metastylid.
K32 M/1-3: Protocristid
(0) M/1-3: Protocristid depressed by a deep median notch, (1) M/1-3: Protocristid lophid-like, with no strong notch .
K33 M/1-3: Hypolophid
(0) M/1-3: Hypolophid absent, (1) M/1-3: Hypolophid present.
K34 M/1-3: Hypolophid
(0) M/1-3: Hypolophid absent or incomplete, (1) M/1-3: Hypolophid depressed by a deep median notch, (2) M/1-3: Hypolophid lophid-like.
K35 M/1-3: Entocristid
(0) M/1-3: Entocristid present, (1) M/1-3: Entocristid reduced to absent.
K36 M/1-3: Entoconulid
(0) M/1-3: Entoconulid absent, (1) M/1-3: Entoconulid present.
K37 M/1-3: Mesoconid
(0) M/1-3: Mesoconid absent, (1) M/1-3: Mesoconid present.
K38 M/1-3: Cristid obliqua
(0) M/1-3: Cristid obliqua strongly oblique, joining the trigonid in its lingual part, (1) M/1-3: Cristid obliqua joining the trigonid at its mid-width, (2) M/1-3: Cristid obliqua joining the trigonid in its labial part.
K39 M/1-3 Postcristid development and link to hypoconulid
(0) M/1-3: Postcristid developed, linking hypoconulid to other cusps, (1) M/1-3: Hypoconulid and entoconid separated by a narrow notch, (2) M/1-3: Postcristid absent, hypoconulid cingular like.
K40 M/1-2: Hypoconulid
(0) M/1-2: Hypoconulid median transversely, (1) M/1-2: Hypoconulid lingual, (2) M/1-2: Hypoconulid labial.
K41 M/1-2: Postentoconulid
(0) M/1-2: Postentoconulid absent, (1) M/1-2: Postentoconulid present, but weak, (2) M/1-2: Postentoconulid well developed.
K42 M/3: Postentoconulid
(0) M/3: Postentoconulid absent, (1) M/3: Postentoconulid present, but weak, (2) M/3: Postentoconulid well developed.
K43 M/1-3: Ectocingulid and/or ectostylid
(0) M/1-3: Ectocingulid and/or ectostylid weak or absent, (1) M/1-3: Ectocingulid present.
K44 M/1: Trigonid/talonid relative width
(0) M/1:Trigonid wider than the talonid, (1) M/1: Trigonid subequal to the talonid, (2) M/1: Trigonid narrower than the talonid.
K45 M/2: Trigonid/talonid relative width
(0) M/2: Trigonid wider than the talonid, (1) M/2: Trigonid subequal to the talonid, (2) M/2: Trigonid narrower than the talonid.
K46 M/3 trigonid/talonid relative width

(0) M/3: Trigonid wider than the talonid, (1) M/3: Trigonid subequal to the talonid, (2) M/3: Trigonid narrower than the talonid.
K47 M/3 relative size
(0) M/3 of similar size to M/2, or slightly larger, (1) M/3 smaller than M/2, (2) M/3 significantly larger than M/2, which is larger than M/1.
K48 M/3
(0) two roots, (1) two roots, but roots fused in higher part, (2) one root.

Dentary

K49 Dentary: Extension of mandibular symphysis
(0) Mand symphysis short, extended to P/2 or more anterior, (1) Mandibular symphysis moderately long: extended between P/2 and P/3, (2) Mand symphysis long, extended to P/3 or posterior.
K50 Dentary: Fusion of the mandibular symphysis
(0) Mandibular symphysis unfused (synchondrosis), (1) Mandibular symphysis fused.
K51 Dentary: Anteriormost mental foramen
(0) Anteriormost mental foramen below P/1 or more anterior, (1) below P/2, (2) below P/3, (3) posterior to P/3.
K52 Dentary:Posteriormost mental foramen
(0) below canine and anterior premolars, (1) below penultimate premolar, (2) below ultimate premolar, (3) at ultimate premolar and first molar junction, or more posterior.
K53 Dentary: Horizontal ramus (body)
(0) Dentary: Horizontal ramus (body) low, (1) Dentary: Horizontal ramus (body) high.
K54 Dentary: Position of the coronoid apophysis
(0) Coronoid apophysis rising at level of M/3, or more distally, (1) Coronoid apophysis rising anteriorly to M/3.
K55 Dentary: Orientation of coronoid apophysis
(0) Dentary: Anterior margin vertical or posteriorly canted, (1) Dentary: Anterior margin anteriorly canted .
K56 Dentary: Development of the coronoid apophysis
(0) coronoid apophysis very high above tooth row (as high or higher than M/3-P/4 length), (1) coronoid apophysis moderately high above tooth row, (2) coronoid apophysis strongly reduced to absent.
K57 Dentary: Mandibular condyle position
(0) At level or low above the dental row, (1) Moderately high above the dental row (one molar length), (2) Very high above the tooth row (>one molar length).
K58 Mandibular condyle shape
(0) ovoid (or plate-like), (1) transversely elongate and cylindrical, (2) antero-posteriorly elongate.
K59 Dentary: Coronoid foramen
(0) Coronoid foramen absent, (1) Coronoid foramen present.
K60 Dentary: Angular process
(0) thin stick-like or hook-like distally protruding angular process, (1) broad and rounded distally projecting angular process, (2) Mandibular angle broad-and round without angular process.
K61 Dentary: Anterior coronoid (= retromolar) fossa
(0) absence of retromolar fossa, (1) retromolar fossa present, (2) retromolar fossa enlarged.

Upper Dentition

K62 Number of upper incisors
(0) three, (1) two, (2) one, (3) none.
K63 Relative size of upper incisors
(0) small, (1) large.
K64 Relative size of I1/
(0) not enlarged (= I2/), (1) I1/>I2/, (2) I1/ greatly enlarged (I1/>>I2/. I1/ tusk like).
K65 Relative size of I2/
(0) I2/ not enlarged (similar to I1/), (1) I2/>>I1/.
K66 Relative size of I3/
(0) I3/ similar in size to I2/ and I1/, (1) I3/ very small, much smaller than I2/ and I1/, (2) I3/ larger than I2/ and I1/.
K67 Upper diastema
(0) small or absent, (1) developed but short elongated (I2/-P2/).
K68 C1/
(0) large (>> premolars), (1) medium to small (= or < premolars and incisors), (2) absent.
K69 C1/: number of roots
(0) two roots, (1) one root.
K70 P1/
(0) P1/: present, (1) P1/: absent.
K71 P1/: Number of roots
(0) P1/: Two roots, (1) P1/: One root.
K72 P2/
(0) P2/: Present, (1) P2/: Absent.
K73 P2/: Number of roots
(0) P2/: Two roots, (1) P2/: Three roots, (2) P2/: One root.
K74 P2/: Protocone
(0) P2/: Protocone absent, crown narrow transversely, (1) P2/: Protocone present, crown slightly developed transversely, (2) P2/: Protocone large, crown as long as wide.
K75 P3/: Number of roots
(0) P3/: Two roots, (1) P3/: Three roots, (2) P3/: one root.
K76 P3/: Protocone
(0) P3/: Protocone absent or reduced, (1) P3/: Protocone present, (2) P3/: Protocone well developed, crown at least as wide as long.
K77 P3/: Metacone
(0) P3/: Metacone absent, (1) P3/: Metacone present and small, (2) P3/: Metacone medium to large, but still closely connate to paracone, (3) P3/: Metacone large and well separated from paracone.
K78 P3/: Conules
(0) absent, (1) present.
K79 P4/ (and P3/): parastyle
(0) large, (1) medium, (2) small or absent.
K80 P4/: Conules
(0) absent, (1) present.
K81 P4/: Number of roots
(0) P4/: Two roots, (1) P4/: Three roots, (2) P4/: Four roots, (3) P4/: One root.
K82 P4: metacone
(0) absent, (1) present, small, 2 Metacone medium (smaller than paracone), (3) Metacone large, close in size to paracone.
K83 P4/: metacone
(0) Metacone conate to paracone, (1) Metacone well separated from paracone.

K84 P4/: protocone
(0) P4/: protocone absent or weak, (1) P4/: protocone present, medium, (2) P4/: protocone large (occlusal outline at least as wide as long).
K85 P4/: lingual cingulum
(0) absent, (1) present .
K86 P4/: Hypocone
(0) P4/: Hypocone absent, (1) P4/: Hypocone present, small, (2) P4/: Hypocone present, large.
K87 P4/: Lophs
(0) P4/: No loph, (1) P4/: At least one loph present.
K88 P4/ (and P3/): postprotocrista
(0) P4/ (and P3/): Postprotocrista distinct, (1) P4/ (and P3/): Postprotocrista absent or weak.
K89 M1-3/ bunodonty
(0) Morphology more or less puncturing with high and pointed cusps, (1) Morphology bunodont with low and inflated crown and cusps.
K90 M1-3/: Lophodonty
(0) Strictly bunodont pattern without loph, (1) Bunodont bilophodont pattern, with weak meta- and protololophs, (2) Bunodont bilophodont pattern with developed meta and protoloph, (3) True lophodont pattern, 4 Bunolophodont pattern.
K91 M1-3/: Lophs
(0) No loph, (1) Lophs transverse, (1) Lophs oblique.
K92 M-13/ parastyle position
(0) labial to paracone, (1) shifted mesially to paracone.
K93 M1-3/ parastyle development
(0) large, (1) small, (2) absent.
K94 M1-3/: Ectocingulum
(0) Ectocingulum reduced, or thin and isolated, (1) Ectocingulum inflated and continuous with mesial and distal cingula.
K95 M1-2/: Stylar shelf development
(0) Stylar shelf wide (5(0) -25 % molar width), (1) Stylar shelf very wide, enlarged (>5(0) % molar width), (2) Stylar shelf reduced (< 25% molar width), (3) Stylar shelf absent.
K96 M1-3/: Pericone
(0) M1-3/: Pericone absent, (1) M1-3/: Pericone absent present.
K97 M1-3/ true hypocone (cingulum derived)
(0) absent or weak, (1) small, crestiform, (2) well inflated, (2) subegal protocone.
K98 Postcingulum
(0) absent or very small, (1) present (joined or not to distocrista).
K99 M1-3/ mesostyle development
(0) absent or weak, (1) present and inflated, (2) enlarged.
K10(0) Mesostyle longitudinal position
(0) more or less in between paracone and metacone, (1) distinctly closer to metacone (alignement with metaloph).
K1(0) 1 M1-3/ mesostyle transverse position
(0) distant labially from paracone and metacone, (1) close to paracone and metacone.
K102 M1-3/ centrocrista
(0) present, (1) absent.
K103 M1-3/ Centrocrista shape
(0) rectodont, not linked to mesostyle moderatly, (1) dilambdodont and linked to a close mesostyle, (2) strongly dilambdodont (V shaped centrocr.) & linked to mesostyle, (3) hyperdilambdodont (para- & metac lingual) & linked to mesostyle.
K104 M1-3/: Postmetacrista
(0) Postmetacrista well developed and predominantly transverse, (1) Postmetacrista weak and predominantly longitudinal.

K105 M-13/ Preparacrista
(0) long and transverse, (1) short and longitudinal, (2) absent or weak.
K106 M3/: Metacone development
(0) Metacone smaller than paracone but well developed, (1) Metacone weak or absent on M3/, (2) Metacone as large or larger than paracone.
K107 M1-3/ metaconule development
(0) Absent to weak Small but well distinct (<paraconule), (1) Large (= or >paraconule), (2) Distinctly enlarged and bulbous (>>paraconule, but < protocone), (3) As large as protocone= pseudohypocone.
K108 M1-3/ metaconule position
(0) Labial: more labial or equal to paraconule, (1) Shifted linguallly close to protocone level, (2) Lingual, behind the protocone or more lingual: pseudohypocone.
K109 M1-3/ paraconule development
(0) present but small (crestiform), (1) absent or weak large and inflated.
K110 M1-3/ postprotocrista
(0) present, (1) absent or reduced (partly correlated with interloph development).
K111 M1-3/: "Prehypocrista" and metaloph
(0) "Prehypocrista" and metaloph absent or reduced, (1) "Prehypocrista" absent or weak, metaloph present, (2) "Prehypocrista" forms metaloph and joins metacone base, (3) "Prehypocrista" forms metaloph and joins metacone apex.
K112 M1-3/ Interloph
(0) absent, (1) present.
K113 M1-2/: Distocrista
(0) Distocrista present between postcingulum and hypocone, (1) Distocrista absent, postcingulum independant from hypocone.
K114 M1-3/: Postentoconule (hypostyle)
(0) Postentoconule (hypostyle) absent, (1) Postentoconule (hypostyle) present.
K115 M1-2/: M1/ and M2/ relative size
(0) M1/ and M2/ of similar size, (1) M1/ significantly smaller than M2/, (2) M1/ significantly larger than M2/.
K116 M2/ and M3/ relative size
(0) M2/ and M3/ of similar size, (1) M3/ smaller than M2/, (2) M3/ strongly reduced, (3) M3/ significantly larger than M2/ (molar size increase posteriorly).
K117 M1-2/ lingual root
(0) M1-2/: one simple lingual root, (1) M1-2/: Lingual root enlarged + incipiently divided (sulcus),
(2) M1-2/: At least 2 lingual roots (hypocone or pseudohypocone root).
K118 M3/: Protocone root
(0) M3/: Protocone root small and simple, (1) M3/: Protocone root enlarged (or fused with hypocone root).
K119 M3/ postero-lingual root under hypocone or pseudohypocone
(0) Postero-lingual root absent, (1) One root under hypocone or pseudohypocone, (2) 2 roots (mesial and distal) roots under hypocone or pseudohypocone.
K12(0) M1-3/: Brachyodonty/hypsodonty
(0) Crown uniformly brachyodont Unilateral labial hypsodonty, (1) Unilateral lingual hypsodonty, (2) Crown uniformly hypsodont.
K121 M1-3/: Occlusal outline
(0) M1-3/: Occlusal outline extended transversally, (1) M1-3/: Occlusal outline narrower (squared or slightly elongated) .

Skull

K122 Skull pneumatization
(0) Reduced pneumatization of skull bones, (1) Significant pneumatization (developed sinuses/diploe), (2) Extensive pneumatization.
K123 Rostrum elongation
(0) Preorbital length vs total skull length between 30% and 50%, (1) Preorbital length vs total skull length > 50%, (2) Preorbital length vs total skull length < 30%.
K124 Rostrum relative width to length: ratio of width of the snout at canine level to preorbital length
(0) Between 30% (included) and 50% < 30%, (1) Between 50% (included) and 70%, (2) Between 70% (included) and 90%, (3) > 90%.
K125 Skull: Extension ot the tooth row
(0) Upper tooth row not extended more distally than mid skull length, (1) upper tooth row very extended distally, up to the two thirds of the skull.
K126 Skull: Palatine development
(0) Palatine anteriorly extended to M1/ level, (1) Palatine short, more posterior than M1/, (2) Palatines more anterior than M1/.
K127 Skull: Major palatine foramen
(0) Within palatine, (1) Between palatine and maxilla, (2) Within maxilla, (3) Multiple small foramina, (4) Absent.
K128 Skull: Location of anterior margin of the choanae
(0) At M3/ level, (1) Posterior to M3/, (2) Anterior to M3/.
K129 Skull: Postpalatine torus
(0) present, (1) absent.
K130 Skull: Nasal cavity development
(0) Nasal cavity narrow and not high anteriorly, (1) Nasal cavity wide and high.
K131 Skull: Nasal extension
(0) Nasal long and located anterior to the orbit, (1) Nasal short and posteriorly shifted .
K132 Skull: Nasal fossa
(0) Nasal fossa anterior, (1) Nasal fossa moderately retracted, (2) Nasal fossa strongly retracted.
K133 Skull: Premaxillary-frontal contact
(0) Premaxillary-frontal contact absent (separated by the nasal), (1) Premaxillary-frontal contact present.
K134 Skull: frontal and maxillary contact anterior to orbit
(0) absent or weak (seprated by lacrimal--nasal contact), (1) present, well developed (=lacrimal-nasal suture absent).
K135 Naso-frontals sutures
(0) Naso-frontals sutures convex posteriorly (nasals intrude in frontals), (1) Naso-frontals sutures straight transversely.
K136 Skull: Lacrimal
(0) Lacrimal present, (1) lacrimal absent.
K137 Skull:Lacrimal facial process
(0) Lacrimal facial process large, and in contact to nasal, (1) Lacrimal facial process large but with reduced or no contact to nasal, (2) Lacrimal facial process small, reduced along orbital rim, (3) Lacrimal facial process absent.
K138 Skull: Lacrimal tubercle
(0) Lacrimal tubercle absent, (1) lacrimal tubercle present.
K139 Skull: Lacrimal foramen
(0) anterior to orbit (facial), (1) in the orbit rim, (2) in the orbit lacrimal foramen, (3) absent.
K140 Skull: Orbit location

(0) Orbit very posterior, above M3/ or more posterior, (1) Orbit posterior, above M2/, (2) Orbit above M1/, (2) Orbit above P4/ or in between P/4 and M1/ level, (3) Orbit above P3/ or in between P4/ and P3/ level, 4 Orbit anterior to P3/ .

K141 Skull: Orbit lower rim construction

(0) Orbit bordered ventrally mostly or entirely by the jugal that joins the lacrimal (reduced zygomatic process of the maxillary), (1) orbit bordered ventrally partly by a short process of the maxillary extended between lacrimal and jugal, (2) maxillary forms the antorbital rim (jugal restricted distally to orbit).

K142 Skull: Infraorbital foramen size

(0) Infraorbital foramen small, (1) infraorbital foramen large.

K143 Skull: Infraorbital foramen position vs upper teeth

(0) Infraorbital foramen anterior to last premolar, (1) Infraorbital foramen above the last premolar, (1) Infraorbital foramen below M1/ or more posterior.

K144 Skull: infraorbital foramen position vs orbit

(0) Anterior to orbit (> one tooth length), long infraorbital canal, (1) Close to the orbit, short infraorbital canal, (2) Below to the orbit, short infraorbital canal.

K145 Skull: Submaxillary fossa

(0) Submaxillary fossa absent, (1) submaxillary fossa present.

K146 Skull: Frontal

(0) Frontal similar or a little shorter than parietal, (1) frontal very shortened, less than half length of parietal, (2) Frontal very elongated, longer than parietal.

K147 Skull: Tuber maxillae development

(0) Tuber maxillae absent/small, (1) tuber maxillae large (to inflated).

K148 Skull: Tuber maxillae location

(0) Tuber maxillae ventral or anterior to the orbit, (1) Tuber maxillae noticeably posterior to the orbit.

K149 Skull: Ascending process of the palatine extention in orbitotemporal fossa

(0) Ascending process of the palatine present in orbito-temporal fossa, (1) Ascending process of the palatine reduced in orbito-temporal fossa, (2) Ascending process of the palatine absent in the orbito-temporal fossa.

K150 Skull: Morphology of zygomatic arch at zygomatic process

(0) Zygoma narrow and slender without distinct ventral max. process, (1) Zygoma high dorso-ventrally + ventral max process (max. sut.).

K151 Skull: Distal extention of the jugal

(0) Jugal restricted distally to the anterior part of the glenoid fossa, 1 Jugal extended distally up to the distal part of the glenoid fossa.

K152 Skull: Postorbital constriction

(0) Postorbital constriction strong, (1) Postorbital constriction weak.

K153 Skull: Postorbital process of the frontal

(0) Postorbital process of the frontal developed and inflated, (1) postorbital process of the frontal reduced or absent.

K154 Skull: Postorbital process of the jugal

(0) Postorbital process of the jugal present but weak postorbital process of the jugal absent, (1) postorbital process of the jugal very developed.

K155 Skull: Zygomatic arch

(0) Zygomatic arch poorly divergent laterally, (1) zygomatic arch widely divergent laterally.

K156 Skull: Squamosal cerebral part development

(0) Cerebral part not extended dorsally, (1) Cerebral part extended and inflated (=scale).

K157 Skull: Squamosal zygomatic process development

(0) moderately developed laterally, (1) very well developed laterally and very robust.

K158 Skull: Parietal-alisphenoid contact

(0) Parietal-alisphenoid contact, (1) frontal-squamosal contact.

K159 Skull: Parietal

(0) Not extended more anteriorly than alisphenoid, (1) Extended more anteriorly than alisphenoid with an orbitosphenoid suture.

K160 Skull: Alisphenoid canal

(0) Absent, (1) Present.

K161 Skull: Foramen rotundum and sphenorbital fissure

(0) confluent, (1) separated.

K162 Skull: External auditory meatus (eam) elevation

(0) eam poorly elevated above the tooth row, (1) eam high above the tooth row.

K163 Skull: Post-tympanic process and external auditory meatus ventral rim

(0) Post-tympanic proc. reduced, eam opened ventrally, (1) Post-tympanic proc. developed, eam still opened ventrally, (2) Post-tympanic proc. joining postglenoid proc., eam closed ventrally.

K164 Skull: Sagittal crest

(0) Sagittal crest present all along top of skull (joining temporal crests), (1) Sagittal crest reduced to back of skull, (2) Sagittal crest weak or absent.

K165 Skull: Nuchal crests

(0) Nuchal crests present, (1) Nuchal crests reduced/absent.

K166 Skull: Periotic mastoidy/amastoidy

(0) Posterior mastoidy: large posterior (occipital) mastoid exposure, (1) Lateral mastoidy: narrow lateral mastoid exposure, (2) Amastoidy: no mastoid exposure.

K167 Skull: Periotic pars mastoidea relative development

(0) Pars mastoidea small, (1) Pars mastoidea larger than pars cochlearis.

K168 Skull: Periotic

(0) Fenestra vestibuli medium to large, (1) Fenestra vestibuli very small (<< f. cochleae).

K169 Skull: Periotic

(0) Fenestra vestibuli round with low stapedial ratio (< 1.8), (1) Fenestra vestibuli elliptical with high stapedial ratio (>1.8).

K170 Skull: Periotic

(0) Fenestra cochleae postero-medial to fenestra vestibuli, (1) Fenestra cochleae posterior to fenestra vestibuli.

K171 Skull: Periotic (pars cochlearis): Round window and cochlear canal

(0) Round window and cochlear canal present, (1) Perilymphatic for. present, no round window and cochlear canal.

K172 Skull: Periotic

(0) Subarcuata fossa present and deeply excavated, (1) Subarcuata fossa present but poorly excavated, (2) Subarcuata fossa absent.

K173 Preglenoid process

(0) absent, (1) present.

K174 Skull: Postglenoid foramen location

(0) Postglenoid foramen medial, (1) Postglenoid foramen distal, (2) Postglenoid foramen absent.

K175 Skull: Tegmen tympani

(0) small or absent inflated, (1) forming large and robust barrel-like bony structure.

K176 Skull: Jugular foramen

(0) Small (subequal to fenestra cochleae), (1) Large (>> fenestra cochleae).

K177 Skull: Midline keel at ventral surface of basioccipital

(0) Absent weak but present, (1) well developed.

K178 Skull: Hypoglossal foramen

(0) Present and isolated, (1) Coalescent with f. lacerum posterius.

K179 Skull: Ethmoidal foramen

(0) Ethmoidal f. far from optic f., (1) median in orbito-temporal fossa, (2) Ethmoidal f. posteriorly shifted.

K180 Skull: Inner ear Cochlea, Secondary bony spiral lamina

(0) Secondary bony spiral lamina present on the basal turn, (1) Secondary bony spiral lamina present on basal and higher turns, (2) Secondary bony spiral lamina absent.

K181 Skull: Inner ear Cochlea, number of turns of the cochlea

(0) 1.5 Number of turns of the cochlea between 1.5 and 2 turns, (1) Number of turns of the cochlea = 2 or more.

K182 Skull: : Inner ear, secondary crus commune

(0) present, (1) absent.

K183 Skull: Inner ear cochlea shape

(0) planispiral (cochlear ratio < 0.6), (1) conic (cochlear ratio > 0.6).

Part II

Character matrix for *Abdounodus hamdii*, taxa analyzed

Matrix
The matrix include 27 taxa and 184 characters. 16 characters are uninformative (2, 4, 8, 54, 55, 62, 66, 81, 86, 96, 133, 136, 159, 169, 171, 179). 44 characters (Table S12) are treated as additive, except when mentioned.

Eutheria
0000000[01]0000000000000000000000000000000000000000[01]10000[01]00[01]00000000000
000[01]00000[01]0?1[01]0000?000000000??00000000000?0000000000000[01]000000000
0010000000000000000000000000000[01]00000000000000
Abdounodus
????????1[12]????001000000010000010011[01][01]2121200[01]0[01]0100200001001 1?????????
???1100201[01]0200001[01]0111000011001110311111?01100000????1?????????????2??000?00??
????????????????????????????????
Anthracobunidae
0000000111000001 01[01]0011122011112011[01]000200212[01][12]2001??000[01]1001000000?01
000[01][01]1221211302100011 2111[23]0310??00110202111101311?0100?0??00?00???????[01]1?[
01]00?0??0?0000?0????0010???????1??????????
Arctocyonidae
0000000[01]1[01][01][01]0000[01]00000[01][01]10000011000000[12]0100122[01]0000020000[12][
01]0[01]00000000101000110010 10?210001001[01]020[12][01]0??00110102000?011[01][01]00000
000[01]000000100111[01]0100010000000[01]10000[01]10000[01]001000[01]100000??0?
Cambaytherium
0000000[12]1[01]00000010?0100111011112011[01]00222021010 2001??00012?0??0000010100000
1220211202100[01]11111120310??00110[12]021110013[01]??01?0?02?????0?11?1??20???0?1??0
0?01??0??1??0?????????0??????????
Desmostylia
110000001[01][12]1000010?00011220111000111001200[01]1[12][12]02001?[01]100011[01]200100
00010?00012[12]1[12]110?21001112111[23]0?10??1?120??21111[01]1311?0100?0021?10[01]0100
[23]123011[12]0211000000001??101[12]00200??010???200????
Embrithopoda
??0?0?031011000101100110 3?111111112100021 00[01]2[01][01]202??20000201210 1???01101000
12?010120200[01]11??0001000210031024211?1?013111[01]10000101111011021?3[12]11100011
10000101001011?02000?1[12]02011112210
Eritherium
11010?1410212100101010102201 11[01]101110122222[01][01][01]01010020????11[12]?????0110
100012[01]0101[01]?20001111111200011101120421111?[01]11111010??00???1000?00??2311000
?01?1?00?1????????0??????????????????
Hyopsodus
000?010[23]10211000000000110100 01[01][01][02]0001000[01]100[01]2[12]00000[01]20000110100
110001101000110010 1[01]?210001001102021 0??0011020[02]000001100000000200000?000100102
0010001000[01]010001100100010001000011 01200??01
Hyracoidea
?10?0?0310[01]10001001000102201110201110111 20[01]1[12]10200100000020 1[12]1012?001[01]
0001212[12]010120200001[12]2001000011[01]0[12]1[01]0421121?0112110100000101000 0100202
[12][01]0[01]000[01][01]00100[12]0000[01]110100200100102012000110
Macroscelidea
010[012]0?031[01]1[01]000[01]01100110101000010000101 1[01]00020[01]1010[01]2?00[01]2[01][0

1][01]0012000[01]00000011110113[01]20[01]0011111120310??00121[12]00021001210011000002[0
1]110000000[12]1220[01]1000000010101000[01]00?000??0?00011?[12]001201
Minchenella
????????1???00011110011121011102011100021021002200????1???????0??????????????????????
????11200120310??0000020102[01]001311001???
????????????????
Numidotherium
11020?25?33?00001[01]10111032011110112000122000022[12]20213110102011201010211?01112
1010110200111311103000??1?120421131?113211011201?11111001?0112421011001211001111
10111200211110100002100100
Ocepeia
0000001[12]1[12]3?3?0010101000100[01]00120110111010002[01]10021230000200110 1?111011?
1??12002010?2000010000100011100200021000 0?01300000203011001000000 0?2010000100?1?0
1000?010?0000211100000112 0?1210
Orycteropus
??1?1?25?[12][23]121101??1??????2?????????????????021012000?0002210103????22?1?[01]2?2??
???3?????0?1?????????????????????????11???310111041010001001100002000000001000100[01]
0000200000002020100 0[012][01]00
Paschatherium
???????3100000001000000000000111000000[01]20000121010?0030???????0??????????????11001
1[12]0211000000012021 0??001[12]0100000?00200000???????????????????????????????????????
???????????????????????
Perissodactyla
000000011[01]2[01]000101000111210[01]11020111101200012[12]020010[12]0000210[12]00000[0
2]00100000122[01]01131210001221[01]120310??00110[12]0[02]1211011110000100[02]1[02]1000
0010002[012]00100011000000000001100[01]1100100?010100002??
Phenacodontidae
0000000[01]1121000[01]0000011[01]10000002000[01][01]10[01]10012[12]01000020000210100000
0[02]0010000011201113121000 1[01]0101203110[01]01110102[01]1100111[01]0000020220100000
00[01][01]200[01][012]001100000000000010010000000 0?11012000???
Phenacolophus
00000003100100000000????310100021110000210012 2[01]200???100?????0??????????020?0000?
????????1111[01]12031110020102020110003[12]1?01???????????????????0?????????????????????
??????????????????????
Phosphatherium
11020?25?021[12]100101010103 2[01]111101120[01]122211022[12]2020[01][23]000010112?????0
1101000121010110200111311103000111011204211[23]1?113[12]112102202101100 0100[23]1241
1001001?1?000101101?110021001000000 1000???
Potamogale
100?0103103?000010[01]0001010000000000000000?0001000020[01]30000100001200010 1?00011
001010?100000?00011000[01]00?00010?1000?01100000001023110000101313[12]210201000??11
??1?001000010??100001011100 2?0
Protungulatum
0000000[01]101100000000001010010001000 10010[01]0012020000[01][12]000020020??????010?0
??11000010?1100010000120110??00000102000?01100000??????????????????????????00????????
??????????001000??0????????
Ptolemaia
???????0103?000000100010102000000000000200000110120 01[12]00000100000100001 1?00?12???
011010000100110300 00??0011000[01]000?0220?02000?0??1?000???0[23]?1001000000???00????
????0000??????????????????
Radinskya
???0000012??01110210

0012211120310??00110[12]02121101111?0000?02?20000011011?10000001[01]00000?000??1000
01100?00?01??0000???
Sirenia
11000103[01]0[12]1[12]11???110?1022011100011100120?202122021?[12]10002012?01200011010
20[02]0?0?00??????111120[23]000???0?12042?111?113111010000?1[01]111211?0212501021001
0100001010010112020[01]00[12]02[01]10002200
Teilhardimys
????????101?000010101000121011100001101100012[12]010000300????0?0??????????0121210[1
2]112[01]2100011111121310??00111[12]0[02][01]21001210011???????????????????????????????
???????????????????????????????
Todralestes
???????1111100000010001000000000000000010000011000?023000000000??????????00?0???0010
?1000000000000110??00000100000?00000000???????????????????????0??????????????????????
????????????????

Taxa analysed

The primary aim of our analysis was a comparison *Abdounodus hamdii* with lophodont and archaic ungulate-like mammals in order to assess its ordinal relationships. The basal out-group taxon (Eutheria) corresponds to the generalized eutherian morphotype.

27 terminal taxa

0 Eutheria
1 Abdounodus
2 Anthracobunidae
3 Arctocyonidae
4 Cambaytherium
5 Desmostylia
6 Embrithopoda
7 Eritherium
8 Hyopsodus
9 Hyracoidea
10 Macroscelidea
11 Minchenella
12 Numidotherium
13 Ocepeia
14 Orycteropus
15 Paschatherium
16 Perissodactyla
17 Phenacodontidae
18 Phenacolophus
19 Phosphatherium
20 Potamogale
21 Protungulatum
22 Ptolemaia
23 Radinskya
24 Sirenia
25 Teilhardimys
26 Todralestes

The taxonomic sampling of our analysis includes a selection of condylarth-grade taxa, basal lophodont ungulate-like taxa, and the main lineages of afrotherians. Most taxa included in the analysis are genera, but we also compared some suprageneric taxa to *Abdounodus.* Use of suprageneric taxa instead genera was made for lineages with incomplete fossil record in order to increase their character completness in the matrix. The character coding of these suprageneric taxa is based on their ancestral morphotype that is reconstructed based on their most basal known genera:

- Anthracobunidae: *Anthracobune, Indobune.*
- Arctocyonids: *Loxolophus*, *Tricentes, Lambertocyon*, *Arctocyon.*
- Desmostylians: *Behemotops* and others.
- Embrithopods : *Namatherium*, Palaeoamasiidae incl. *Crivadiatherium*, *Arsinoitherium*
- Eutheria: *Acristatherium*, leptictids (*Leptacodon*, *Leptictis*), cimolestids (*Cimolestes, Maelestes*).
- Hyracoids: *Seggeurius*, *Microhyrax, Helioseus,* Pliohyracidae.
- Macroscelids: *Chambius*, *Herodotius, ?Metoldobotes* and extant genera (*Petrodromus*, *Rhynchocyon*).
- Perissodactyles: *Hyracotherium*, *Cymbalophus*, *Pachynolophus.*
- Phenacodontids : *Ectocion, Phenacodus.*

- Sirenians: *Prorastomus*, *Pezosiren*, *Protosiren*, *Eosiren*, *Eotheroides*, *Prototherium.*

Part III

TNT analysis, method, cladograms, and characters at nodes

The parsimony analysis was developed by means of the TNT program. We used the “traditional search” command for all analyses. Bremer indices were calculated for 10000 trees with additional 10 steps longer than in the shortest obtained tree. The 16 uninformative characters were made inactive before the analysis.

The interface WINCLADA associated with the heuristic algorithm NONA was used in complement of our study, especially for the revision of the matrix, for the preliminary explorative analysis of tree topology and for examination of character distribution in trees.

1. Analysis 1 *:* 44 characters additive and unweighted analysis

1.1 Cladogram 1

Traditional search, 1 tree found.

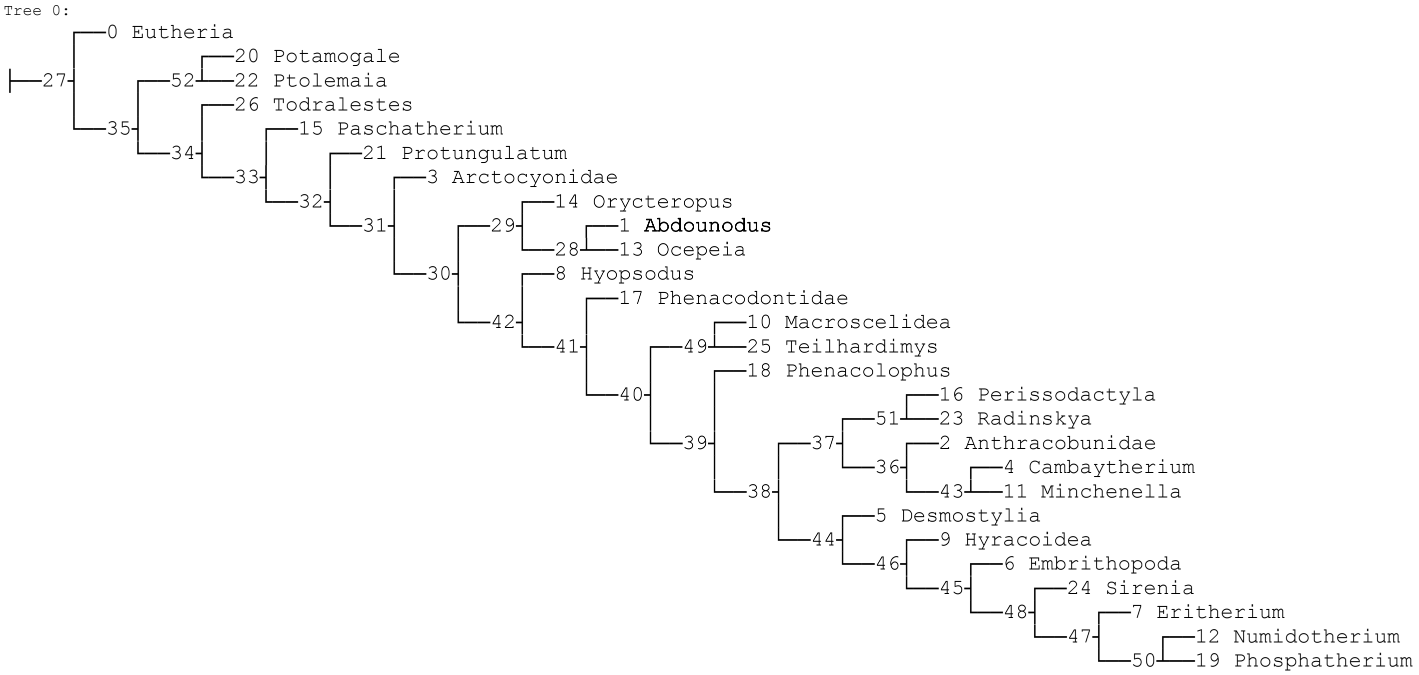

Tree length : 736. Retention index: 53.0. Consistency Index: 37.0.

Cladogram 1, Homoplasy

	+0	+1	+2	+3	+4	+5	+6	+7	+8	+9
0	1	1	--	0	--	2	2	13	--	3
10	9	2	4	2	1	3	6	3	5	1
20	3	5	4	1	4	2	2	1	3	2
30	7	7	2	1	2	5	2	4	10	2
40	7	2	6	3	5	6	4	4	2	6
50	3	1	5	4	--	--	1	5	4	0
60	2	1	--	3	7	1	--	3	3	2
70	2	2	--	4	3	1	6	3	2	5
80	2	--	6	1	1	3	--	0	1	1
90	0	2	5	6	5	4	--	5	2	4
100	1	0	1	3	5	7	1	2	1	7
110	2	4	1	1	0	2	7	1	0	0
120	1	2	1	1	3	1	3	1	4	3
130	2	2	1	--	4	1	--	4	3	0
140	4	5	3	3	3	1	3	3	0	1
150	1	1	3	1	3	1	4	2	0	--
160	1	3	1	1	4	1	1	1	2	4
170	0	--	2	2	3	1	2	5	2	--
180	4	3	2	1						

1.2 Bremer supports (from 10000 trees, cut 2)

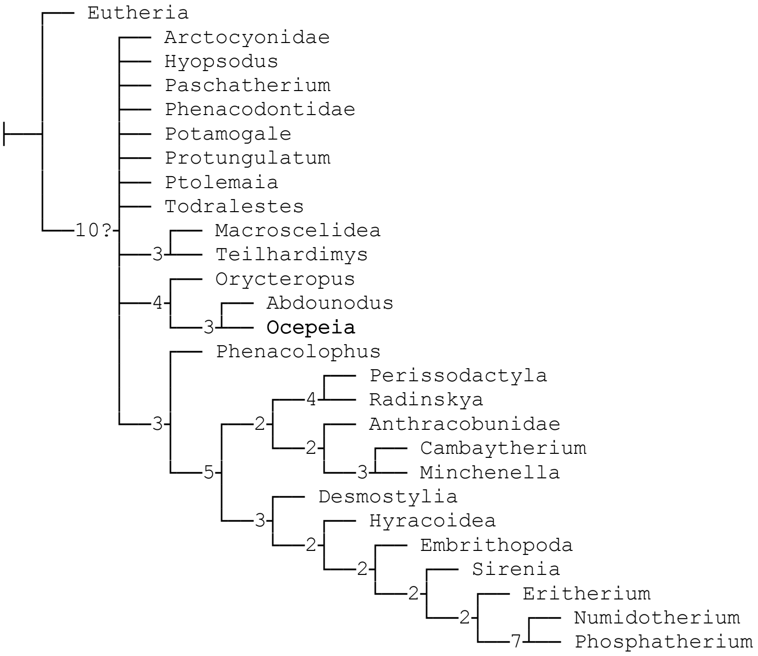

1.2. Synapomorphies for cladogram 1

Eutheria :
No autapomorphies
Abdounodus :
Char. 31: 2 --> 0
Char. 38: 1 --> 2
Char. 40: 1 --> 2
Char. 41: 0 --> 1
Char. 42: 0 --> 2
Char. 44: 2 --> 0
Char. 57: 2 --> 1
Char. 98: 1 --> 0
Char. 107: 2 --> 3
Char. 110: 0 --> 1
Char. 111: 0 --> 1
Char. 112: 0 --> 1
Char. 140: 0 --> 2
Anthracobunidae :
Char. 25: 1 --> 2
Char. 57: 2 --> 1
Char. 78: 0 --> 1
Char. 141: 0 --> 1
Char. 173: 0 --> 1
Arctocyonidae :
Char. 45: 1 --> 2
Char. 94: 1 --> 0
Char. 155: 0 --> 1
Char. 161: 0 --> 1
Cambaytherium :
Char. 15: 1 --> 0
Char. 17: 1 --> 0
Char. 20: 0 --> 1
Char. 21: 1 --> 0
Char. 22: 1 --> 0
Char. 24: 2 --> 1
Char. 38: 0 --> 2
Char. 91: 2 --> 1
Desmostylia :
Char. 7: 123 --> 0
Char. 16: 0 --> 1
Char. 53: 0 --> 1
Char. 57: 2 --> 1
Char. 78: 0 --> 1
Char. 102: 0 --> 1
Char. 105: 1 --> 2
Char. 127: 1 --> 2
Char. 128: 0 --> 1
Char. 144: 0 --> 12
Char. 146: 0 --> 2
Embrithopoda :
Char. 17: 0 --> 1
Char. 21: 0 --> 1
Char. 24: 2 --> 3
Char. 26: 0 --> 1
Char. 32: 0 --> 1
Char. 34: 1 --> 2
Char. 38: 1 --> 0
Char. 40: 02 --> 1
Char. 95: 2 --> 1
Char. 99: 1 --> 2
Char. 103: 1 --> 3
Char. 105: 1 --> 0
Char. 106: 0 --> 2
Char. 135: 0 --> 1
Char. 144: 0 --> 1
Char. 178: 0 --> 1
Eritherium :
Char. 12: 1 --> 2
Char. 41: 01 --> 2
Char. 44: 2 --> 01
Char. 47: 2 --> 1
Char. 49: 2 --> 1
Char. 94: 0 --> 1
Char. 116: 3 --> 1
Hyopsodus :
Char. 5: 0 --> 1
Char. 28: 0 --> 1
Char. 57: 2 --> 1
Char. 64: 0 --> 1
Char. 68: 0 --> 1
Char. 94: 1 --> 0
Char. 127: 1 --> 0
Char. 138: 1 --> 0
Char. 152: 0 --> 1
Char. 156: 0 --> 1
Char. 157: 0 --> 1
Char. 164: 0 --> 1
Char. 168: 0 --> 1
Hyracoidea :
Char. 37: 0 --> 1
Char. 39: 2 --> 1
Char. 73: 0 --> 1
Char. 74: 0 --> 2
Char. 95: 2 --> 0
Char. 111: 1 --> 2
Char. 116: 3 --> 1
Char. 117: 1 --> 2
Char. 138: 1 --> 0
Char. 142: 1 --> 0
Char. 151: 0 --> 1
Char. 154: 0 --> 12
Char. 161: 0 --> 1
Char. 181: 2 --> 1
Macroscelidea :
Char. 17: 0 --> 1
Char. 43: 1 --> 0
Char. 45: 1 --> 0
Char. 49: 0 --> 1
Char. 78: 0 --> 1
Char. 85: 1 --> 0
Char. 105: 1 --> 2
Minchenella :
Char. 45: 1 --> 0
Char. 53: 0 --> 1
Char. 92: 1 --> 0
Char. 93: 1 --> 0
Char. 104: 1 --> 0
Char. 105: 1 --> 0
Char. 109: 2 --> 1
Char. 110: 1 --> 0
Char. 111: 1 --> 2
Numidotherium :
Char. 9: 0 --> 3
Char. 10: 2 --> 3
Char. 12: 1 --> 0
Char. 13: 1 --> 0
Char. 21: 0 --> 1
Char. 42: 1 --> 0
Char. 51: 0 --> 3
Char. 52: 2 --> 1
Char. 53: 0 --> 1
Char. 67: 0 --> 2
Char. 70: 0 --> 1
Char. 73: 0 --> 1
Char. 74: 0 --> 1
Char. 99: 1 --> 0
Char. 102: 0 --> 1
Char. 122: 0 --> 1
Char. 125: 0 --> 1
Char. 128: 0 --> 1
Char. 137: 2 --> 1
Char. 141: 1 --> 2
Char. 144: 0 --> 1
Char. 154: 0 --> 1
Char. 156: 0 --> 1
Char. 163: 1 --> 2
Char. 168: 0 --> 1
Char. 178: 0 --> 1
Ocepeia :
Char. 18: 0 --> 1
Char. 20: 0 --> 1
Char. 46: 0 --> 1
Char. 49: 1 --> 2
Char. 50: 0 --> 1
Char. 51: 0 --> 2
Char. 52: 2 --> 3
Char. 76: 1 --> 2
Char. 92: 1 --> 0
Char. 104: 1 --> 0
Char. 105: 1 --> 0
Char. 109: 1 --> 0
Char. 116: 1 --> 3
Orycteropus :
Char. 6: 1 --> 2
Char. 7: 2 --> 5
Char. 14: 0 --> 1
Char. 19: 0 --> 1
Char. 26: 0 --> 2
Char. 47: 0 --> 1
Char. 48: 0 --> 2
Char. 56: 0 --> 2
Char. 67: 1 --> 2
Char. 68: 0 --> 2
Char. 75: 1 --> 2
Char. 120: 0 --> 3
Char. 121: 0 --> 1
Char. 123: 0 --> 1
Char. 125: 0 --> 1
Char. 127: 1 --> 4
Char. 128: 0 --> 1
Char. 139: 2 --> 0
Char. 143: 0 --> 2
Char. 146: 1 --> 0
Char. 152: 0 --> 1
Char. 156: 0 --> 1
Char. 164: 0 --> 2
Char. 172: 0 --> 2
Char. 181: 2 --> 01
Paschatherium :
Char. 7: 1 --> 3

Char. 10: 1 --> 0
Char. 16: 0 --> 1
Char. 22: 1 --> 0
Char. 28: 0 --> 1
Char. 29: 0 --> 1
Char. 38: 1 --> 2
Char. 47: 0 --> 1
Char. 77: 0 --> 1
Char. 80: 0 --> 1
Char. 82: 0 --> 12
Char. 97: 1 --> 2
Perissodactyla :
Char. 83: 0 --> 1
Char. 123: 0 --> 1
Char. 134: 1 --> 0
Char. 137: 1 --> 0
Char. 138: 1 --> 0
Char. 161: 0 --> 1
Phenacodontidae :
Char. 7: 2 --> 01
Char. 9: 0 --> 1
Char. 37: 0 --> 1
Char. 63: 1 --> 0
Char. 83: 0 --> 1
Char. 99: 0 --> 1
Char. 103: 0 --> 1
Char. 107: 2 --> 1
Char. 127: 1 --> 2
Phenacolophus :
Char. 10: 1 --> 0
Char. 32: 0 --> 1
Char. 45: 1 --> 2
Char. 53: 0 --> 1
Char. 73: 0 --> 2
Char. 76: 1 --> 0
Char. 99: 0 --> 1
Char. 103: 0 --> 2
Char. 104: 1 --> 0
Char. 115: 1 --> 0
Phosphatherium :
Char. 57: 2 --> 1
Char. 120: 0 --> 2
Char. 124: 0 --> 2
Char. 172: 1 --> 0
Potamogale :
Char. 7: 1 --> 3
Char. 16: 0 --> 1
Char. 49: 0 --> 2
Char. 63: 0 --> 1
Char. 64: 1 --> 2
Char. 68: 0 --> 1
Char. 94: 0 --> 1
Char. 106: 0 --> 1
Char. 139: 1 --> 3
Char. 140: 0 --> 12
Char. 141: 0 --> 2
Char. 144: 0 --> 2
Char. 152: 0 --> 1
Char. 153: 0 --> 1
Char. 165: 0 --> 1
Protungulatum :
Char. 27: 0 --> 1
Char. 45: 1 --> 0
Char. 46: 0 --> 2
Ptolemaia :
Char. 7: 1 --> 0
Char. 26: 0 --> 2
Char. 38: 0 --> 2
Char. 47: 0 --> 1
Char. 48: 0 --> 2
Char. 76: 1 --> 2
Char. 82: 0 --> 1
Char. 89: 0 --> 1
Char. 92: 0 --> 1
Char. 93: 0 --> 1
Char. 104: 0 --> 1
Char. 105: 0 --> 1
Char. 120: 0 --> 2
Radinskya :
Char. 82: 3 --> 1
Char. 143: 1 --> 0
Sirenia :
Char. 14: 0 --> 1
Char. 19: 0 --> 1
Char. 53: 0 --> 1
Char. 73: 0 --> 2
Char. 75: 1 --> 02
Char. 76: 2 --> 0
Char. 132: 01 --> 2
Char. 140: 3 --> 5
Char. 144: 0 --> 2
Char. 164: 0 --> 2
Char. 177: 12 --> 0
Teilhardimys :
Char. 16: 0 --> 1
Char. 20: 0 --> 1
Char. 21: 1 --> 0
Char. 22: 1 --> 0
Char. 25: 0 --> 2
Char. 28: 0 --> 1
Char. 29: 0 --> 1
Char. 30: 0 --> 1
Char. 52: 2 --> 3
Char. 73: 0 --> 1
Char. 74: 0 --> 2
Char. 76: 1 --> 2
Char. 82: 3 --> 2
Todralestes :
Char. 9: 0 --> 1
Char. 75: 1 --> 0
Node 28 :
Char. 49: 0 --> 1
Char. 61: 0 --> 1
Char. 126: 0 --> 1
Node 29 :
Char. 6: 0 --> 1
Char. 9: 0 --> 12
Char. 12: 01 --> 2
Char. 16: 0 --> 1
Char. 43: 1 --> 0
Char. 67: 0 --> 1
Char. 70: 0 --> 1
Char. 130: 0 --> 1
Char. 142: 1 --> 0
Node 30 :
Char. 7: 1 --> 2
Char. 10: 1 --> 2
Char. 31: 1 --> 2
Char. 63: 0 --> 1
Char. 107: 1 --> 2
Char. 139: 1 --> 2
Node 31 :
Char. 79: 0 --> 1
Char. 92: 0 --> 1
Node 32 :
Char. 24: 0 --> 1
Char. 31: 0 --> 1
Char. 52: 3 --> 2
Char. 89: 0 --> 1
Char. 109: 0 --> 2
Char. 115: 0 --> 1
Node 33 :
Char. 43: 0 --> 1
Char. 44: 1 --> 2
Char. 85: 0 --> 1
Char. 94: 0 --> 1
Char. 95: 0 --> 2
Node 34 :
Char. 38: 0 --> 1
Char. 97: 0 --> 1
Char. 98: 0 --> 1
Char. 107: 0 --> 1
Node 35 :
No synapomorphies
Node 36 :
Char. 10: 1 --> 0
Char. 42: 0 --> 2
Node 37 :
Char. 17: 0 --> 1
Char. 63: 1 --> 0
Char. 135: 0 --> 1
Node 38 :
Char. 28: 0 --> 1
Char. 29: 0 --> 1
Char. 76: 1 --> 2
Char. 91: 1 --> 2
Char. 110: 0 --> 1
Char. 113: 0 --> 1
Node 39 :
Char. 25: 0 --> 1
Char. 27: 0 --> 1
Char. 33: 0 --> 1
Char. 34: 0 --> 1
Char. 39: 1 --> 2
Char. 47: 1 --> 2
Char. 118: 0 --> 1
Node 40 :
Char. 10: 2 --> 1
Char. 91: 0 --> 1
Char. 121: 0 --> 1
Char. 146: 1 --> 0
Node 41 :
Char. 47: 0 --> 1
Char. 80: 0 --> 1
Char. 82: 0 --> 3
Char. 97: 2 --> 3
Char. 111: 0 --> 1
Char. 112: 0 --> 1
Char. 117: 0 --> 1
Char. 126: 0 --> 2
Char. 129: 0 --> 1
Node 42 :
Char. 21: 0 --> 1
Char. 97: 1 --> 2
Node 43 :
Char. 16: 0 --> 1
Char. 44: 2 --> 0
Char. 113: 1 --> 0
Node 44 :
Char. 0: 0 --> 1

Char. 1: 0 --> 1
Char. 21: 1 --> 0
Char. 25: 1 --> 2
Char. 60: 1 --> 2
Char. 126: 2 --> 0
Char. 137: 1 --> 2

Node 45 :
Char. 49: 0 --> 2
Char. 71: 0 --> 1
Char. 94: 1 --> 0
Char. 150: 0 --> 1
Char. 155: 0 --> 1

Node 46 :
Char. 58: 1 --> 0
Char. 61: 0 --> 1
Char. 68: 0 --> 1
Char. 80: 1 --> 0
Char. 85: 1 --> 0
Char. 98: 1 --> 0
Char. 99: 0 --> 1
Char. 109: 2 --> 1

Node 47 :
Char. 3: 0 --> 1
Char. 6: 0 --> 1
Char. 7: 3 --> 4
Char. 20: 0 --> 1
Char. 60: 2 --> 1

Node 48 :
Char. 12: 0 --> 1
Char. 13: 0 --> 1
Char. 42: 0 --> 12
Char. 105: 1 --> 2
Char. 114: 0 --> 1
Char. 143: 1 --> 0

Node 49 :
Char. 26: 0 --> 1
Char. 36: 0 --> 1
Char. 106: 0 --> 1
Char. 111: 1 --> 2
Char. 120: 0 --> 1

Node 50 :
Char. 3: 1 --> 2
Char. 6: 1 --> 2
Char. 7: 4 --> 5
Char. 24: 2 --> 3
Char. 32: 0 --> 1
Char. 34: 1 --> 2
Char. 35: 1 --> 0
Char. 45: 1 --> 2
Char. 87: 0 --> 1
Char. 90: 1 --> 3
Char. 95: 2 --> 3
Char. 111: 1 --> 3
Char. 140: 3 --> 4

Node 51 :
Char. 90: 1 --> 2
Char. 111: 1 --> 2
Char. 116: 3 --> 1
Char. 121: 1 --> 0
Char. 165: 0 --> 1

Node 52 :
Char. 10: 1 --> 3
Char. 24: 0 --> 1
Char. 58: 0 --> 1
Char. 64: 0 --> 1
Char. 70: 0 --> 1
Char. 128: 0 --> 1
Char. 137: 1 --> 23

2. Analysis 2 *:* 44 characters additive and analysis with implied weigthing

2.1 Cladogram 2

Standard weighting strength is 3.00000.

Traditional search, 1 tree found.

Best score (TBR): 75.83690.

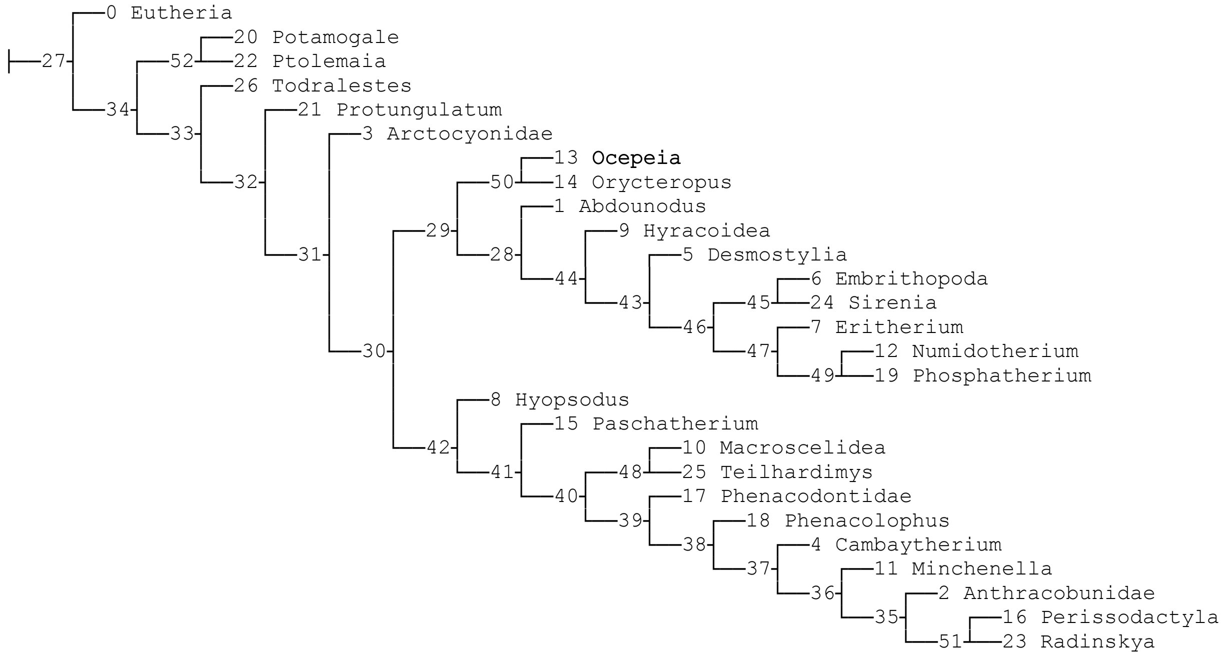

Node 29 = **Paenungulatomorpha**

Tree length : 746. Retention index: 52.0. Consistency Index: 36.5.

Cladogram 2, homoplasy

	+0	+1	+2	+3	+4	+5	+6	+7	+8	+9
0	1	1	--	0	--	2	2	12	--	4
10	10	2	5	3	1	2	7	2	5	1
20	3	5	5	1	5	2	2	2	4	3
30	7	6	2	1	2	5	2	4	9	2
40	7	2	8	4	7	6	4	4	2	7
50	4	1	6	4	--	--	1	5	4	0
60	2	2	--	2	7	1	--	3	4	2
70	2	2	--	4	3	1	7	2	2	7
80	1	--	6	1	0	3	--	0	0	2
90	1	3	6	7	5	4	--	1	2	4
100	1	0	1	2	4	6	1	2	0	8
110	2	4	1	1	1	3	7	2	1	0
120	1	4	1	1	3	1	3	1	4	4
130	1	1	0	--	4	1	--	4	3	0
140	3	5	3	4	3	1	4	2	1	1
150	1	1	3	1	3	1	4	1	0	--
160	1	3	0	1	4	1	1	1	2	4
170	0	--	2	2	2	1	2	5	2	--
180	2	3	2	0						

2.2. Synapomorphies for cladogram 2

Eutheria :
No autapomorphies
Abdounodus :
Char. 38: 1 --> 2
Char. 41: 0 --> 1
Char. 42: 1 --> 2
Char. 44: 2 --> 0
Char. 49: 0 --> 1
Char. 57: 2 --> 1
Char. 126: 0 --> 1
Anthracobunidae :
Char. 25: 1 --> 2
Char. 30: 0 --> 1
Char. 57: 2 --> 1
Char. 58: 1 --> 0
Char. 141: 0 --> 1
Char. 147: 1 --> 0
Char. 164: 0 --> 1
Char. 173: 0 --> 1
Arctocyonidae :
Char. 146: 0 --> 1
Char. 155: 0 --> 1
Char. 161: 0 --> 1
Cambaytherium :
Char. 20: 0 --> 1
Char. 22: 1 --> 0
Char. 30: 0 --> 1
Char. 38: 0 --> 2
Char. 40: 1 --> 2
Char. 56: 0 --> 1
Char. 67: 0 --> 1
Char. 154: 0 --> 1
Desmostylia :
Char. 7: 3 --> 0
Char. 23: 0 --> 1
Char. 53: 0 --> 1
Char. 57: 2 --> 1
Char. 58: 0 --> 1
Char. 61: 1 --> 0
Char. 78: 0 --> 1
Char. 80: 0 --> 1
Char. 85: 0 --> 1
Char. 98: 0 --> 1
Char. 99: 1 --> 0
Char. 102: 0 --> 1
Char. 109: 1 --> 2
Char. 127: 1 --> 2
Char. 128: 0 --> 1
Char. 143: 0 --> 1
Char. 146: 0 --> 2
Char. 147: 0 --> 1
Embrithopoda :
Char. 24: 2 --> 3
Char. 26: 0 --> 1
Char. 31: 0 --> 1
Char. 32: 0 --> 1
Char. 34: 1 --> 2
Char. 38: 1 --> 0
Char. 42: 1 --> 0
Char. 92: 1 --> 0
Char. 95: 23 --> 1
Char. 105: 2 --> 0
Char. 106: 0 --> 2
Char. 143: 0 --> 1
Char. 178: 0 --> 1
Char. 182: 0 --> 1
Eritherium :
Char. 12: 01 --> 2
Char. 31: 0 --> 1
Char. 41: 01 --> 2
Char. 42: 1 --> 2
Char. 44: 2 --> 01
Char. 47: 2 --> 1
Char. 116: 3 --> 1
Hyopsodus :
Char. 5: 0 --> 1
Char. 10: 1 --> 2
Char. 12: 0 --> 1
Char. 35: 0 --> 1
Char. 57: 2 --> 1
Char. 124: 0 --> 2
Char. 127: 1 --> 0
Char. 138: 1 --> 0
Char. 146: 0 --> 1
Char. 157: 0 --> 1
Char. 164: 0 --> 1
Char. 168: 0 --> 1
Char. 173: 0 --> 1
Hyracoidea :
Char. 15: 0 --> 1
Char. 16: 1 --> 0
Char. 31: 0 --> 2
Char. 64: 01 --> 2
Char. 73: 0 --> 1
Char. 74: 0 --> 2
Char. 92: 1 --> 0
Char. 111: 1 --> 2
Char. 138: 1 --> 0
Char. 151: 0 --> 1
Char. 154: 0 --> 12
Char. 161: 0 --> 1
Char. 182: 0 --> 1
Macroscelidea :
Char. 17: 0 --> 1
Char. 31: 0 --> 1
Char. 43: 1 --> 0
Char. 45: 1 --> 0
Char. 49: 0 --> 1
Char. 78: 0 --> 1
Char. 85: 1 --> 0
Char. 105: 1 --> 2
Minchenella :
Char. 18: 0 --> 1
Char. 45: 1 --> 0
Char. 53: 0 --> 1
Char. 92: 1 --> 0
Char. 104: 1 --> 0
Char. 105: 1 --> 0
Char. 109: 2 --> 1
Numidotherium :
Char. 9: 0 --> 3
Char. 10: 2 --> 3
Char. 21: 0 --> 1
Char. 42: 1 --> 0
Char. 51: 0 --> 3
Char. 52: 2 --> 1
Char. 53: 0 --> 1
Char. 67: 0 --> 2
Char. 70: 0 --> 1
Char. 73: 0 --> 1
Char. 74: 0 --> 1
Char. 99: 1 --> 0
Char. 102: 0 --> 1
Char. 122: 0 --> 1
Char. 125: 0 --> 1
Char. 128: 0 --> 1
Char. 131: 0 --> 1
Char. 137: 2 --> 1
Char. 141: 1 --> 2
Char. 154: 0 --> 1
Char. 156: 0 --> 1
Char. 163: 1 --> 2
Char. 168: 0 --> 1
Char. 178: 0 --> 1
Ocepeia :
Char. 7: 3 --> 12
Char. 12: 2 --> 3
Char. 46: 0 --> 1
Char. 49: 0 --> 2
Char. 50: 0 --> 1
Char. 51: 0 --> 2
Char. 116: 1 --> 3
Char. 122: 0 --> 2
Char. 126: 0 --> 1
Char. 134: 1 --> 0
Char. 137: 1 --> 0
Char. 141: 0 --> 1
Char. 146: 0 --> 1
Char. 150: 0 --> 1
Char. 153: 0 --> 1
Char. 167: 0 --> 1
Char. 168: 0 --> 1
Char. 174: 2 --> 0
Char. 175: 0 --> 1
Char. 182: 0 --> 1
Orycteropus :
Char. 6: 1 --> 2
Char. 7: 3 --> 5
Char. 14: 0 --> 1
Char. 19: 0 --> 1
Char. 26: 0 --> 2
Char. 47: 0 --> 1
Char. 48: 0 --> 2
Char. 56: 0 --> 2
Char. 58: 0 --> 1
Char. 67: 1 --> 2
Char. 68: 01 --> 2
Char. 75: 1 --> 2
Char. 120: 0 --> 3
Char. 121: 0 --> 1
Char. 123: 0 --> 1
Char. 125: 0 --> 1
Char. 127: 1 --> 4
Char. 128: 0 --> 1
Char. 139: 2 --> 0
Char. 143: 0 --> 2
Char. 152: 0 --> 1
Char. 156: 0 --> 1
Char. 164: 0 --> 2
Char. 172: 01 --> 2
Char. 177: 2 --> 0
Paschatherium :

Char. 10: 1 --> 0
Char. 11: 1 --> 0
Char. 16: 0 --> 1
Char. 22: 1 --> 0
Char. 24: 1 --> 0
Char. 29: 0 --> 1
Char. 38: 01 --> 2
Char. 52: 2 --> 3
Char. 89: 1 --> 0
Char. 92: 1 --> 0
Char. 115: 1 --> 0
Perissodactyla :
Char. 83: 0 --> 1
Char. 123: 0 --> 1
Char. 137: 1 --> 0
Char. 138: 1 --> 0
Char. 161: 0 --> 1
Phenacodontidae :
Char. 10: 1 --> 2
Char. 37: 0 --> 1
Char. 83: 0 --> 1
Char. 103: 0 --> 1
Char. 124: 0 --> 2
Char. 127: 1 --> 2
Char. 146: 0 --> 1
Char. 163: 0 --> 1
Char. 173: 0 --> 1
Phenacolophus :
Char. 24: 1 --> 3
Char. 32: 0 --> 1
Char. 45: 1 --> 2
Char. 53: 0 --> 1
Char. 73: 0 --> 2
Char. 76: 1 --> 0
Char. 77: 2 --> 0
Char. 103: 0 --> 2
Char. 104: 1 --> 0
Char. 115: 1 --> 0
Phosphatherium :
Char. 57: 2 --> 1
Char. 120: 0 --> 2
Char. 124: 0 --> 2
Char. 172: 1 --> 0
Potamogale :
Char. 7: 1 --> 3
Char. 16: 0 --> 1
Char. 49: 0 --> 2
Char. 63: 0 --> 1
Char. 64: 1 --> 2
Char. 68: 0 --> 1

Char. 94: 0 --> 1
Char. 106: 0 --> 1
Char. 139: 1 --> 3
Char. 140: 0 --> 12
Char. 141: 0 --> 2
Char. 144: 0 --> 2
Char. 146: 0 --> 1
Char. 152: 0 --> 1
Char. 153: 0 --> 1
Char. 165: 0 --> 1
Protungulatum :
Char. 27: 0 --> 1
Char. 35: 0 --> 1
Char. 46: 0 --> 2
Ptolemaia :
Char. 7: 1 --> 0
Char. 26: 0 --> 2
Char. 38: 0 --> 2
Char. 47: 0 --> 1
Char. 48: 0 --> 2
Char. 76: 1 --> 2
Char. 82: 0 --> 1
Char. 89: 0 --> 1
Char. 92: 0 --> 1
Char. 93: 0 --> 1
Char. 104: 0 --> 1
Char. 105: 0 --> 1
Char. 120: 0 --> 2
Radinskya :
Char. 82: 3 --> 1
Sirenia :
Char. 14: 0 --> 1
Char. 19: 0 --> 1
Char. 42: 1 --> 2
Char. 46: 0 --> 2
Char. 53: 0 --> 1
Char. 73: 0 --> 2
Char. 75: 1 --> 02
Char. 76: 2 --> 0
Char. 132: 1 --> 2
Char. 140: 3 --> 5
Char. 145: 0 --> 1
Teilhardimys :
Char. 16: 0 --> 1
Char. 20: 0 --> 1
Char. 22: 1 --> 0
Char. 25: 0 --> 2
Char. 29: 0 --> 1
Char. 35: 0 --> 1
Char. 52: 2 --> 3

Char. 73: 0 --> 1
Char. 74: 0 --> 2
Char. 76: 1 --> 2
Todralestes :
Char. 9: 0 --> 1
Char. 75: 1 --> 0
Node 28 :
Char. 35: 0 --> 1
Char. 39: 0 --> 1
Char. 40: 1 --> 2
Char. 42: 0 --> 1
Char. 98: 1 --> 0
Char. 107: 2 --> 3
Char. 110: 0 --> 1
Char. 111: 0 --> 1
Char. 112: 0 --> 1
Char. 140: 0 --> 2
Node 29 :
Char. 16: 0 --> 1
Char. 33: 0 --> 1
Char. 34: 0 --> 1
Char. 85: 1 --> 0
Char. 95: 2 --> 0
Char. 97: 1 --> 0
Char. 99: 0 --> 1
Char. 108: 0 --> 1
Char. 174: 1 --> 2
Node 30 :
Char. 7: 1 --> 3
Char. 63: 0 --> 1
Char. 139: 1 --> 2
Char. 177: 0 --> 2
Node 31 :
Char. 30: 0 --> 1
Char. 84: 1 --> 2
Char. 92: 0 --> 1
Char. 104: 0 --> 1
Char. 105: 0 --> 1
Node 32 :
Char. 43: 0 --> 1
Char. 44: 1 --> 2
Char. 57: 0 --> 2
Char. 85: 0 --> 1
Char. 89: 0 --> 1
Char. 95: 0 --> 2
Node 33 :
Char. 38: 0 --> 1
Char. 97: 0 --> 1
Char. 98: 0 --> 1
Char. 107: 0 --> 1

Node 34 :
No synapomorphies
Node 35 :
Char. 40: 1 --> 0
Char. 113: 0 --> 1
Node 36 :
Char. 15: 0 --> 1
Char. 17: 0 --> 1
Char. 24: 1 --> 2
Char. 91: 1 --> 2
Node 37 :
Char. 11: 1 --> 0
Char. 29: 0 --> 1
Char. 42: 0 --> 2
Char. 76: 1 --> 2
Node 38 :
Char. 10: 1 --> 0
Char. 25: 0 --> 1
Char. 27: 0 --> 1
Char. 33: 0 --> 1
Char. 34: 0 --> 1
Char. 39: 1 --> 2
Char. 47: 1 --> 2
Node 39 :
Char. 31: 0 --> 2
Char. 63: 1 --> 0
Char. 77: 1 --> 2
Char. 109: 01 --> 2
Char. 147: 0 --> 1
Node 40 :
Char. 39: 0 --> 1
Char. 90: 0 --> 1
Char. 97: 2 --> 3
Char. 112: 0 --> 1
Char. 117: 0 --> 1
Node 41 :
Char. 47: 0 --> 1
Char. 77: 0 --> 1
Char. 80: 0 --> 1
Node 42 :
Char. 58: 0 --> 1
Char. 97: 1 --> 2
Char. 183: 0 --> 1
Node 43 :
Char. 39: 1 --> 2
Char. 88: 0 --> 1
Char. 95: 0 --> 23
Char. 105: 1 --> 2
Char. 116: 1 --> 3
Char. 140: 2 --> 3

Char. 157: 0 --> 1
Char. 162: 0 --> 1
Node 44 :
Char. 24: 1 --> 2
Char. 25: 0 --> 2
Char. 27: 0 --> 1
Char. 28: 0 --> 1
Char. 29: 0 --> 1
Char. 47: 0 --> 2
Char. 50: 0 --> 1
Char. 77: 0 --> 1
Char. 91: 0 --> 2
Char. 107: 3 --> 4
Char. 108: 1 --> 2
Char. 118: 0 --> 1
Char. 119: 0 --> 1
Char. 121: 0 --> 1
Node 45 :
Char. 131: 0 --> 1
Char. 132: 0 --> 1
Char. 180: 0 --> 2
Node 46 :
Char. 49: 0 --> 12
Char. 91: 2 --> 1
Char. 150: 0 --> 1
Char. 155: 0 --> 1
Node 47 :
Char. 3: 0 --> 1
Char. 6: 0 --> 1
Char. 7: 3 --> 4
Char. 10: 1 --> 2
Char. 20: 0 --> 1
Char. 101: 0 --> 1
Node 48 :
Char. 18: 0 --> 1
Char. 26: 0 --> 1
Char. 36: 0 --> 1
Char. 106: 0 --> 1
Char. 120: 0 --> 1
Node 49 :
Char. 3: 1 --> 2
Char. 6: 1 --> 2
Char. 7: 4 --> 5
Char. 24: 2 --> 3
Char. 32: 0 --> 1
Char. 34: 1 --> 2
Char. 35: 1 --> 0
Char. 45: 1 --> 2
Char. 46: 0 --> 12
Char. 87: 0 --> 1
Char. 90: 1 --> 3
Char. 111: 1 --> 3
Char. 140: 3 --> 4
Char. 145: 0 --> 1
Node 50 :
Char. 6: 0 --> 1
Char. 10: 1 --> 23
Char. 12: 0 --> 2
Char. 67: 0 --> 1
Char. 70: 0 --> 1
Char. 160: 1 --> 0
Node 51 :
Char. 90: 1 --> 2
Char. 116: 3 --> 1
Char. 121: 1 --> 0
Char. 165: 0 --> 1
Node 52 :
Char. 10: 1 --> 3
Char. 58: 0 --> 1
Char. 70: 0 --> 1
Char. 128: 0 --> 1

3. Analysis 3 : no characters additive and unweighted analysis

3.1 Cladogram 3 (consensus)

Traditional search, 2 trees found.

Consensus of 2 trees.

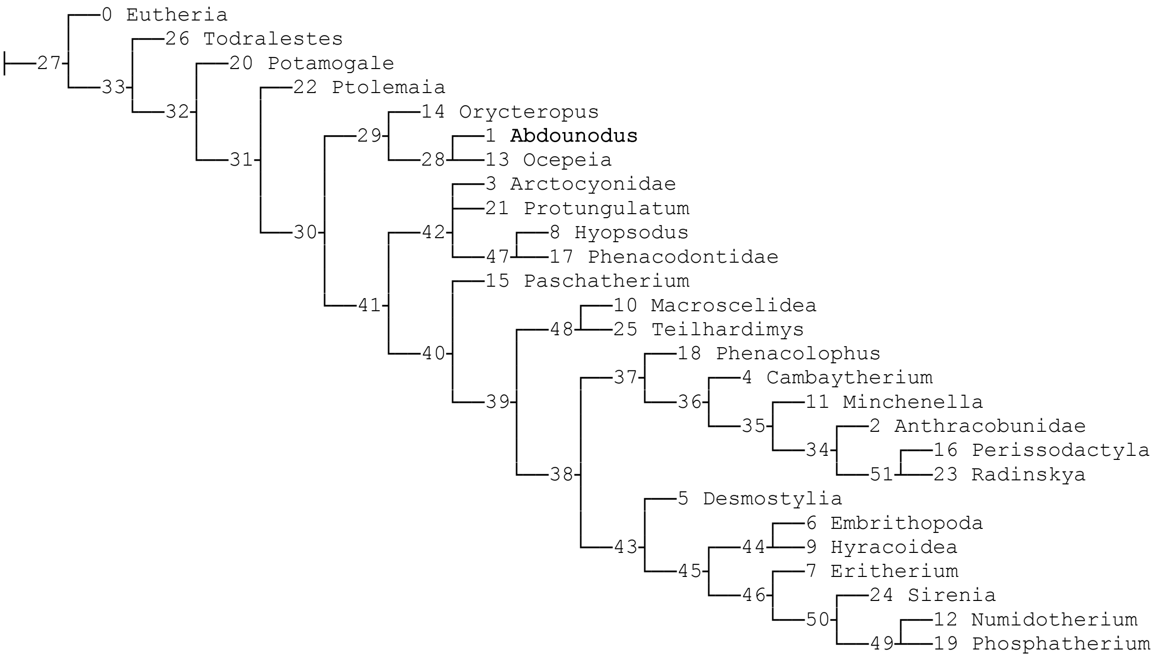

Trees length (Trees 1 and 2): 682. Retention index: 52.8. Consistency Index: 39.6.

Tree 0, homoplasy

	+0	+1	+2	+3	+4	+5	+6	+7	+8	+9
0	1	1	--	0	--	2	2	5	--	3
10	6	2	2	2	1	1	6	2	5	1
20	4	4	4	1	4	2	2	1	3	2
30	7	6	2	1	2	5	2	4	10	2
40	7	1	4	2	4	6	3	4	1	5
50	3	1	5	4	--	--	0	4	4	0
60	3	1	--	4	4	1	--	2	2	1
70	2	3	--	4	1	1	5	2	2	6
80	2	--	5	1	1	2	--	0	1	1
90	0	2	5	5	5	3	--	3	3	4
100	1	0	1	3	4	5	1	3	0	4
110	3	5	2	1	0	2	5	2	0	0
120	1	2	0	1	2	1	4	1	5	4
130	2	2	0	--	4	1	--	3	3	1
140	4	4	3	2	3	0	2	3	0	0
150	2	1	3	1	3	2	4	2	0	--
160	1	3	1	1	2	1	1	1	2	4
170	0	--	1	1	3	1	2	6	2	--
180	2	2	1	1						

Tree 1, homoplasy

	+0	+1	+2	+3	+4	+5	+6	+7	+8	+9
0	1	1	--	0	--	2	2	5	--	3
10	6	2	2	2	1	1	6	2	5	1
20	4	4	4	1	4	2	2	1	3	2
30	7	6	2	1	2	6	2	4	10	2
40	7	1	4	2	4	6	3	4	1	5
50	3	1	5	4	--	--	0	4	4	0
60	3	1	--	4	4	1	--	2	2	1
70	2	3	--	4	1	1	5	2	2	5
80	2	--	5	1	1	2	--	0	1	1
90	0	2	5	5	5	3	--	3	3	4
100	1	0	1	3	4	5	1	3	0	4
110	3	5	2	1	0	2	5	2	0	0
120	1	2	0	1	2	1	4	1	5	4
130	2	2	0	--	4	1	--	3	3	1
140	4	4	3	2	3	0	2	3	0	0
150	2	1	3	1	3	2	4	2	0	--
160	1	3	1	1	2	1	1	1	2	4
170	0	--	1	1	3	1	2	6	2	--
180	2	2	1	1						

3.2 Bremer supports (from 10000 trees, cut 2)

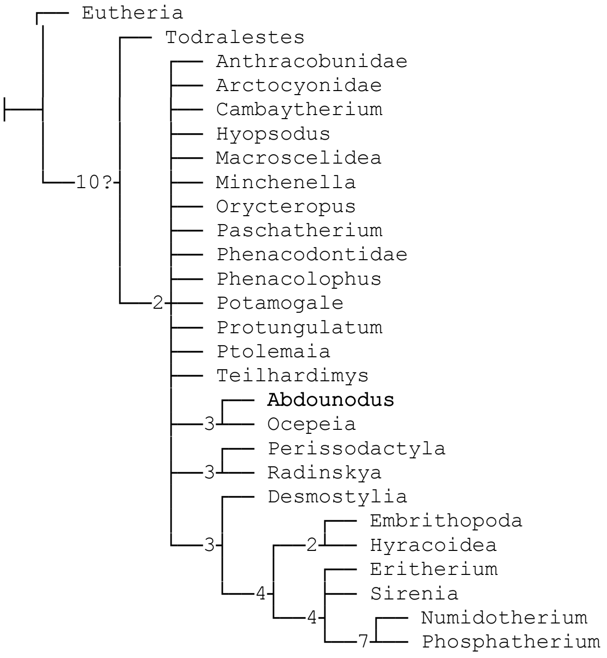

4. Analysis 4 : no characters additive and implied weighting analysis

4.1 Cladogram 4

Traditional search, 1 tree found.

Tree 0:

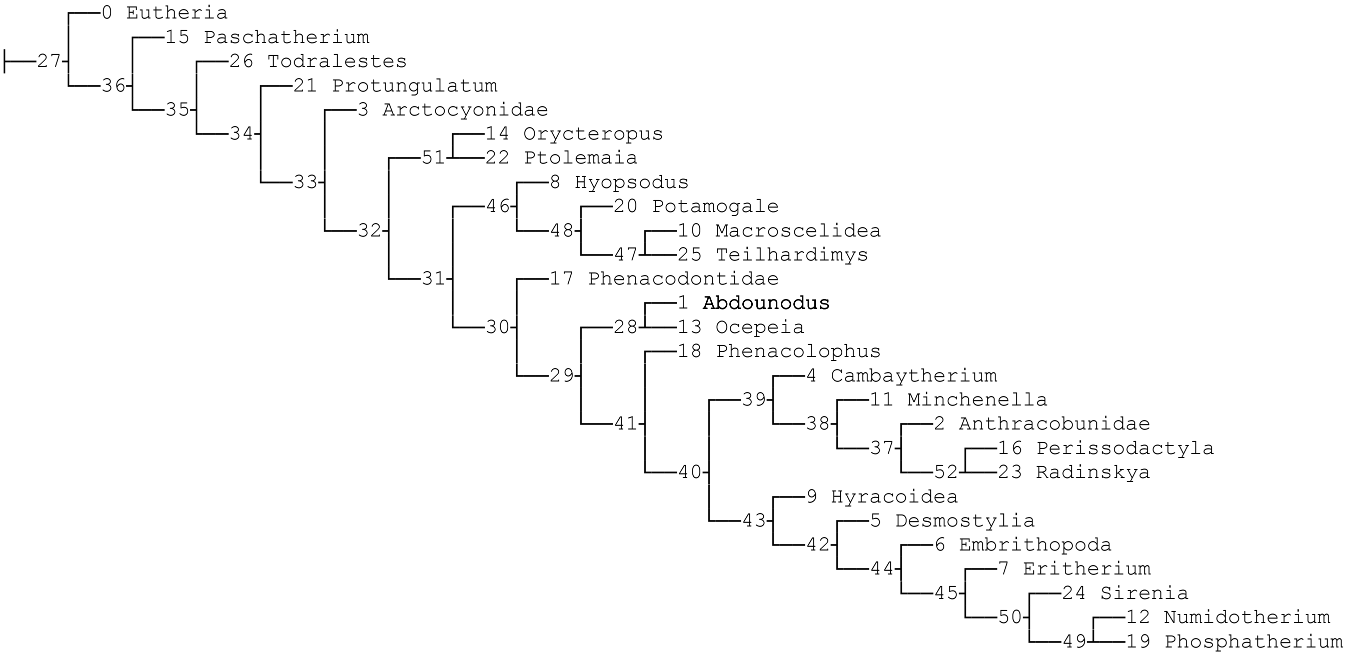

Tree length : 691. Retention index: 51.7. Consistency Index: 39.1.

5. Analysis 5 *:* Paenungulatomorpha constrained, 44 characters additive, and unweighted analysis

5.1 Cladogram 5 (consensus)

Traditional search, 4 trees found.

Tree 0:

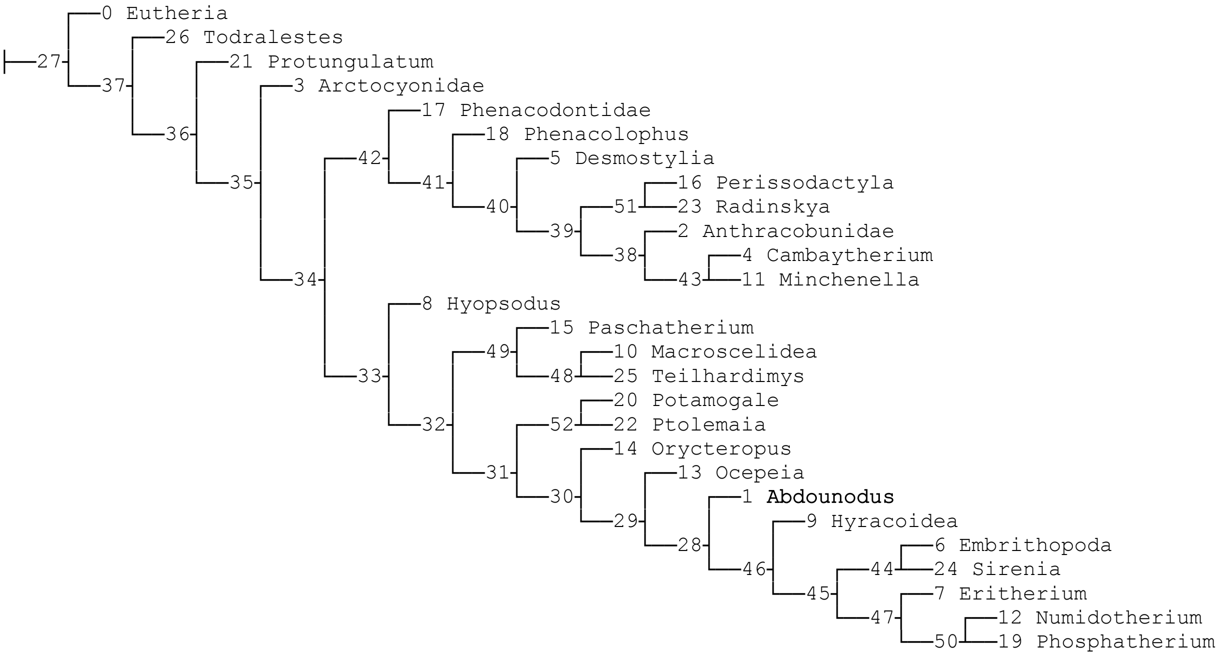

Tree 1:

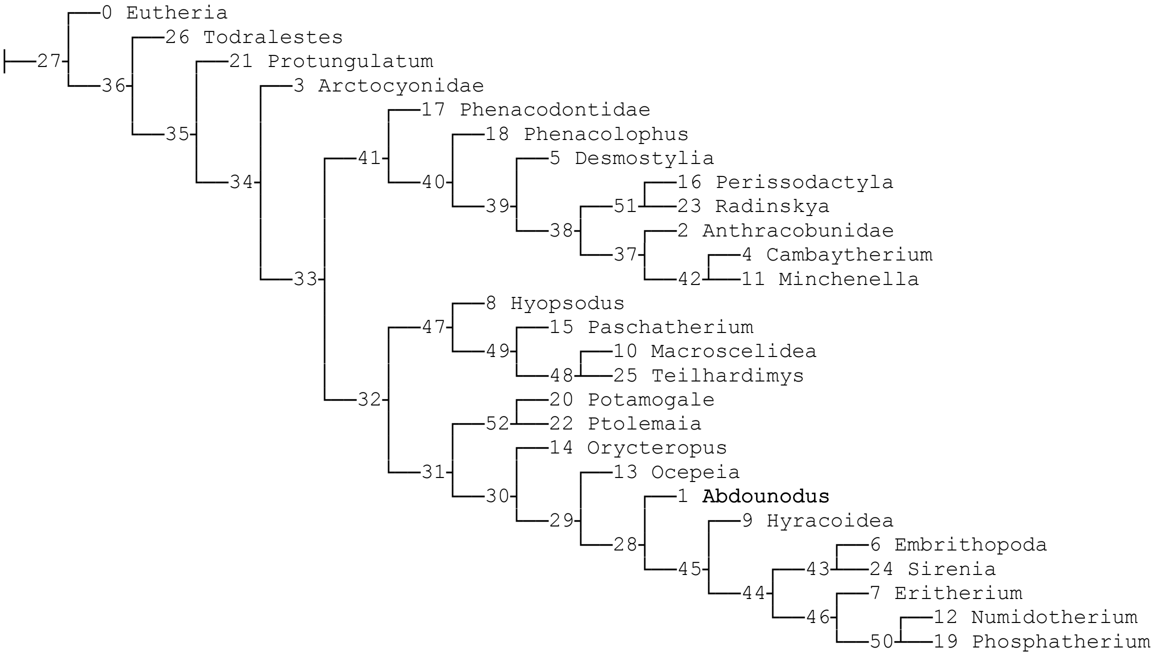

Tree 2:

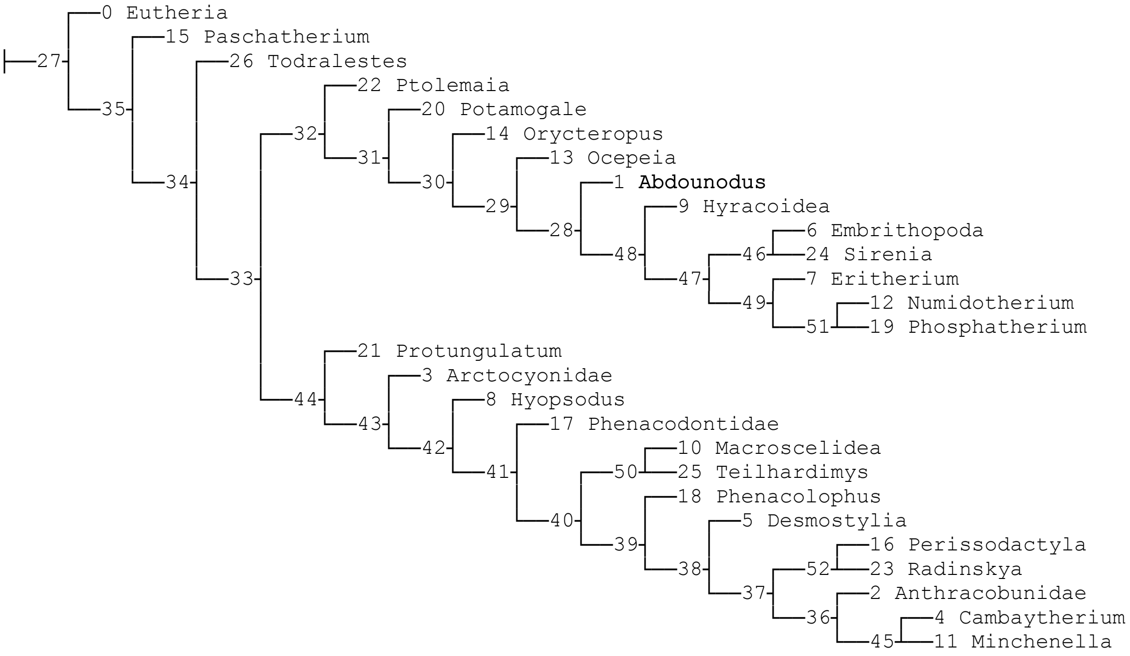
0 Eutheria
15 Paschatherium
26 Todralestes
22 Ptolemaia
20 Potamogale
14 Orycteropus
13 Ocepeia
1 **Abdounodus**
9 Hyracoidea
6 Embrithopoda
24 Sirenia
7 Eritherium
12 Numidotherium
19 Phosphatherium
21 Protungulatum
3 Arctocyonidae
8 Hyopsodus
17 Phenacodontidae
10 Macroscelidea
25 Teilhardimys
18 Phenacolophus
5 Desmostylia
16 Perissodactyla
23 Radinskya
2 Anthracobunidae
4 Cambaytherium
11 Minchenella

Tree 3:

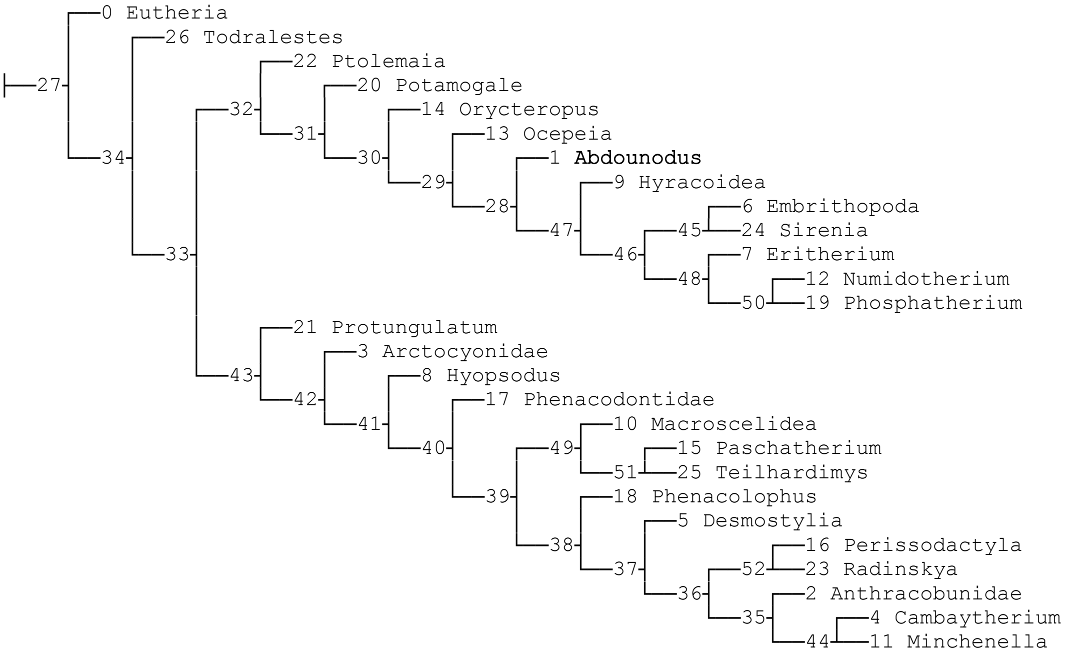
0 Eutheria
26 Todralestes
22 Ptolemaia
20 Potamogale
14 Orycteropus
13 Ocepeia
1 **Abdounodus**
9 Hyracoidea
6 Embrithopoda
24 Sirenia
7 Eritherium
12 Numidotherium
19 Phosphatherium
21 Protungulatum
3 Arctocyonidae
8 Hyopsodus
17 Phenacodontidae
10 Macroscelidea
15 Paschatherium
25 Teilhardimys
18 Phenacolophus
5 Desmostylia
16 Perissodactyla
23 Radinskya
2 Anthracobunidae
4 Cambaytherium
11 Minchenella

Tree 0, homoplasy

	+0	+1	+2	+3	+4	+5	+6	+7	+8	+9
0	2	2	--	0	--	1	3	8	--	4
10	10	2	6	3	1	3	6	3	4	1
20	3	6	5	0	5	3	2	2	4	3
30	7	7	2	1	2	5	2	3	11	3
40	7	2	6	3	6	6	4	5	2	6
50	4	1	6	4	--	--	1	7	2	0
60	3	0	--	2	3	1	--	4	2	2
70	2	2	--	4	3	1	7	2	2	6
80	1	--	6	1	1	2	--	0	1	2
90	2	4	6	6	5	4	--	3	2	3
100	1	0	1	2	4	7	1	3	0	4
110	2	4	2	1	1	2	5	3	1	0
120	1	4	1	1	3	1	5	1	3	4
130	2	1	0	--	4	1	--	5	3	1
140	4	4	3	4	3	1	4	1	1	1
150	1	1	2	1	3	1	2	2	0	--
160	2	3	1	2	4	1	1	1	2	5
170	0	--	3	2	2	1	1	5	2	--
180	2	3	2	1						

Tree 1, homoplasy

	+0	+1	+2	+3	+4	+5	+6	+7	+8	+9
0	2	2	--	0	--	1	3	8	--	4
10	10	2	6	3	1	3	7	3	4	1
20	3	6	5	0	5	3	2	2	3	3
30	7	7	2	1	2	5	2	3	11	3
40	7	2	6	3	6	6	4	5	2	6
50	4	1	6	4	--	--	1	7	2	0
60	3	0	--	2	4	1	--	4	2	2
70	2	2	--	4	3	1	7	2	2	6
80	1	--	6	1	1	2	--	0	1	2
90	2	4	6	6	5	4	--	3	2	3
100	1	0	1	2	4	7	1	3	0	4
110	2	4	2	1	1	2	5	3	1	0
120	1	4	1	1	3	1	5	1	4	4
130	2	1	0	--	4	1	--	5	3	1
140	4	4	3	4	3	1	4	1	1	1
150	1	1	2	1	3	1	2	2	0	--
160	2	3	1	2	4	1	1	1	2	4
170	0	--	3	2	2	1	1	5	2	--
180	2	3	2	0						

Tree 2, homoplasy

	+0	+1	+2	+3	+4	+5	+6	+7	+8	+9
0	2	2	--	0	--	2	3	11	--	4
10	9	1	6	3	1	3	6	3	4	1
20	3	5	4	0	4	3	2	2	4	3
30	7	7	2	1	2	5	2	3	11	2
40	7	2	6	3	7	6	4	5	2	6
50	4	1	5	4	--	--	1	7	3	0
60	3	0	--	3	5	1	--	4	2	2
70	2	2	--	4	3	1	7	3	2	6
80	1	--	6	1	2	2	--	0	1	1
90	1	3	5	5	5	4	--	2	2	3
100	1	0	1	2	5	8	1	4	0	4
110	2	4	1	1	1	1	6	2	1	0
120	1	3	1	1	3	1	4	1	4	4
130	2	1	0	--	4	1	--	6	3	1
140	4	4	3	3	3	1	3	2	1	1
150	1	1	3	1	3	1	4	2	0	--
160	2	3	1	2	4	1	1	1	2	4
170	0	--	3	2	2	1	2	5	2	--
180	2	3	2	0						

Tree 3, homoplasy

	+0	+1	+2	+3	+4	+5	+6	+7	+8	+9
0	2	2	--	0	--	2	3	9	--	4
10	10	2	6	3	1	3	5	3	5	1
20	3	5	4	0	5	3	3	2	3	2
30	6	7	2	1	2	5	3	3	11	3
40	7	2	6	2	6	6	4	4	2	6
50	4	1	5	4	--	--	1	7	3	0
60	3	0	--	3	5	1	--	4	2	2
70	2	2	--	4	3	1	7	2	2	6
80	0	--	5	1	1	1	--	0	1	2
90	2	4	6	6	4	3	--	2	2	3
100	1	0	1	2	4	7	2	4	0	4
110	2	5	2	1	1	2	5	3	1	0
120	2	4	1	1	3	1	4	1	4	4
130	2	1	0	--	4	1	--	6	3	1
140	4	4	3	3	3	1	3	2	1	1
150	1	1	3	1	3	1	4	2	0	--
160	2	3	1	2	4	1	1	1	2	4
170	0	--	3	2	2	1	2	5	2	--
180	2	3	2	0						

Strict consensus of 4 trees (cladogram 5)

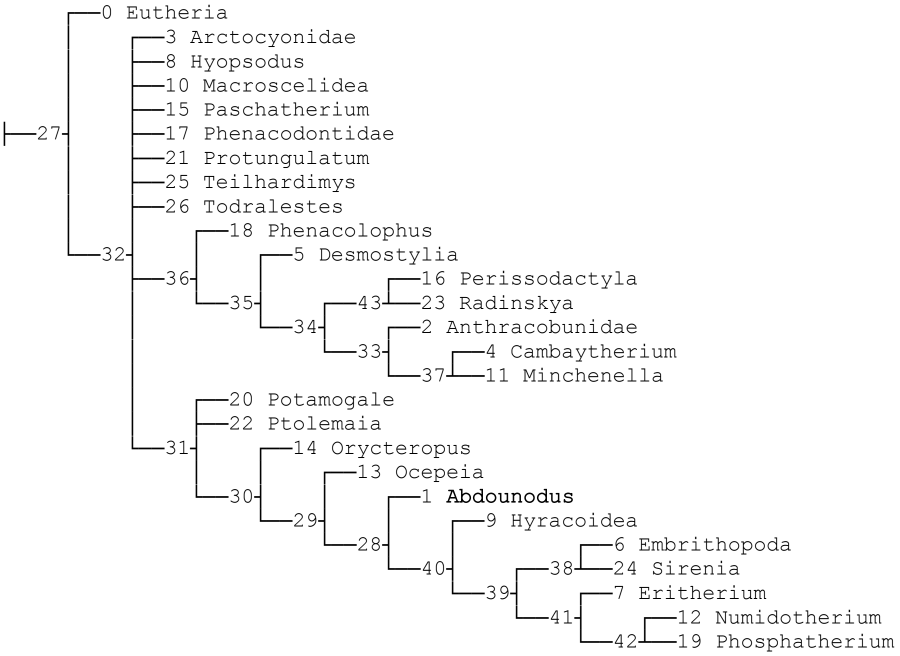

Node 29 = **Paenungulatomorpha**

Trees length : 744. Retention index: 52.2. Consistency Index: 36.6.

5.2 Bremer supports (from 10000 trees, cut 2)

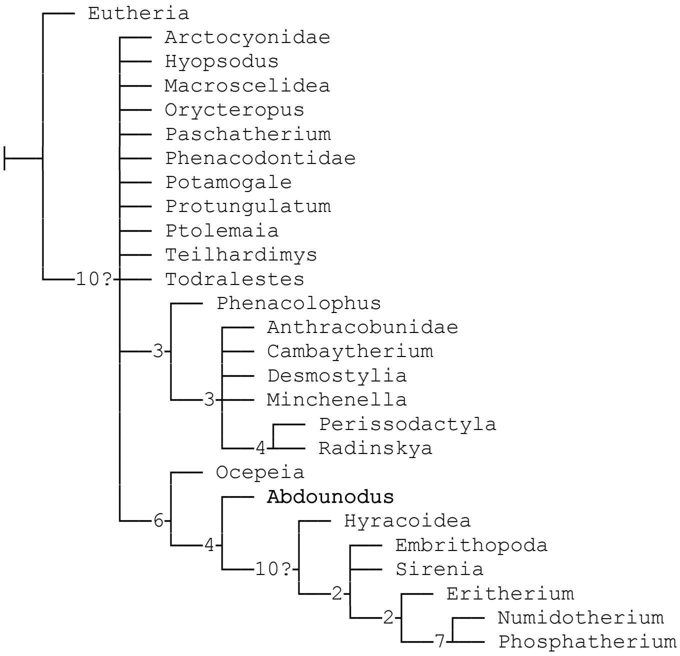

6. Analysis 6 : clade Paenungulatomorpha constrained, 44 characters additive, and implied weighting analysis

6.1 Cladogram 6

Traditional search, 1 tree found.

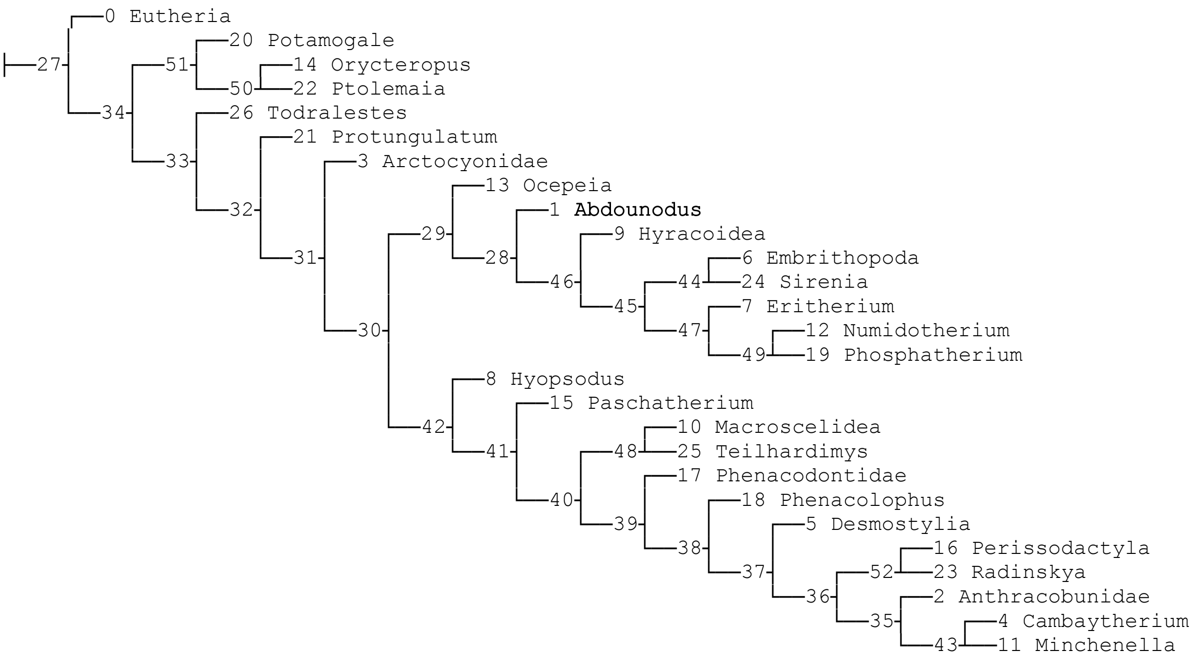

Node 29 = **Paenungulatomorpha**
Trees length : 750. Retention index: 51.6. Consistency Index: 36.3.

Cladogram 6, homoplasy

	+0	+1	+2	+3	+4	+5	+6	+7	+8	+9
0	2	2	--	0	--	2	3	12	--	5
10	10	2	7	3	1	3	8	3	5	1
20	3	6	5	0	6	3	1	2	4	3
30	6	7	2	1	2	5	2	3	10	2
40	7	2	6	3	6	6	4	3	0	6
50	4	1	6	4	--	--	1	7	2	0
60	3	0	--	3	6	1	--	4	3	2
70	2	2	--	4	3	1	7	2	2	7
80	0	--	6	1	0	2	--	0	1	2
90	1	4	6	6	5	4	--	1	1	3
100	1	0	1	2	4	7	1	2	0	7
110	2	4	1	1	1	3	7	2	1	0
120	1	4	1	1	2	1	4	1	3	4
130	3	1	0	--	4	1	--	6	3	0
140	4	4	4	3	3	1	4	1	1	1
150	1	1	3	1	3	1	3	2	0	--
160	2	3	1	2	4	1	1	1	2	4
170	0	--	3	2	3	1	2	4	2	--
180	2	3	2	0						

6.2. Synapomorphies of cladogram 6

Eutheria :
No autapomorphies
Abdounodus :
Char. 38: 1 --> 2
Char. 41: 0 --> 1
Char. 42: 1 --> 2
Char. 44: 2 --> 0
Char. 57: 2 --> 1
Char. 93: 0 --> 1
Anthracobunidae :
Char. 25: 1 --> 2
Char. 57: 2 --> 1
Char. 141: 0 --> 1
Char. 147: 1 --> 0
Char. 173: 0 --> 1
Arctocyonidae :
Char. 155: 0 --> 1
Cambaytherium :
Char. 15: 1 --> 0
Char. 17: 1 --> 0
Char. 20: 0 --> 1
Char. 21: 1 --> 0
Char. 22: 1 --> 0
Char. 24: 2 --> 1
Char. 38: 0 --> 2
Char. 91: 2 --> 1
Desmostylia :
Char. 0: 0 --> 1
Char. 1: 0 --> 1
Char. 7: 1 --> 0
Char. 16: 0 --> 1
Char. 25: 1 --> 2
Char. 31: 2 --> 0
Char. 57: 2 --> 1
Char. 60: 1 --> 2
Char. 82: 3 --> 0
Char. 88: 0 --> 1
Char. 102: 0 --> 1
Char. 105: 1 --> 2
Char. 126: 2 --> 0
Char. 128: 0 --> 1
Char. 130: 0 --> 1
Char. 137: 1 --> 23
Char. 144: 0 --> 12
Char. 157: 0 --> 1
Char. 162: 0 --> 1
Embrithopoda :
Char. 24: 2 --> 3
Char. 26: 0 --> 1
Char. 31: 0 --> 1
Char. 32: 0 --> 1
Char. 34: 1 --> 2
Char. 38: 1 --> 0
Char. 42: 1 --> 0
Char. 92: 1 --> 0
Char. 95: 23 --> 1
Char. 105: 12 --> 0
Char. 106: 0 --> 2
Char. 143: 0 --> 1
Char. 178: 0 --> 1
Eritherium :
Char. 12: 01 --> 2
Char. 31: 0 --> 1
Char. 41: 01 --> 2
Char. 42: 1 --> 2
Char. 44: 2 --> 01
Char. 47: 2 --> 1
Hyopsodus :
Char. 5: 0 --> 1
Char. 10: 1 --> 2
Char. 35: 0 --> 1
Char. 57: 2 --> 1
Char. 68: 0 --> 1
Char. 124: 0 --> 2
Char. 138: 1 --> 0
Char. 157: 0 --> 1
Char. 164: 0 --> 1
Char. 168: 0 --> 1
Char. 173: 0 --> 1
Hyracoidea :
Char. 30: 1 --> 0
Char. 31: 0 --> 2
Char. 43: 0 --> 1
Char. 49: 1 --> 0
Char. 52: 2 --> 0
Char. 73: 0 --> 1
Char. 74: 0 --> 2
Char. 92: 1 --> 0
Char. 111: 1 --> 2
Char. 138: 1 --> 0
Char. 154: 0 --> 12
Macroscelidea :
Char. 17: 0 --> 1
Char. 31: 0 --> 1
Char. 43: 1 --> 0
Char. 45: 1 --> 0
Char. 49: 0 --> 1
Char. 78: 0 --> 1
Char. 85: 1 --> 0
Char. 105: 1 --> 2
Minchenella :
Char. 45: 1 --> 0
Char. 53: 0 --> 1
Char. 92: 1 --> 0
Char. 93: 1 --> 0
Char. 104: 1 --> 0
Char. 105: 1 --> 0
Char. 109: 2 --> 1
Char. 110: 1 --> 0
Char. 111: 1 --> 2
Numidotherium :
Char. 9: 0 --> 3
Char. 10: 2 --> 3
Char. 21: 0 --> 1
Char. 37: 1 --> 0
Char. 42: 1 --> 0
Char. 51: 0 --> 3
Char. 52: 2 --> 1
Char. 53: 0 --> 1
Char. 67: 0 --> 2
Char. 70: 0 --> 1
Char. 73: 0 --> 1
Char. 74: 0 --> 1
Char. 99: 1 --> 0
Char. 102: 0 --> 1
Char. 122: 0 --> 1
Char. 125: 0 --> 1
Char. 128: 0 --> 1
Char. 131: 0 --> 1
Char. 137: 2 --> 1
Char. 141: 1 --> 2
Char. 144: 0 --> 1
Char. 154: 0 --> 1
Char. 156: 0 --> 1
Char. 163: 1 --> 2
Char. 168: 0 --> 1
Char. 178: 0 --> 1
Ocepeia :
Char. 6: 0 --> 1
Char. 10: 1 --> 3
Char. 12: 01 --> 3
Char. 20: 0 --> 1
Char. 31: 0 --> 2
Char. 36: 0 --> 1
Char. 46: 0 --> 1
Char. 49: 1 --> 2
Char. 51: 0 --> 2
Char. 52: 2 --> 3
Char. 67: 0 --> 1
Char. 70: 0 --> 1
Char. 92: 1 --> 0
Char. 104: 1 --> 0
Char. 105: 1 --> 0
Char. 116: 1 --> 3
Char. 122: 0 --> 2
Char. 124: 0 --> 3
Char. 134: 1 --> 0
Char. 137: 1 --> 0
Char. 153: 0 --> 1
Char. 167: 0 --> 1
Char. 168: 0 --> 1
Char. 175: 0 --> 1
Char. 180: 0 --> 1
Orycteropus :
Char. 7: 123 --> 5
Char. 9: 0 --> 12
Char. 12: 0 --> 2
Char. 13: 0 --> 1
Char. 14: 0 --> 1
Char. 19: 0 --> 1
Char. 44: 1 --> 2
Char. 56: 0 --> 2
Char. 57: 0 --> 2
Char. 60: 0 --> 1
Char. 67: 0 --> 2
Char. 68: 01 --> 2
Char. 73: 0 --> 2
Char. 75: 1 --> 2
Char. 121: 0 --> 1
Char. 123: 0 --> 1
Char. 125: 0 --> 1
Char. 130: 0 --> 1
Char. 139: 1 --> 0
Char. 142: 1 --> 0
Char. 143: 0 --> 2
Char. 164: 0 --> 2
Paschatherium :
Char. 10: 1 --> 0

Char. 11: 1 --> 0
Char. 16: 0 --> 1
Char. 22: 1 --> 0
Char. 24: 1 --> 0
Char. 29: 0 --> 1
Char. 38: 01 --> 2
Char. 52: 2 --> 3
Char. 89: 1 --> 0
Char. 92: 1 --> 0
Char. 115: 1 --> 0
Perissodactyla :
Char. 83: 0 --> 1
Char. 123: 0 --> 1
Char. 134: 1 --> 0
Char. 137: 1 --> 0
Char. 138: 1 --> 0
Char. 161: 0 --> 1
Phenacodontidae :
Char. 10: 1 --> 2
Char. 37: 0 --> 1
Char. 83: 0 --> 1
Char. 103: 0 --> 1
Char. 124: 0 --> 2
Char. 173: 0 --> 1
Phenacolophus :
Char. 10: 1 --> 0
Char. 32: 0 --> 1
Char. 45: 1 --> 2
Char. 73: 0 --> 2
Char. 76: 1 --> 0
Char. 77: 2 --> 0
Char. 103: 0 --> 2
Char. 104: 1 --> 0
Char. 115: 1 --> 0
Phosphatherium :
Char. 57: 2 --> 1
Char. 120: 0 --> 2
Char. 124: 0 --> 2
Char. 172: 1 --> 0
Potamogale :
Char. 49: 0 --> 2
Char. 63: 0 --> 1
Char. 64: 1 --> 2
Char. 94: 0 --> 1
Char. 106: 0 --> 1
Char. 126: 0 --> 2
Char. 129: 0 --> 1
Char. 137: 12 --> 3
Char. 139: 1 --> 3
Char. 140: 0 --> 12
Char. 141: 0 --> 2
Char. 144: 0 --> 2
Char. 153: 0 --> 1
Char. 165: 0 --> 1
Char. 177: 0 --> 1
Char. 178: 0 --> 1
Protungulatum :
Char. 27: 0 --> 1
Char. 35: 0 --> 1
Char. 46: 0 --> 2
Ptolemaia :
Char. 7: 123 --> 0
Char. 51: 0 --> 1
Char. 115: 1 --> 2
Char. 116: 1 --> 2
Radinskya :
Char. 82: 3 --> 1
Char. 143: 1 --> 0
Sirenia :
Char. 14: 0 --> 1
Char. 19: 0 --> 1
Char. 30: 1 --> 0
Char. 42: 1 --> 2
Char. 46: 0 --> 2
Char. 53: 0 --> 1
Char. 73: 0 --> 2
Char. 75: 1 --> 02
Char. 76: 2 --> 0
Char. 93: 0 --> 2
Char. 132: 1 --> 2
Char. 140: 3 --> 5
Char. 141: 1 --> 0
Char. 145: 0 --> 1
Teilhardimys :
Char. 16: 0 --> 1
Char. 20: 0 --> 1
Char. 22: 1 --> 0
Char. 25: 0 --> 2
Char. 29: 0 --> 1
Char. 35: 0 --> 1
Char. 52: 2 --> 3
Char. 73: 0 --> 1
Char. 74: 0 --> 2
Char. 76: 1 --> 2
Todralestes :
Char. 9: 0 --> 1
Char. 51: 0 --> 2
Char. 75: 1 --> 0
Node 28 :
Char. 35: 0 --> 1
Char. 39: 0 --> 1
Char. 40: 1 --> 2
Char. 42: 0 --> 1
Char. 98: 1 --> 0
Char. 107: 2 --> 3
Char. 110: 0 --> 1
Char. 111: 0 --> 1
Char. 112: 0 --> 1
Char. 140: 0 --> 2
Node 29 :
Char. 33: 0 --> 1
Char. 34: 0 --> 1
Char. 37: 0 --> 1
Char. 43: 1 --> 0
Char. 49: 0 --> 1
Char. 61: 0 --> 1
Char. 85: 1 --> 0
Char. 95: 2 --> 0
Char. 97: 1 --> 0
Char. 99: 0 --> 1
Char. 108: 0 --> 1
Char. 141: 0 --> 1
Char. 166: 0 --> 2
Node 30 :
Char. 7: 1 --> 23
Char. 63: 0 --> 1
Char. 139: 1 --> 2
Char. 177: 0 --> 2
Node 31 :
Char. 30: 0 --> 1
Char. 84: 1 --> 2
Char. 92: 0 --> 1
Char. 104: 0 --> 1
Char. 105: 0 --> 1
Node 32 :
Char. 43: 0 --> 1
Char. 44: 1 --> 2
Char. 57: 0 --> 2
Char. 85: 0 --> 1
Char. 89: 0 --> 1
Char. 95: 0 --> 2
Node 33 :
Char. 38: 0 --> 1
Char. 97: 0 --> 1
Char. 98: 0 --> 1
Char. 107: 0 --> 1
Node 34 :
No synapomorphies
Node 35 :
Char. 10: 1 --> 0
Char. 42: 0 --> 2
Node 36 :
Char. 15: 0 --> 1
Char. 17: 0 --> 1
Char. 135: 0 --> 1
Node 37 :
Char. 29: 0 --> 1
Char. 35: 0 --> 1
Char. 76: 1 --> 2
Char. 110: 0 --> 1
Char. 113: 0 --> 1
Node 38 :
Char. 25: 0 --> 1
Char. 27: 0 --> 1
Char. 33: 0 --> 1
Char. 34: 0 --> 1
Char. 39: 1 --> 2
Char. 47: 1 --> 2
Node 39 :
Char. 31: 0 --> 2
Char. 64: 1 --> 0
Char. 77: 1 --> 2
Char. 109: 01 --> 2
Char. 147: 0 --> 1
Node 40 :
Char. 39: 0 --> 1
Char. 90: 0 --> 1
Char. 97: 2 --> 3
Char. 112: 0 --> 1
Char. 117: 0 --> 1
Node 41 :
Char. 47: 0 --> 1
Char. 77: 0 --> 1
Char. 80: 0 --> 1
Node 42 :
Char. 58: 0 --> 1
Char. 97: 1 --> 2
Char. 169: 1 --> 0
Char. 183: 0 --> 1
Node 43 :
Char. 16: 0 --> 1
Char. 44: 2 --> 0
Char. 113: 1 --> 0
Node 44 :
Char. 37: 1 --> 0
Char. 60: 1 --> 2
Char. 131: 0 --> 1
Char. 132: 0 --> 1
Char. 180: 0 --> 2
Node 45 :

Char. 39: 1 --> 2
Char. 88: 0 --> 1
Char. 95: 0 --> 23
Char. 140: 2 --> 3
Char. 155: 0 --> 1
Char. 157: 0 --> 1
Char. 162: 0 --> 1
Node 46 :
Char. 24: 1 --> 2
Char. 25: 0 --> 2
Char. 27: 0 --> 1
Char. 28: 0 --> 1
Char. 29: 0 --> 1
Char. 47: 0 --> 2
Char. 77: 0 --> 1
Char. 82: 0 --> 2
Char. 107: 3 --> 4
Char. 108: 1 --> 2
Char. 118: 0 --> 1
Char. 119: 0 --> 1
Char. 121: 0 --> 1
Node 47 :
Char. 3: 0 --> 1
Char. 6: 0 --> 1
Char. 7: 3 --> 4
Char. 10: 1 --> 2
Char. 20: 0 --> 1
Char. 82: 2 --> 1
Char. 93: 0 --> 1
Char. 101: 0 --> 1
Node 48 :
Char. 18: 0 --> 1
Char. 26: 0 --> 1
Char. 36: 0 --> 1
Char. 106: 0 --> 1
Char. 120: 0 --> 1
Node 49 :
Char. 3: 1 --> 2
Char. 6: 1 --> 2
Char. 7: 4 --> 5
Char. 24: 2 --> 3
Char. 32: 0 --> 1
Char. 34: 1 --> 2
Char. 35: 1 --> 0
Char. 45: 1 --> 2
Char. 46: 0 --> 12
Char. 87: 0 --> 1
Char. 90: 1 --> 3
Char. 111: 1 --> 3
Char. 140: 3 --> 4
Char. 145: 0 --> 1
Node 50 :
Char. 26: 0 --> 2
Char. 47: 0 --> 1
Char. 48: 0 --> 2
Char. 89: 0 --> 1
Node 51 :
Char. 10: 1 --> 3
Char. 58: 0 --> 1
Char. 70: 0 --> 1
Char. 124: 0 --> 1
Char. 128: 0 --> 1
Char. 156: 0 --> 1
Node 52 :
Char. 90: 1 --> 2
Char. 111: 1 --> 2
Char. 116: 3 --> 1
Char. 121: 1 --> 0
Char. 165: 0 --> 1

7. Analysis 7 *:* Hypocone (character 98) X 5, 44 characters additive, and unweighted analysis

7.1 Cladogram 7

Traditional search, 8 trees found.

Strict consensus of 8 trees

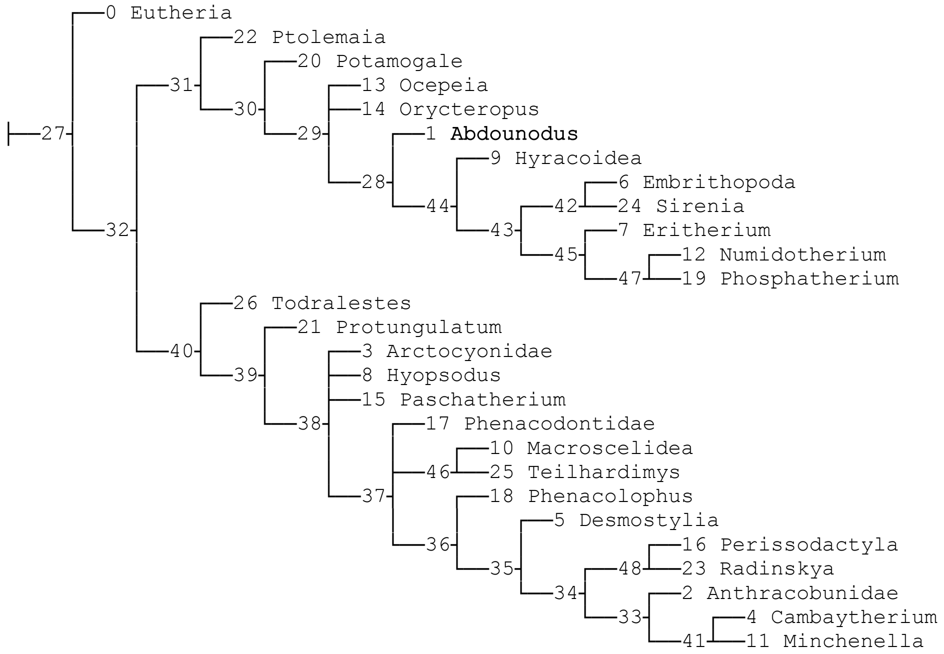

Node 29 = **Paenungulatomorpha**

Trees length : 760. Retention index: 56.7. Consistency Index: 37.4.

7.2 Bremer supports (from 10000 trees, cut 2)

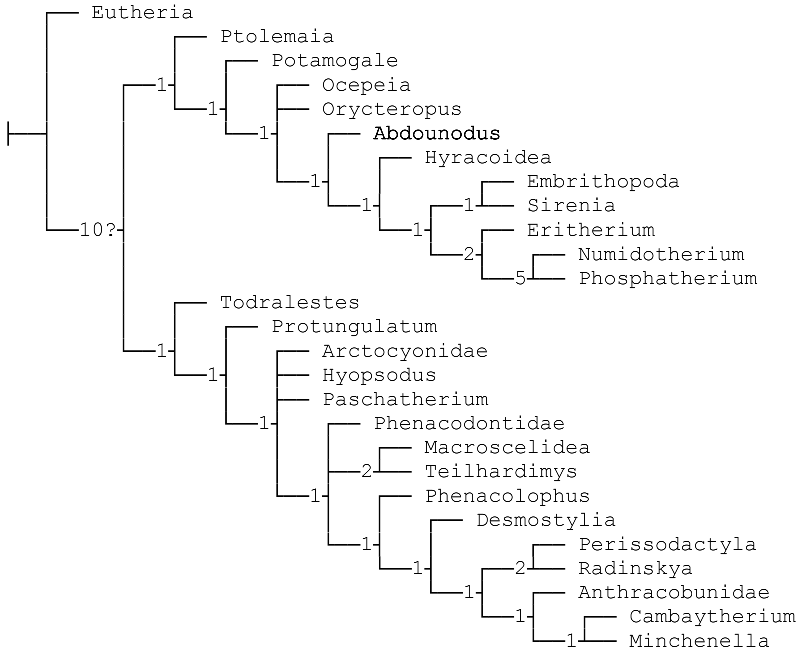

7.3 Synapomorphies common to 8 trees
(Node numbers refer to nodes in consensus)

Eutheria :
All trees:
No autapomorphies:

Abdounodus :
All trees:
Char. 18: 1 --> 0
Char. 38: 1 --> 2
Char. 41: 0 --> 1
Char. 42: 1 --> 2
Char. 44: 12 --> 0
Char. 93: 0 --> 1
Some trees:
Char. 57: 2 --> 1
Char. 126: 0 --> 1

Anthracobunidae :
All trees:
Char. 25: 1 --> 2
Char. 141: 0 --> 1
Char. 147: 1 --> 0
Char. 173: 0 --> 1
Some trees:
Char. 57: 2 --> 1

Arctocyonidae :
All trees:
Char. 155: 0 --> 1
Char. 161: 0 --> 1
Some trees:
Char. 31: 02 --> 1
Char. 45: 1 --> 2
Char. 176: 1 --> 0
Char. 177: 2 --> 0

Cambaytherium :
All trees:
Char. 15: 1 --> 0
Char. 17: 1 --> 0
Char. 20: 0 --> 1
Char. 21: 1 --> 0
Char. 22: 1 --> 0
Char. 24: 2 --> 1
Char. 38: 0 --> 2

Char. 91: 2 --> 1

Desmostylia :
All trees:
Char. 0: 0 --> 1
Char. 1: 0 --> 1
Char. 7: 1 --> 0
Char. 16: 0 --> 1
Char. 25: 1 --> 2
Char. 60: 1 --> 2
Char. 88: 0 --> 1
Char. 102: 0 --> 1
Char. 105: 1 --> 2
Char. 130: 0 --> 1
Char. 137: 1 --> 23
Char. 144: 0 --> 12
Char. 146: 0 --> 2
Char. 157: 0 --> 1
Char. 162: 0 --> 1
Some trees:
Char. 21: 1 --> 0
Char. 31: 2 --> 0
Char. 57: 2 --> 1
Char. 63: 0 --> 1
Char. 82: 3 --> 0
Char. 126: 2 --> 0
Char. 127: 1 --> 2
Char. 128: 0 --> 1

Embrithopoda :
All trees:
Char. 24: 2 --> 3
Char. 26: 0 --> 1
Char. 31: 0 --> 1
Char. 32: 0 --> 1
Char. 34: 1 --> 2
Char. 38: 1 --> 0
Char. 42: 1 --> 0
Char. 95: 23 --> 1
Char. 105: 12 --> 0
Char. 106: 0 --> 2
Char. 143: 0 --> 1
Char. 178: 0 --> 1
Some trees:
Char. 12: 1 --> 0
Char. 182: 0 --> 1

Eritherium :
All trees:
Char. 12: 01 --> 2
Char. 31: 0 --> 1
Char. 41: 01 --> 2
Char. 42: 1 --> 2
Char. 47: 2 --> 1
Some trees:
Char. 44: 2 --> 01

Hyopsodus :
All trees:
Char. 5: 0 --> 1
Char. 12: 0 --> 1
Char. 68: 0 --> 1
Char. 138: 1 --> 0
Char. 152: 0 --> 1
Char. 156: 0 --> 1
Char. 157: 0 --> 1
Char. 164: 0 --> 1
Char. 168: 0 --> 1
Some trees:
Char. 10: 1 --> 2
Char. 21: 0 --> 1
Char. 28: 0 --> 1
Char. 35: 0 --> 1
Char. 38: 1 --> 0
Char. 63: 0 --> 1
Char. 64: 0 --> 1
Char. 93: 0 --> 1
Char. 107: 1 --> 2
Char. 124: 0 --> 2
Char. 127: 1 --> 0
Char. 169: 1 --> 0

Hyracoidea :
All trees:
Char. 30: 1 --> 0
Char. 31: 0 --> 2
Char. 43: 0 --> 1
Char. 49: 1 --> 0
Char. 52: 2 --> 0
Char. 73: 0 --> 1
Char. 74: 0 --> 2
Char. 111: 1 --> 2
Char. 138: 1 --> 0
Char. 154: 0 --> 12
Some trees:
Char. 12: 1 --> 0
Char. 130: 1 --> 0
Char. 182: 0 --> 1

Macroscelidea :
All trees:
Char. 17: 0 --> 1
Char. 43: 1 --> 0
Char. 45: 12 --> 0
Char. 49: 0 --> 1
Char. 78: 0 --> 1
Char. 85: 1 --> 0
Char. 105: 1 --> 2
Some trees:
Char. 31: 0 --> 1

Minchenella :
All trees:
Char. 53: 0 --> 1
Char. 92: 1 --> 0
Char. 93: 1 --> 0
Char. 104: 1 --> 0
Char. 105: 1 --> 0
Char. 109: 2 --> 1
Char. 110: 1 --> 0
Char. 111: 1 --> 2
Some trees:
Char. 45: 1 --> 0

Numidotherium :
All trees:
Char. 9: 0 --> 3
Char. 10: 2 --> 3
Char. 21: 0 --> 1
Char. 37: 1 --> 0
Char. 42: 1 --> 0
Char. 51: 0 --> 3
Char. 52: 2 --> 1
Char. 53: 0 --> 1
Char. 67: 0 --> 2
Char. 70: 0 --> 1
Char. 73: 0 --> 1
Char. 74: 0 --> 1
Char. 99: 1 --> 0
Char. 102: 0 --> 1
Char. 122: 0 --> 1
Char. 125: 0 --> 1
Char. 128: 0 --> 1
Char. 131: 0 --> 1
Char. 137: 2 --> 1
Char. 141: 1 --> 2
Char. 144: 0 --> 1
Char. 154: 0 --> 1
Char. 156: 0 --> 1
Char. 163: 1 --> 2
Char. 168: 0 --> 1
Char. 178: 0 --> 1
Some trees:

Char. 12: 1 --> 0

Ocepeia :
All trees:
Char. 7: 3 --> 12
Char. 12: 12 --> 3
Char. 46: 0 --> 1
Char. 49: 1 --> 2
Char. 51: 0 --> 2
Char. 68: 1 --> 0
Char. 116: 1 --> 3
Char. 122: 0 --> 2
Char. 134: 1 --> 0
Char. 137: 12 --> 0
Char. 146: 0 --> 1
Char. 153: 0 --> 1
Char. 167: 0 --> 1
Char. 168: 0 --> 1
Char. 174: 2 --> 0
Char. 175: 0 --> 1
Some trees:
Char. 20: 0 --> 1
Char. 31: 0 --> 2
Char. 36: 0 --> 1
Char. 50: 0 --> 1
Char. 98: 0 --> 1
Char. 126: 0 --> 1
Char. 150: 0 --> 1
Char. 180: 0 --> 1
Char. 182: 0 --> 1

Orycteropus :
All trees:
Char. 6: 1 --> 2
Char. 7: 3 --> 5
Char. 14: 0 --> 1
Char. 19: 0 --> 1
Char. 26: 0 --> 2
Char. 47: 0 --> 1
Char. 48: 0 --> 2
Char. 56: 0 --> 2
Char. 67: 01 --> 2
Char. 68: 1 --> 2
Char. 75: 1 --> 2
Char. 120: 0 --> 3
Char. 121: 0 --> 1
Char. 123: 0 --> 1
Char. 125: 0 --> 1
Char. 127: 1 --> 4
Char. 143: 0 --> 2
Char. 164: 0 --> 2
Char. 169: 1 --> 0
Char. 172: 01 --> 2
Some trees:
Char. 49: 1 --> 0
Char. 73: 0 --> 2
Char. 139: 2 --> 0
Char. 141: 1 --> 0
Char. 152: 0 --> 1
Char. 156: 0 --> 1
Char. 177: 2 --> 0

Paschatherium :
All trees:
Char. 10: 1 --> 0
Char. 11: 1 --> 0
Char. 16: 0 --> 1
Char. 22: 1 --> 0
Char. 29: 0 --> 1
Some trees:
Char. 7: 12 --> 3
Char. 24: 1 --> 0
Char. 28: 0 --> 1
Char. 38: 01 --> 2
Char. 47: 0 --> 1
Char. 52: 12 --> 3
Char. 77: 0 --> 1
Char. 80: 0 --> 1
Char. 82: 0 --> 12
Char. 89: 1 --> 0
Char. 92: 1 --> 0
Char. 109: 2 --> 0
Char. 115: 1 --> 0
Char. 116: 1 --> 2

Perissodactyla :
All trees:
Char. 83: 0 --> 1
Char. 123: 0 --> 1
Char. 134: 1 --> 0
Char. 137: 1 --> 0
Char. 138: 1 --> 0
Char. 161: 0 --> 1
Some trees:
Char. 129: 0 --> 1

Phenacodontidae :
All trees:
Char. 37: 0 --> 1
Char. 103: 0 --> 1
Some trees:
Char. 7: 23 --> 01
Char. 9: 0 --> 1
Char. 10: 1 --> 2
Char. 38: 1 --> 0
Char. 40: 0 --> 1
Char. 79: 0 --> 1
Char. 83: 0 --> 1
Char. 93: 1 --> 0
Char. 99: 0 --> 1
Char. 124: 0 --> 2
Char. 127: 1 --> 2
Char. 146: 0 --> 1
Char. 173: 0 --> 1

Phenacolophus :
All trees:
Char. 10: 1 --> 0
Char. 32: 0 --> 1
Char. 73: 0 --> 2
Char. 76: 1 --> 0
Char. 103: 0 --> 2
Char. 104: 1 --> 0
Char. 115: 1 --> 0
Some trees:
Char. 7: 12 --> 3
Char. 38: 1 --> 0
Char. 40: 0 --> 1
Char. 45: 1 --> 2
Char. 77: 2 --> 0
Char. 99: 0 --> 1

Phosphatherium :
All trees:
Char. 57: 2 --> 1
Char. 120: 0 --> 2
Char. 124: 0 --> 2
Char. 172: 1 --> 0

Potamogale :
All trees:
Char. 45: 1 --> 0
Char. 49: 01 --> 2
Char. 69: 1 --> 0
Char. 95: 0 --> 1
Char. 106: 0 --> 1
Char. 126: 0 --> 2
Char. 137: 12 --> 3
Char. 141: 01 --> 2
Char. 144: 0 --> 2
Char. 146: 0 --> 1
Char. 153: 0 --> 1
Char. 165: 0 --> 1
Char. 178: 0 --> 1
Some trees:
Char. 127: 1 --> 3
Char. 129: 0 --> 1
Char. 140: 0 --> 12
Char. 152: 0 --> 1
Char. 156: 0 --> 1
Char. 177: 02 --> 1

Protungulatum :
All trees:
Char. 27: 0 --> 1
Char. 35: 0 --> 1
Char. 45: 1 --> 0
Char. 46: 0 --> 2
Some trees:
Char. 31: 0 --> 1
Char. 94: 0 --> 1

Ptolemaia :
All trees:
Char. 7: 1 --> 0
Char. 26: 0 --> 2
Char. 38: 01 --> 2
Char. 47: 0 --> 1
Char. 48: 0 --> 2
Char. 51: 0 --> 1
Char. 82: 0 --> 1
Char. 92: 0 --> 1
Char. 93: 0 --> 1
Char. 95: 0 --> 3
Char. 104: 0 --> 1
Char. 105: 0 --> 1
Char. 120: 0 --> 2

Radinskya :
All trees:
Char. 82: 3 --> 1
Char. 143: 1 --> 0

Sirenia :
All trees:
Char. 14: 0 --> 1
Char. 19: 0 --> 1
Char. 30: 1 --> 0
Char. 42: 1 --> 2
Char. 46: 0 --> 2
Char. 53: 0 --> 1
Char. 73: 0 --> 2
Char. 75: 1 --> 02
Char. 76: 2 --> 0
Char. 93: 0 --> 2
Char. 132: 1 --> 2
Char. 140: 3 --> 5
Char. 141: 1 --> 0

Char. 145: 0 --> 1

Teilhardimys :
All trees:
Char. 16: 0 --> 1
Char. 20: 0 --> 1
Char. 22: 1 --> 0
Char. 25: 0 --> 2
Char. 29: 0 --> 1
Char. 52: 2 --> 3
Char. 73: 0 --> 1
Char. 74: 0 --> 2
Char. 76: 1 --> 2
Some trees:
Char. 21: 1 --> 0
Char. 28: 0 --> 1
Char. 30: 0 --> 1
Char. 35: 0 --> 1
Char. 79: 0 --> 12
Char. 82: 3 --> 2

Todralestes :
All trees:
Char. 9: 0 --> 1
Char. 51: 0 --> 2
Char. 75: 1 --> 0

Node 28 :
All trees:
Char. 35: 0 --> 1
Char. 39: 0 --> 1
Char. 42: 0 --> 1
Char. 104: 0 --> 1
Char. 105: 0 --> 1
Char. 107: 2 --> 3
Char. 110: 0 --> 1
Char. 111: 0 --> 1
Char. 112: 0 --> 1
Some trees:
Char. 140: 0 --> 2

Node 29 :
All trees:
Char. 57: 0 --> 12
Char. 60: 0 --> 1
Char. 174: 1 --> 2
Some trees:
Char. 9: 0 --> 12
Char. 12: 0 --> 12
Char. 30: 0 --> 1
Char. 33: 0 --> 1
Char. 34: 0 --> 1
Char. 37: 0 --> 1
Char. 44: 1 --> 2
Char. 84: 1 --> 2
Char. 107: 0 --> 2
Char. 130: 0 --> 1
Char. 142: 1 --> 0

Node 30 :
All trees:
Char. 7: 1 --> 3
Char. 16: 0 --> 1
Char. 63: 0 --> 1
Char. 64: 1 --> 2
Char. 68: 0 --> 1
Char. 94: 0 --> 1
Some trees:
Char. 49: 0 --> 01
Char. 141: 0 --> 01

Node 31 :
All trees:
Char. 10: 1 --> 3
Char. 70: 0 --> 1
Some trees:
Char. 58: 0 --> 1
Char. 64: 0 --> 1
Char. 128: 0 --> 1
Char. 137: 1 --> 2

Node 32 :
All trees:
No synapomorphies

Node 33 :
All trees:
Char. 10: 1 --> 0
Char. 42: 0 --> 2
Some trees:
Char. 38: 1 --> 0

Node 34 :
All trees:
Char. 15: 0 --> 1
Char. 17: 0 --> 1
Char. 135: 0 --> 1

Node 35 :
All trees:
Char. 29: 0 --> 1
Char. 76: 1 --> 2
Char. 110: 0 --> 1
Char. 113: 0 --> 1
Some trees:
Char. 7: 23 --> 1
Char. 28: 0 --> 1
Char. 35: 0 --> 1
Char. 91: 1 --> 2

Node 36 :
All trees:
Char. 25: 0 --> 1
Char. 27: 0 --> 1
Char. 33: 0 --> 1
Char. 34: 0 --> 1
Char. 39: 1 --> 2
Some trees:
Char. 47: 1 --> 2
Char. 107: 1 --> 2
Char. 116: 12 --> 3
Char. 118: 0 --> 1

Node 37 :
All trees:
Char. 97: 2 --> 3
Char. 112: 0 --> 1
Char. 117: 0 --> 1
Some trees:
Char. 39: 0 --> 1
Char. 47: 0 --> 1
Char. 80: 0 --> 1
Char. 82: 0 --> 3
Char. 90: 0 --> 1
Char. 111: 0 --> 1
Char. 126: 0 --> 2
Char. 143: 0 --> 1

Node 38 :
All trees:
Char. 84: 1 --> 2
Char. 104: 0 --> 1
Char. 105: 0 --> 1
Some trees:
Char. 58: 0 --> 1
Char. 92: 0 --> 1
Char. 97: 1 --> 2

Node 39 :
All trees:
Char. 43: 0 --> 1
Char. 44: 1 --> 2
Char. 57: 0 --> 12
Char. 85: 0 --> 1
Char. 95: 0 --> 2
Some trees:
Char. 94: 0 --> 01
Char. 109: 01 --> 2

Node 40 :
All trees:
Char. 97: 0 --> 1
Char. 98: 0 --> 1
Char. 107: 0 --> 1

Node 41 :
All trees:
Char. 16: 0 --> 1
Char. 44: 2 --> 0
Char. 113: 1 --> 0

Node 42 :
All trees:
Char. 37: 1 --> 0
Char. 60: 1 --> 2
Char. 131: 0 --> 1
Char. 132: 0 --> 1
Char. 180: 0 --> 2

Node 43 :
All trees:
Char. 39: 1 --> 2
Char. 88: 0 --> 1
Char. 95: 0 --> 23
Char. 140: 2 --> 3
Char. 155: 0 --> 1
Char. 157: 0 --> 1
Char. 162: 0 --> 1
Some trees:
Char. 71: 0 --> 1
Char. 142: 0 --> 1
Char. 150: 0 --> 1

Node 44 :
All trees:
Char. 24: 1 --> 2
Char. 25: 0 --> 2
Char. 27: 0 --> 1
Char. 28: 0 --> 1
Char. 29: 0 --> 1
Char. 47: 0 --> 2
Char. 77: 0 --> 1
Char. 82: 0 --> 2
Char. 107: 3 --> 4
Char. 108: 1 --> 2
Char. 118: 0 --> 1
Char. 119: 0 --> 1

Char. 121: 0 --> 1

Some trees:

Char. 9: 12 --> 0

Char. 165: 0 --> 1

Node 45 :

All trees:

Char. 3: 0 --> 1

Char. 7: 3 --> 4

Char. 20: 0 --> 1

Char. 82: 2 --> 1

Char. 93: 0 --> 1

Char. 101: 0 --> 1

Some trees:

Char. 6: 0 --> 1

Node 46 :

All trees:

Char. 18: 0 --> 1

Char. 26: 0 --> 1

Char. 106: 0 --> 1

Char. 120: 0 --> 1

Some trees:

Char. 36: 0 --> 1

Char. 77: 02 --> 1

Char. 111: 1 --> 2

Char. 116: 12 --> 2

Node 47 :

All trees:

Char. 3: 1 --> 2

Char. 6: 1 --> 2

Char. 7: 4 --> 5

Char. 24: 2 --> 3

Char. 32: 0 --> 1

Char. 34: 1 --> 2

Char. 35: 1 --> 0

Char. 45: 1 --> 2

Char. 46: 0 --> 12

Char. 87: 0 --> 1

Char. 90: 1 --> 3

Char. 111: 1 --> 3

Char. 140: 3 --> 4

Char. 145: 0 --> 1

Node 48 :

All trees:

Char. 90: 1 --> 2

Char. 111: 1 --> 2

Char. 116: 3 --> 1

Char. 121: 1 --> 0

8. Analysis 8 : Hypocone (character 98) X 5, 44 characters additive, and implied weighting analysis

8.1 Cladogram 8

Traditional search, 1 tree found.

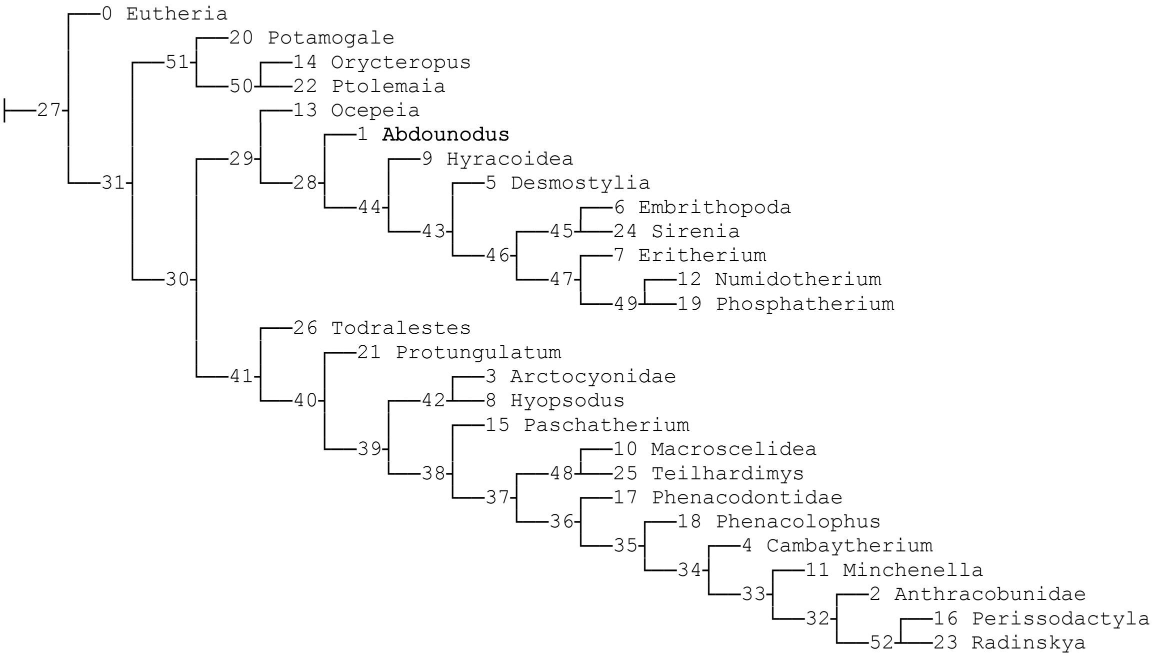

Node 29 = **Paenungulatomorpha**
Trees length : 768. Retention index: 56.0. Consistency Index: 37.0.

9. Analysis 9 : Analysis without Desmostylia, 44 characters additive, and unweighted analysis

This analysis was developed to check the effects of the long branch attraction of the Desmostylia (hypothesis of convergences of Desmostylia and Paenungulata)

44 characters additive.

26 active, 1 inactive taxa

26 terminal taxa are active:

0 Eutheria
1 Abdounodus
2 Anthracobunidae
3 Arctocyonidae
4 Cambaytherium
6 Embrithopoda
7 Eritherium
8 Hyopsodus
9 Hyracoidea
10 Macroscelidea
11 Minchenella
12 Numidotherium
13 Ocepeia
14 Orycteropus
15 Paschatherium
16 Perissodactyla
17 Phenacodontidae
18 Phenacolophus
19 Phosphatherium
20 Potamogale
21 Protungulatum
22 Ptolemaia
23 Radinskya
24 Sirenia
25 Teilhardimys
26 Todralestes

1 terminal taxa are inactive:

5 Desmostylia

9.1 Cladogram 9

Traditional search, 18 treew found.

Strict consensus of 18 trees (1 taxa excluded)

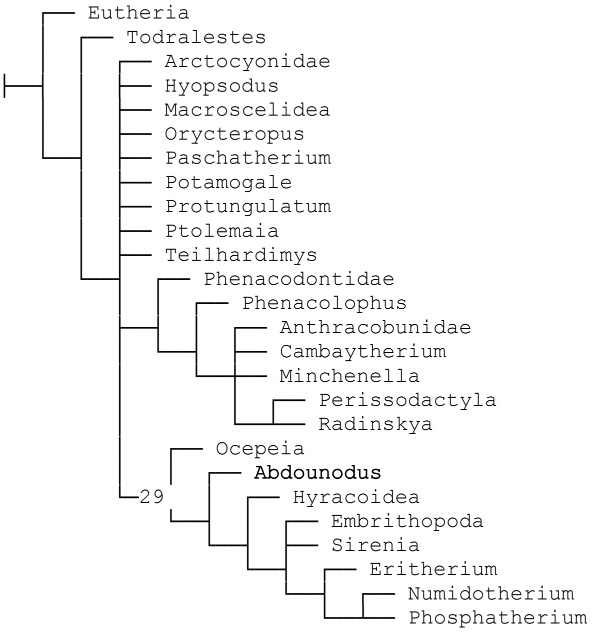

Node 29 = **Paenungulatomorpha**

Tree length : 711. Retention index: 55.5. Consistency Index: 38.3.

9.2 Bremer supports (from 10000 trees, cut 0)

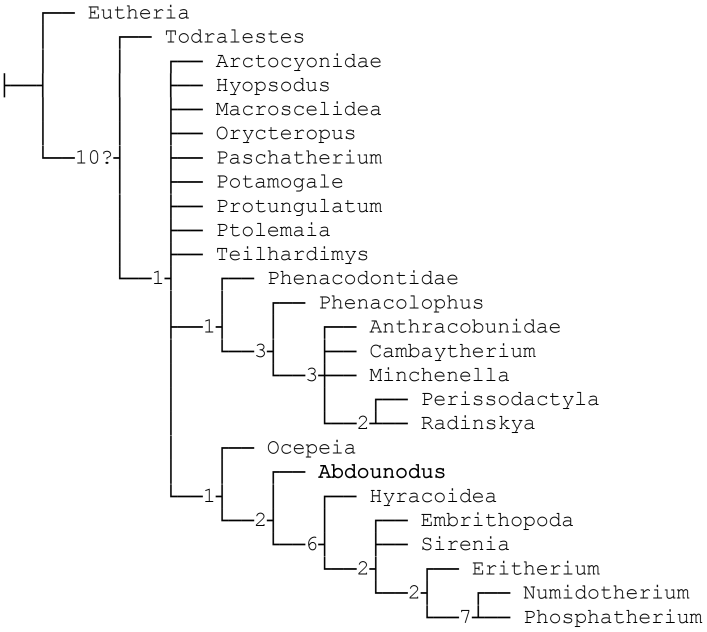

10. Analysis 10 : analysis without Desmostylia, 44 characters additive and implied weigthing analysis

This analysis was developed to check possible effects of long branch attraction of the Desmostylia (hypothesis of convergences of Desmostylia and Paenungulata).

10.1 Cladogram 10

Standard weighting strength is 3.00000.

Traditional search, 1 tree found.

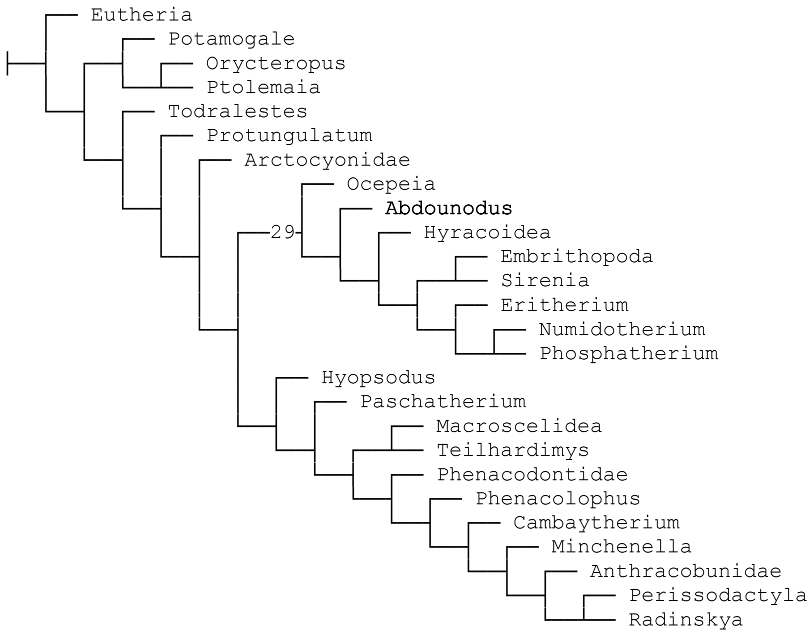

Node 29 = **Paenungulatomorpha**

Tree length : 718. Retention index: 54.8. Consistency Index: 37.9.
